# Effect of a Co-Located Bridging Recovery Initiative on Hospital Length of Stay Among Patients With Opioid Use Disorder: The BRIDGE Randomized Clinical Trial

**DOI:** 10.1001/jamanetworkopen.2023.56430

**Published:** 2024-02-27

**Authors:** David Marcovitz, Mary Lynn Dear, Rebecca Donald, David A. Edwards, Kristopher A. Kast, Thao D. V. Le, Mauli V. Shah, Jason Ferrell, Cheryl Gatto, Cassandra Hennessy, Reagan Buie, Todd W. Rice, William Sullivan, Katie D. White, Grace Van Winkle, Rachel Wolf, Christopher J. Lindsell

**Affiliations:** 1 Vanderbilt University School of Medicine, Nashville, Tennessee; 2 Department of Psychiatry and Behavioral Sciences, Vanderbilt University School of Medicine, Nashville, Tennessee; 3 Vanderbilt Institute for Clinical and Translational Research, Vanderbilt University Medical Center, Nashville, Tennessee; 4 Division of Pain Medicine, Department of Anesthesia, Vanderbilt University Medical Center, Nashville, Tennessee; 5 Department of Biostatistics, Vanderbilt University School of Medicine, Nashville, Tennessee; 6 Office of Episodes of Care, Vanderbilt University Medical Center, Nashville, Tennessee; 7 Division of Allergy, Pulmonary, and Critical Care Medicine, Vanderbilt University Medical Center, Nashville, Tennessee; 8 Division of General Internal Medicine and Public Health, Vanderbilt University Medical Center, Nashville, Tennessee; 9 Division of Infectious Diseases, Vanderbilt University Medical Center, Nashville, Tennessee

## Abstract

**Importance:**

Co-located bridge clinics aim to facilitate a timely transition to outpatient care for inpatients with opioid use disorder (OUD); however, their effect on hospital length of stay (LOS) and postdischarge outcomes remains unclear.

**Objective:**

To evaluate the effect of a co-located bridge clinic on hospital LOS among inpatients with OUD.

**Design, Setting, and Participants:**

This parallel-group randomized clinical trial recruited 335 adult inpatients with OUD seen by an addiction consultation service and without an existing outpatient clinician to provide medication for OUD (MOUD) between November 25, 2019, and September 28, 2021, at a tertiary care hospital affiliated with a large academic medical center and its bridge clinic.

**Intervention:**

The bridge clinic included enhanced case management before and after hospital discharge, MOUD prescription, and referral to a co-located bridge clinic. Usual care included MOUD prescription and referrals to community health care professionals who provided MOUD.

**Main Outcomes and Measures:**

The primary outcome was the index admission LOS. Secondary outcomes, assessed at 16 weeks, were linkage to health care professionals who provided MOUD, MOUD refills, same-center emergency department (ED) and hospital use, recurrent opioid use, quality of life (measured by the Schwartz Outcome Scale–10), overdose, mortality, and cost. Analysis was performed on an intent-to-treat basis.

**Results:**

Of 335 participants recruited (167 randomized to the bridge clinic and 168 to usual care), the median age was 38.0 years (IQR, 31.9-45.7 years), and 194 (57.9%) were male. The median LOS did not differ between arms (adjusted odds ratio [AOR], 0.94 [95% CI, 0.65-1.37]; *P* = .74). At the 16-week follow-up, participants referred to the bridge clinic had fewer hospital-free days (AOR, 0.54 [95% CI, 0.32-0.92]), more readmissions (AOR, 2.17 [95% CI, 1.25-3.76]), and higher care costs (AOR, 2.25 [95% CI, 1.51-3.35]), with no differences in ED visits (AOR, 1.15 [95% CI, 0.68-1.94]) or deaths (AOR, 0.48 [95% CI, 0.08-2.72]) compared with those receiving usual care. Follow-up calls were completed for 88 participants (26.3%). Participants referred to the bridge clinic were more likely to receive linkage to health care professionals who provided MOUD (AOR, 2.37 [95% CI, 1.32-4.26]) and have more MOUD refills (AOR, 6.17 [95% CI, 3.69-10.30]) and less likely to experience an overdose (AOR, 0.11 [95% CI, 0.03-0.41]).

**Conclusions and Relevance:**

This randomized clinical trial found that among inpatients with OUD, bridge clinic referrals did not improve hospital LOS. Referrals may improve outpatient metrics but with higher resource use and expenditure. Bending the cost curve may require broader community and regional partnerships.

**Trial Registration:**

ClinicalTrials.gov Identifier: NCT04084392

## Key Points

**Question:**

Does referring inpatients with opioid use disorder (OUD) from a hospital addiction consultation service to a co-located outpatient bridge clinic have an effect on hospital length of stay (LOS) compared with usual care?

**Findings:**

In this randomized clinical trial including 335 patients, with only 88 [26.3%] available for follow-up, bridge clinic referral did not affect hospital LOS. Patients referred to the bridge clinic had more buprenorphine refills, improved linkage to health care professionals who provide medication for OUD, and reduced overdose rates based on 16-week self-report after discharge but reported no significant difference in recurrent opioid use, death rates, or quality of life compared with usual care.

**Meaning:**

Referring inpatients with OUD to a co-located outpatient bridge clinic did not affect hospital LOS.

## Teaser

This randomized clinical trial evaluates the effect of a co-located bridge clinic on hospital length of stay among inpatients with opioid use disorder.

## Introduction

The number of overdose deaths in the US reached 105 452 in 2022, an increase of 48% compared with 2019.^1^ Although substance use disorder (SUD)–related admissions already represented 20% to 30% of general hospital admissions,^2,3,4^ the health care system has witnessed concomitant increases in hospitalizations associated with complex, injection drug–related infections, sometimes prolonged due to difficulty placing patients for parenteral antibiotics.^5^ Given the connection between such admissions and increased cost, length of stay (LOS), morbidity, and mortality,^6,7^ there is significant focus on integration of SUD care into the general hospital.^8^

Multiple components of SUD treatment have demonstrated effectiveness in the outpatient setting, none more than medication for opioid use disorder (MOUD), including methadone and buprenorphine-naloxone.^9^ Integration of motivational interviewing, comprehensive case management (where patients receive enhanced support for managing other medical appointments and support with their social determinants of health), and MOUD into the general outpatient medical setting has shown promise.^10^

The past decade has witnessed additional research on the role of an integrated addiction consultation service (ACS) in general hospitals,^11,12,13,14,15,16,17,18,19^ with early positive indicators for LOS,^16,17^ postdischarge addiction severity,^12^ and readmission.^15,19^ Bridge clinics, generally described as transitional outpatient clinics designed to provide wraparound care while a patient transitions from inpatient to outpatient settings, have proven feasible to implement and offer promise in supporting longitudinal engagement.^20,21,22^ Bridge clinics offer presumed care advantages, including timely provision of MOUD while a long-term clinician is identified, and notwithstanding other barriers, including stigma, they may offer the ability to discharge patients early with continued outpatient parenteral antibiotic therapy (OPAT), which could directly affect the hospital LOS. Hence, LOS may serve as a measurable surrogate for timely linkage to treatment and thus as a measure for benefits of the bridge clinic model. Despite putative benefits, to our knowledge, there are no known randomized clinical trials systematically evaluating the effect of referring inpatients from an ACS to a bridge clinic on LOS and postdischarge outcomes. This study was designed to test the hypothesis that among individuals with OUD receiving inpatient addiction consultations, more active coordination and execution of aftercare in the context of a co-located bridge clinic model would reduce hospital LOS and improve patient-centered outcomes compared with usual care.

## Methods

### Study Design

The Bridging Recovery Initiative Despite Gaps in Entry (BRIDGE) trial^23^ (NCT04084392) was a single-center, pragmatic (ie, comparative effectiveness embedded into routine process of care) parallel-group, randomized clinical trial designed to evaluate the clinical effectiveness of referring patients to the bridge clinic as a part of clinical care (study protocol, statistical analysis plan, and statistical analysis report in Supplement 1). Patients in both study arms maintained autonomy relative to decisions about their care. This study was approved by the Vanderbilt University institutional review board with a waiver of informed consent (it is not practicable to answer the research question with informed consent because patients who choose to participate will necessarily differ from those who do not in substantial ways, including access to care). This study followed the Consolidated Standards of Reporting Trials (CONSORT) reporting guideline.^24^

### Participants

Hospitalized adults with OUD seen in an ACS were screened for enrollment between November 25, 2019, and September 28, 2021. The inclusion criteria were (1) aged 18 years or older, (2) active OUD and accepting a transitional prescription for buprenorphine-naloxone or intramuscular naltrexone injection, and (3) outpatient MOUD plans not fixed prior to admission. Exclusion criteria were (1) ACS deemed ineligible for referral to outpatient bridge clinic or (2) previous randomization in this study. Patients referred to a bridge clinic prior to study initiation remained in bridge clinic care and were not screened. Ineligibility criteria determined by the ACS included, but were not limited to, severe, active co-occurring psychiatric disorders requiring a higher level of psychiatric care, and methadone maintenance was deemed the best choice of MOUD.

### Assessment at Baseline

The ACS was notified of potentially eligible patients via a consultation order from the patient’s primary hospital service. An ACS clinician evaluated patients for OUD, while an ACS social worker screened the patient and proceeded with randomization once eligibility criteria were met.

Some patients were admitted with infections related to injection drug use, requiring several weeks of treatment with intravenous antibiotics. We leveraged a protocol to assess candidacy for OPAT among patients randomized to the bridge clinic. However, due to hospital bed demand related to the COVID-19 pandemic, this protocol was modified such that all patients deemed eligible for OPAT were referred to a bridge clinic and were not included in the trial after April 2021.^23^

### Randomization

Randomization was performed using the randomization module in REDCap.^25^ At study outset, the bridge clinic had the capacity to accept approximately 50% of eligible patients. We used randomization to determine patient referral, and allocation was set at a 1:1 ratio.

### Bridge Clinic Intervention

The bridge clinic patients were informed that they would be referred to a co-located, multispecialty clinic with wraparound services for patients with OUD before and after hospital discharge using shared, co-located staff with the inpatient ACS.^23^ The care team included 4 medical specialties (addiction psychiatry, internal medicine, infectious diseases, and pain anesthesia), social workers, recovery coaches, and a nurse case manager.

Patients were provided a buprenorphine-naloxone bridge prescription, generally in the range of 12 to 16 mg, and on the first Friday after discharge, patients saw a medical professional based on their condition; other disciplines rotated seeing the patient as needed. Patients were asked to present weekly to the clinic for the first 8 weeks of treatment, then twice monthly based on their clinical presentation and stability (assuming they maintained abstinence from opioids, alcohol, benzodiazepines, and stimulants). The target period for stabilization and transition to a long-term treatment program was no more than 3 months, although, in practice, this period could have been as long as 1 year. Permanent transition into appropriate long-term care was addressed as often as every visit. Care was provided at no cost for patients lacking insurance.

### Usual Care

Patients randomized to usual care were referred to a community health care professional for MOUD linkage by a nonspecialty, hospital-based social worker team. The community referrals were generally requested between 7 and 10 days after discharge and were all located within a 15- to 20-minute drive from Vanderbilt University Medical Center. The ACS initiated MOUD in a dosage range of 12 to 16 mg, addressed psychiatric comorbidity during the hospitalization, and provided prescriptions to last until linkage to MOUD. However, they did not receive further OUD aftercare–related case management or recovery coaching in the hospital, they had no planned touchpoints with ACS or the bridge clinic after discharge, and there was no comprehensive case management (where patients receive enhanced support for managing other medical appointments and support with their social determinants of health).

### Outcomes

The primary outcome was the overall LOS from admission to hospital discharge. The self-reported secondary outcomes, assessed for the 16-week follow-up period, were linkage to health care professionals who provided MOUD, buprenorphine-naloxone (or naltrexone) prescriptions filled, recurrent opioid use, overall quality of life as measured by the Schwartz Outcome Scale–10, and overdose. The electronic medical record–based secondary outcomes, assessed for the 16-week follow-up period, were same-center emergency department (ED) visit and hospital readmission rates, hospital-free days, ED-free days, mortality, and care costs.

Exploratory outcomes for patients suitable for OPAT included new or recurrent infection (as defined by a positive culture result and/or change in antibiotic regimen), antibiotic therapy completion, and number of days from negative blood culture result to first hospital discharge. Implementation and fidelity measurements included acceptance of a referral to a bridge clinic and intervention contamination, as well as reasons for ineligibility. Intervention contamination was defined as when a patient randomized to usual care was offered the bridge clinic or when a patient was randomized to the bridge clinic but was offered only usual care at the time of discharge from the inpatient stay.

### Statistical Analysis

The analysis for this trial used an intent-to-treat approach (eAppendix in Supplement 2). Descriptive statistics were used to summarize participant baseline characteristics and outcomes, overall and by study arm. Categorical variables were summarized as counts and percentages, and continuous variables were summarized as median (IQR) values. Since the main outcome data are positive and skewed, a proportional odds model was used to compare LOS between those offered referral to the bridge clinic and those receiving usual care on an intent-to-treat basis. The model was adjusted for age, race, ethnicity, and social deprivation quartile. The race and ethnicity data were obtained from Research Derivative, which is derived from the Vanderbilt University Medical Center’s electronic medical record. The researchers did not determine race or ethnicity; they were abstracted from the electronic medical record. This information is self-reported and/or input by clinical personnel. These variables were collected as baseline demographics to characterize the study population. The Vanderbilt University Medical Center is committed to addressing issues of diversity, equity, and inclusion and is currently engaged in systemwide initiatives to remediate historic inequities in care, in part by hiring a more diverse workforce and also by collecting relevant data in the course of routine research that can inform the equitable distribution of health care resources.

The Area Deprivation Index (ADI) was estimated using the Neighborhood Atlas tool.^26,27^ The ADI is calculated with 17 indicators from the American Community Survey encompassing income, educational level, employment, and housing conditions and can show where areas of deprivation and affluence exist within a community divided into social deprivation quartiles, which is a 4-level ordinal indicator (highest quartiles show greatest deprivation). Multiple imputation using estimated mean matching was used to impute missing data. To account for the uncertainty in specifying the start point for LOS, a post hoc sensitivity analysis was used to compare the LOS between study arms from the point of randomization to hospital discharge during the index inpatient visit. Differential treatment effects were assessed by testing the interaction between the study arm and baseline characteristic of interest in the proportional odds model. A *P* value of .20 was determined a priori to suggest the possibility of a differential effect for exploratory outcomes.

Secondary and exploratory outcomes were adjusted for the same covariates as the primary model. Continuous and ordinal outcomes were modeled using proportional odds models, and binary outcomes were modeled using logistic regression. Single imputation used modes for categorical variables and mean values for continuous variables. Adjustments for multiplicity were not made, and observed effects should be interpreted as exploratory. All analyses were conducted using R, version 4.2.2 (R Project for Statistical Computing), including the Hmisc, rms, and survey packages. All *P* values were from 2-sided tests, and results were deemed statistically significant at *P* < .05.

Prior to the bridge clinic establishment, the mean (SD) LOS for patients meeting inclusion criteria was 15 (15) days (range, 3-42 days). We estimated that 350 patients randomized to each study arm would provide greater than 80% power to detect a reduction in LOS of 3 days. Sample size was reestimated midway through the recruitment period on July 27 and August 8, 2020, due to disruption caused by the COVID-19 pandemic and low enrollment (136 patients). The distribution of LOS was estimated for enrolled patients (mean [SD] LOS, 9 [11] days). We estimated that 168 patients per study arm would provide 80% power to detect a reduction in LOS of 1.5 days.

## Results

### Study Participants

**Figure 1.**
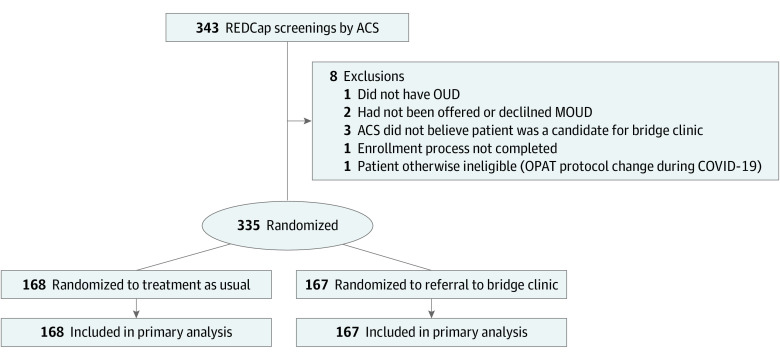
CONSORT Diagram of Participant Flow Through the Trial ACS indicates addiction consultation service; MOUD, medication for opioid use disorder; OPAT, outpatient parenteral antibiotic therapy; and OUD, opioid use disorder.

Of 343 patients initially identified as potential candidates and referred for screening, 335 were randomized (167 to the bridge clinic and 168 to usual care). Reasons for exclusion were as follows: the patient did not have OUD (n = 1), the patient was not offered or declined MOUD (n = 2), ACS did not believe the patient was a candidate for the bridge clinic (n = 3), the enrollment process was not completed (n = 1), and the patient was found to be otherwise ineligible due to changes in the OPAT protocol during the COVID-19 pandemic (n = 1) (Figure 1). Seven participants were deemed eligible for OPAT. Although patients in the usual care arm were not eligible for OPAT, 1 patient in the usual care arm was mistakenly screened for OPAT and referred to the bridge clinic for OPAT. This patient was analyzed as usual care per the intent-to-treat analysis. Of the 167 participants assigned to the bridge clinic, 97 (58.1%) attended at least 1 visit at the bridge clinic during the 16-week follow-up period. One participant assigned to the usual care arm was exposed to the bridge clinic during this time (eAppendix in Supplement 2).

### Demographic and Baseline Characteristics

In the overall cohort of 335 participants, the median age was 38.0 years (IQR, 31.9-45.7 years), and 194 (57.9%) were male (Table 1). The study participants had a mean ADI of 55.7 (range, 41.5-72.0). For 38 patients, the ADI value^26^ could not be captured due to no record of their home address (n = 1), an indication of homelessness (n = 4), or the ADI value being suppressed due to high group quarters population (ie, transitional housing) (n = 33). Demographic variables, including social deprivation,^27^ were balanced across the study arms.

**Table 1.** Participant Demographic Characteristics

Characteristic	Participants, No. (%)		
	Total (N = 335)	Bridge clinic (n = 167)	Usual care (n = 168)
Age, median (IQR), y	38.0 (31.9-45.7)	38.7 (32.1-47.0)	37.9 (31.8-44.5)
Sex			
Female	141 (42.1)	72 (43.1)	69 (41.1)
Male	194 (57.9)	95 (56.9)	99 (58.9)
Race			
Black or African American	36 (10.7)	16 (9.6)	20 (11.9)
White	287 (85.7)	144 (86.2)	143 (85.1)
Other^a^	10 (3.0)	6 (3.6)	4 (2.4)
Missing or unknown	2 (0.6)	1 (0.6)	1 (0.6)
Ethnicity			
Hispanic or Latino	12 (3.6)	6 (3.6)	6 (3.6)
Non-Hispanic or non-Latino	320 (95.5)	161 (96.4)	159 (94.6)
Missing or unknown	3 (0.9)	0	3 (1.8)
Social deprivation (quartile)^b^			
Quartile 1	112 (33.4)	55 (32.9)	57 (33.9)
Quartile 2	73 (21.8)	35 (21.0)	38 (22.6)
Quartile 3	71 (21.2)	35 (21.0)	36 (21.4)
Quartile 4	79 (23.6)	42 (25.1)	37 (22.0)

^a^ Includes Asian, declined to answer, mixed race, and those listed as “other.”

^b^ Social deprivation incorporated the Census Tract Area Deprivation Index where available, and data were extrapolated from the electronic medical record. Higher quartiles indicate greater deprivation; quartile 4 is the highest deprivation.

### Primary Outcome

**Figure 2.**
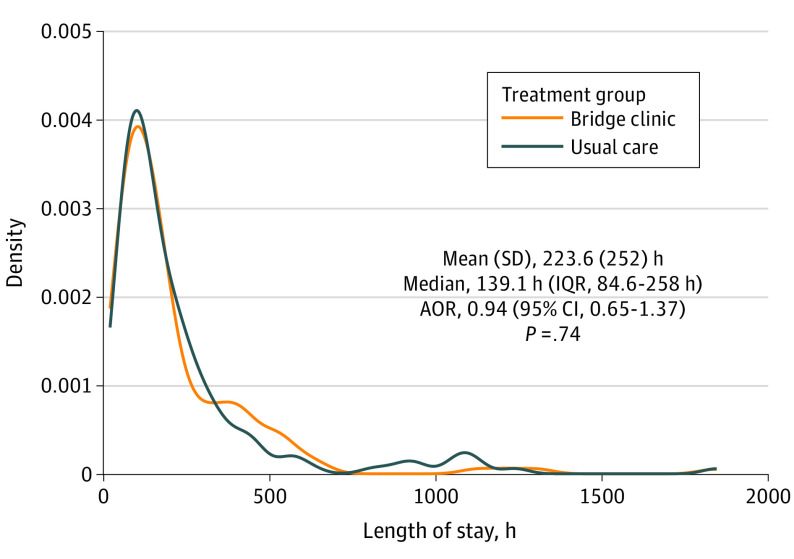
Primary Outcome: Length of Stay by Study Group AOR indicates adjusted odds ratio.

Index hospital LOS from admission to discharge was not shown to differ between the 2 arms (median, 5.7 days [IQR, 3.6-10.7 days] in the usual care group and 5.9 days [IQR, 3.3-11.5 days] in the bridge clinic group; adjusted odds ratio [AOR], 0.94 [95% CI, 0.65-1.37]; *P* = .74) (Figure 2). A similar result was observed for a post hoc sensitivity analysis comparing LOS from the point of randomization to hospital discharge between study arms (eAppendix in Supplement 2).

### Other Outcomes

In the 16 weeks after enrollment, participants in the bridge clinic arm experienced fewer hospital-free and ED-free days (AOR, 0.61 [95% CI, 0.39-0.95]), more readmissions or ED visits (AOR, 1.66 [95% CI, 1.06-2.60]), fewer hospital-free days (AOR, 0.54 [95% CI, 0.32-0.92]), more readmissions (AOR, 2.17 [95% CI, 1.25-3.76]), and higher care costs (AOR, 2.25 [95% CI, 1.51-3.35]), with no differences in ED visits (AOR, 1.15 [95% CI, 0.68-1.94]) or deaths (AOR, 0.48 [95% CI, 0.08-2.72]) compared with those receiving usual care (Table 2). Total median costs of care were higher among those referred to the bridge clinic than those in the usual care arm ($9482 [IQR, $1478-$29 376] vs $1705 [IQR, $360-$12 146]; AOR, 2.25 [95% CI, 1.51-3.35]). Mortality rates were similar between groups. A total of 22 patients in the bridge arm and 17 patients in the usual care arm were discharged before medically advised (BMA).

**Table 2.** Electronic Medical Record–Based Outcomes

Outcome	Total (N = 335)	Bridge clinic (n = 167)	Usual care (n = 168)	AOR (95% CI)
Composite measures				
Hospital-free and ED-free days, median (IQR)	112.00 (110.00-112.00)	112.00 (107.00-112.00)	112.00 (111.00-112.00)	0.61 (0.39-0.95)
No. of readmissions or ED visits				
Mean (SD)	0.76 (1.63)	0.92 (1.82)	0.59 (1.40)	1.66 (1.06-2.60)
Median (IQR)	0 (0-1)	0 (0-1)	0 (0-1)	
At least 1 readmission or ED visit, No. (%)^a^	121 (36.1)	69 (41.3)	52 (31.0)	1.61 (1.01-2.57)
Readmissions				
Hospital-free days, median (IQR)	112.00 (112.00-112.00)	112.00 (109.00-112.00)	112.00 (112.00-112.00)	0.54 (0.32-0.92)
No. of readmissions				
Mean (SD)	0.33 (0.82)	0.46 (1.02)	0.20 (0.53)	2.17 (1.25-3.76)
Median (IQR)	0 (0-0)	0 (0-1)	0 (0-0)	
At least 1 readmission, No. (%)^a^	70 (20.9)	45 (26.9)	25 (14.9)	2.10 (1.21-3.67)
ED visits				
No. of ED visits				
Mean (SD)	0.43 (1.24)	0.47 (1.33)	0.39 (1.15)	1.15 (0.68-1.94)
Median (IQR)	0 (0-0)	0 (0-0)	0 (0-0)	
At least 1 ED visit, No. (%)^a^	76 (22.7)	40 (24.0)	36 (21.4)	1.17 (0.69-1.97)
Other measures				
Total cost of care, median (IQR), $	4926.93 (678.99-22 109.90)	9481.93 (1478.28-29 376.14)	1705.28 (360.16-12 146.33)	2.25 (1.51-3.35)
Missing	24 (7.2)	7 (4.2)	17 (10.1)	
Death, No. (%)				
Yes	6 (1.8)	2 (1.2)	4 (2.4)	0.48 (0.08-2.72)

Abbreviations: AOR, adjusted odds ratio; ED, emergency department.

^a^ Post hoc analysis.

Follow-up calls were completed for 88 participants (26.3%). To account for a difference in follow-up rates between study arms (63.6% bridge clinic [56 participants]; 36.4% usual care [32 participants]), an inverse probability of treatment (follow-up) weighting analysis was conducted for self-reported secondary outcomes. Propensity for follow-up was estimated from baseline characteristics (age, race, ethnicity, and social deprivation). Although patients referred to the bridge clinic were more likely to report opioid use within the past 30 days (ie, weeks 12-16 after hospital discharge) (AOR, 46.94 [95% CI, 9.28-237.35]), recurrent opioid use over the full 16-week follow-up did not differ between arms, and participants referred to the bridge clinic were less likely to report experiencing an overdose (AOR, 0.11 [95% CI, 0.03-0.41) (Table 3). Linkage to health care professionals who provided MOUD was more frequent (AOR, 2.37 [95% CI, 1.32-4.26]), and the number of buprenorphine fills was higher (AOR, 6.17 [95% CI, 3.69-10.30]) among those referred to the bridge clinic compared with usual care. Quality of life as measured by the Schwartz Outcome Scale–10 was not significantly different between the 2 groups.

**Table 3.** Self-Reported Outcomes at 16-Week Follow-Up

Outcome	Participants, No. (%)			Propensity score, AOR (95% CI)^a^
	Total (N = 335)	Bridge clinic (n = 167)	Usual care (n = 168)	
Opioid use within past 30 d	NA	NA	NA	46.94 (9.28-237.35)
No use	4 (1.2)	2 (1.2)	2 (1.2)	NA
Single use	4 (1.2)	0	4 (2.4)	NA
Multiple use	24 (7.2)	17 (10.2)	7 (4.2)	NA
Unable to recontact at 16 wk	303 (90.4)	148 (88.6)	155 (92.3)	NA
Recurrent opioid use	NA	NA	NA	0.70 (0.42-1.18)
Yes	38 (11.3)	23 (13.8)	15 (8.9)	NA
No	50 (14.9)	33 (19.8)	17 (10.1)	NA
Unable to recontact at 16 wk	247 (73.7)	111 (66.5)	136 (81.0)	NA
Overdose	NA	NA	NA	0.11 (0.03-0.41)
Yes	5 (1.5)	1 (0.6)	4 (2.4)	NA
No	81 (24.2)	53 (31.7)	28 (16.7)	NA
Unable to recontact at 16 wk	249 (74.3)	113 (67.7)	136 (81.0)	NA
Linkage to health care professional who provided MOUD	NA	NA	NA	2.37 (1.32-4.26)
Yes	64 (19.1)	45 (26.9)	19 (11.3)	NA
No	24 (7.2)	11 (6.6)	13 (7.7)	NA
Unable to recontact at 16 wk	247 (73.7)	111 (66.5)	136 (81.0)	NA
No. of buprenorphine fills, median (IQR)	6.0 (0.0-15.0)	10.0 (4.0-16.0)	1.0 (0.0-6.0)	6.17 (3.69-10.30)
Missing	258 (77.0)	121 (72.5)	137 (81.5)	NA
Quality of Life SOS10 score, median (IQR)	44.0 (29.5-54.0)	47.0 (26.0-53.8)	41.5 (32.5-54.3)	0.90 (0.58-1.38)
Missing	249 (74.3)	113 (67.7)	136 (81.0)	NA

Abbreviations: AOR, adjusted odds ratio; MOUD, medication for opioid use disorder; NA, not applicable; SOS10, Schwartz Outcome Scale–10.

^a^ Calculated for 16-week self-reported outcomes.

For the 7 patients deemed eligible to receive OPAT, none experienced a new or recurrent infection, and 6 completed the antibiotic therapy regimen. Data on the time from negative blood culture result to hospital discharge were available for 5 of the 7 participants (median time, 15 hours [IQR, 12-21 hours]) (eAppendix in Supplement 2).

## Discussion

In this pragmatic randomized clinical trial, referring patients initiating MOUD in a general hospital to a multidisciplinary bridge clinic was not shown to reduce hospital LOS compared with usual care. When compared with usual care, referral to the bridge clinic increased hospital readmission and the related overall cost of postdischarge care. Among the 26.3% of all trial enrollees who could be reached at 16 weeks after discharge, those referred to the bridge clinic had increased buprenorphine refills, improved linkage to health care professionals who provided MOUD, reduced overdose rates, and no change in recurrent opioid use, death rates, or quality of life after accounting for propensity to follow-up. The bridge clinic appeared to be an acceptable treatment option, with 58.1% of those referred attending at least 1 visit.

Although retrospective studies of bridge clinic interventions have examined ED use and hospital readmissions,^20,21^ to our knowledge, this is the first randomized clinical trial to evaluate an intervention involving an ACS-to-bridge clinic referral pathway prospectively. As ACS intervened in both arms, it may have also reduced LOS for the usual care arm, masking potential effects of referral to the internal bridge clinic. We speculate that there is a complex relationship between referral to ACS-to-bridge services, rates of BMA discharge, and LOS; ACS may extend LOS (by decreasing BMA discharge) through drawing attention to the needs of a historically stigmatized population while simultaneously decreasing LOS when aftercare is proactively rendered. Among our participants, 22 in the bridge arm and 17 in the usual care arm were discharged BMA. If ACS consultations were enriched for patients with more complex conditions overall, inherently high rates of BMA discharge could further reduce LOS and mask the effects of the bridge clinic intervention.

Referral to the bridge clinic increased hospital readmissions and total cost of care within our medical center. Two prior retrospective studies have found lower 30-day^19^ and 90-day readmissions^15^ for those receiving ACS. Another study found no significant association between addiction consultation and 30-day use of acute care.^18^ One randomized clinical trial examined the benefit of including case management alongside addiction consultation for patients with SUD and found reduced 30-day readmission using linkage procedures to the surrounding community.^28^ In contrast, the combined effect of linked inpatient-outpatient support in our intervention may have led to increased patient affiliation to our medical center. Reduction in readmissions or total costs may have only accrued outside the 16-week window or when compared with readmissions across the multiple regional hospitals; such data were unavailable at the time of this publication. Recent analyses of claims data suggest that gaps in buprenorphine care are associated with increased total Medicare spending.^29^ It is also plausible that patients afforded access to addiction treatment services may have taken a greater interest in their health and were more likely to proactively seek help for emergency medical issues. As cost benefits accrue downstream, a comprehensive understanding of the cost-effectiveness of the referral strategy is warranted to argue for reasonable reimbursement based on global patient outcomes.

Nearly 60% of patients referred for bridge clinic services attended their first clinic visit. Because the response rate for patient-reported outcomes at the 16-week assessment was low and reduced certainty in comparisons between study arms, the inverse probability of treatment weighting approach was used to assist with causal inferences. Buprenorphine refills and linkage to health care professionals who provided MOUD were both improved in the group referred to the bridge clinic, as were self-reported overdose rates. These data must be interpreted with caution given the significant amount of missing data. The STOP (Suboxone Transition to Opiate Program) Trial found that offering buprenorphine linkage increased postdischarge clinic attendance compared with offering detoxification,^11^ although not for persons who inject drugs.^30^ Another large ED-based trial of buprenorphine linkage showed improvement in 30-day linkage among the intervention group, with lower self-reported recurrent opioid use not corroborated by urine toxicology results.^31^ In our trial, in contrast to these studies, we required usual care inpatients to receive buprenorphine induction and discharge prescriptions to community health care professionals within 7 to 10 days. This proactive approach to usual care—an emerging standard of care during the trial—may have influenced our secondary outcomes.

We expected a major benefit of the bridge clinic would be to allow for early OPAT. However, due to our strict inclusion criteria, the effect of the COVID-19 pandemic, and the eventual decision to exclude patients, there were only 7 trial patients eligible for OPAT. Prior literature suggests that OPAT may be safe and effective for persons who inject drugs compared with those with no history of injection drug use.^32^ However, some studies required minimum standards for OPAT inclusion^33,34^ that we sought to replicate, including acceptance of MOUD, stable housing, and a sober support person, among others. Conclusions regarding the effect of referral to the bridge clinic on outcomes for patients eligible for OPAT should not be made based on our limited data.

This trial began 6 months after the launch of the bridge clinic while it inhabited a temporary space. Although 58.1% of patients referred to the bridge clinic had at least 1 visit, procedures for collaboration with primary hospital teams, specialty clinician-patient pairing, case management, and recovery coach interactions were evolving during this time. In contrast, the SUMMIT (Substance Use, Motivation and Medication Integrated Treatment) trial, a health services delivery intervention for SUD, used a nearly 2-year implementation process prior to trial launch to achieve the system-level change needed to support the intervention.^35^ Our approach sought to generate early evidence on local effectiveness to inform continued hospital investment in the bridge clinic intervention, recognizing that rapid Plan-Do-Study-Act cycles are common in improving clinical processes even in mature systems.

In terms of clinical significance, we enrolled a highly representative sample of patients by embedding recruitment within normal workflows to maximize inclusivity. Participants in the study had a mean ADI of 55.7 (range, 41.5-72.0), suggesting they were not among the most disadvantaged nor among the most advantaged members of the population; 41% of the US population has an ADI below patients in this trial, and 28% of the US population has an ADI above patients in this trial. Although we did not systematically report on insurance status for this trial, uninsured rates in our bridge clinic have consistently been approximately 70% since inception. Studies with restrictive entrance criteria and controlled settings can bias toward including highly motivated patients and thus limit generalizability. Nonrandomized studies are difficult to interpret in this highly complex setting. The generalizability in our trial may provide important information to other researchers regarding the challenges of executing clinical initiatives among patients with OUD seen in an ACS. Challenges included implementation of an OPAT regimen, linkage of patients to continuous follow-up, and maintaining engagement via telephone with patients at just 16 weeks after discharge.

### Limitations

This study has some limitations. Although randomization should have led to an even distribution of differences, we did not systematically capture the severity of OUD or the nature of co-occurring disorders at enrollment, limiting our ability to account for the heterogeneity of treatment effect. The response rate of 26.3% to the 16-week telephone call was lower than expected. In the landmark POATS (Prescription Opioid Addiction Treatment Study) trial, the response rate at the 18-month telephone follow-up of 653 randomized participants was 38.6%, still significantly higher than in our study despite occurring at a much longer duration after follow-up.^36^ The low response rate provides important information about the study population for future researchers but may obfuscate differences in secondary outcomes. It is also possible that 16 weeks is too short a window to show improvements in patient-centered outcomes. Moreover, future studies should consider an objective assessment of follow-up outcomes compared with self-reporting in this study. A lack of information about recurrent use of acute care in settings outside our single center is a limitation that we hope to address in a subsequent study. Finally, because our trial was part of routine clinical care and was not blinded, it is possible that ACS medical professionals approached patients randomized to the bridge clinic and patients receiving usual care differently, leading to nonspecific effects favoring one condition or another.

## Conclusions

This randomized clinical trial found that among patients with OUD, referral from the general hospital to a transitional bridge clinic did not reduce hospital LOS and was associated with increases in readmission and total cost of care. Referral to a bridge clinic was associated with a higher likelihood of refilling buprenorphine prescriptions during a 16-week follow-up, increased linkage to health care professionals who provided MOUD, and fewer overdoses, but these data must be interpreted with significant caution in the setting of a high degree of missing data. These findings suggest that among a complex cohort of hospitalized patients with OUD, outpatient metrics may be positively affected through the connection to a bridge clinic, but higher resource use and higher expenditure may be required to achieve these goals. Bending the cost curve may require broader community and regional partnerships.

## Notes

Supplement 1.
Study Protocol, Statistical Analysis Plan, and Statistical Analysis Report

Supplement 2. **eAppendix.** Methods and Design

Supplement 3.
Nonauthor Collaborators

Supplement 4.
Data Sharing Statement
