# Supplementary material for: Effect of a Co-Located Bridging Recovery Initiative on Hospital Length of Stay Among Patients With Opioid Use Disorder: The BRIDGE Randomized Clinical Trial
Source: JAMA Netw Open. 2024 Feb 27;7(2):e2356430. doi: 10.1001/jamanetworkopen.2023.56430 (PMC10900965; doi:10.1001/jamanetworkopen.2023.56430)
Supplement: Supplement 1. — Study Protocol, Statistical Analysis Plan, and Statistical Analysis Report [file jamanetwopen-e2356430-s001.pdf]

Supplementary Material

Table of Contents

eSupp1 – Study Protocol ..... 2

eSupp2 – Statistical Analysis Plan ..... 11

eSupp3 – Statistical Analysis Report ..... 19

**eSupp1 – Study Protocol**

# Evaluating the Impact of the Bridge Clinic in Patients with Opioid Use Disorder


**David Marcovitz, M.D., Assistant Professor**
**Department of Psychiatry**
**Vanderbilt University Medical Center**

Sub-Investigators and their affiliations:

Patty W. Wright, M.D.
Professor, Division of Infectious Diseases, Department of Medicine, VUMC

Katie D. White, M.D., Ph.D.
Assistant Professor, Division of Infectious Diseases, Department of Medicine, VUMC

Cody Chastain, M.D.
Assistant Professor, Division of Infectious Diseases, Department of Medicine, VUMC

William Sullivan, M.Ed., M.D.
Hugh J. Morgan Chief Resident, Nashville VA Medical Center, Division of General Internal Medicine and Public Health

Heather Limper, M.P.H., Ph.D.
Research Assistant Professor of Medicine, Associate Director, Implementation Science Core, Assistant Director for Operations, CCQIR, VUMC

David Edwards, M.D., Ph.D.
Chief, Pain Medicine, Assistant Professor, Division of Pain Medicine, Department of Anesthesiology, VUMC

Christina A. Fiske, M.P.H., M.D.
Assistant Professor, Division of Infectious Diseases, Department of Medicine, VUMC

Christopher J. Lindsell, Ph.D.
Professor of Biostatistics, Associate Director, Center for Clinical Quality and Implementation Research, Director, VICTR Research Methods Program, VUMC

Todd Rice, M.Sc., M.D.
Associate Professor of Medicine, Department of Allergy/Pulmonary & Critical Care Medicine, Medical Director, Institutional Review Board, VUMC

**Table of Contents:**

**Study Schema**
1. **Study Summary**
2. **Background**
3. **Rationale and Specific Aims**
4. **Inclusion/Exclusion Criteria**
5. **Enrollment/Randomization**
6. **Study Procedures**
7. **Risks and Benefits**
8. **Reporting of Adverse Events or Unanticipated Problems involving Risk to Participants or Others**
9. **Study Withdrawal/Discontinuation**
10. **Statistical Considerations**
11. **Privacy/Confidentiality Issues**
12. **Follow-up and Record Retention**

## 1. Study Summary

**Title:** Evaluating the Impact of the Bridge Clinic in Patients with Opioid Use Disorder

**Study Aims:**

- Aim 1: To determine whether referral to the Bridge Clinic reduces overall index hospital length of stay when compared to direct referral to a long-term outpatient addiction provider for patients with active opioid use disorder (OUD) being considered for medications-for-addiction treatment (MAT).
- Aim 2: To evaluate the effects of the same intervention in the same population on secondary outcomes including costs, care-linkage, readmission rates, self-reported buprenorphine or naltrexone fills, rate of known recurrent opioid use, and measures of overall quality of life.
- Aim 3: Evaluate fidelity outcomes including Bridge Clinic acceptance and attendance rates among those referred to Bridge Clinic and reasons why patients were not considered to be eligible for inclusion.
- Aim 4: Among the subgroup of patients with an infection for which a course of IV antibiotics is required, additionally evaluate whether access to the Bridge Clinic facilitates outpatient management of the antibiotic therapy, with consequent improvement in clinical (infection and mortality) and resource (inpatient days, time to discharge following a negative blood culture) outcomes.

**Study Hypotheses:**

- Primary: Among patients with active OUD being considered for MAT, index hospital length of stay will be shorter among patients randomized to referral to the Bridge Clinic than among patients randomized to no referral to the Bridge Clinic.
- Secondary: Among patients with active OUD being considered for MAT, total costs, readmission rates, and rate of recurrent opioid use will be lower while successful care-linkage, self-reported buprenorphine or naltrexone prescription fill rates and quality of life will be higher among patients randomized to referral to the Bridge Clinic than among patients randomized to no referral to the Bridge Clinic.
- Exploratory: Among patients with active OUD being considered for MAT who also have an infection requiring IV antibiotics, referral to the Bridge Clinic will result in lower resource use without worse clinical outcomes when compared with not being referred to the Bridge clinic.

**Study Population:** Inpatients with OUD being considered for medications-for-addiction treatment (MAT) who have not previously utilized Bridge Clinic.

Due to COVID and the resulting changes in patient flow, we will temporarily suspend eligibility for the trial for OPAT patients as of December 3rd, 2020.

**Comparators:**

- Direct referral to a long-term outpatient addiction provider
- Referral to the Bridge Clinic while long-term outpatient addiction provider is located/identified

**Randomization:** Randomization will occur at the individual patient level. Currently, the Bridge Clinic can only accept a limited number of patients. To learn from the care provided, we propose randomizing all patients eligible for referral to Bridge Clinic in a ratio such that the Bridge Clinic is at capacity. In this way, the choice to refer is unbiased and the strength of evidence is greatly enhanced.

**Consent:** This study involves comparing two approaches to care: i) inpatient MAT (buprenorphine or naltrexone) with search for an outpatient MAT provider to accept the patient, or ii) inpatient MAT (buprenorphine or naltrexone) with referral to the Bridge Clinic to maintain the patient's care until a long-term outpatient MAT provider accepts the patient. Both approaches represent standard of care.

It is not practicable to answer the research question with informed consent because patients who choose to participate will necessarily differ from those who do not in substantial ways, including access to care.

We request a waiver of consent as 1) data for the research will be obtained from the medical record, financial systems, state-mandated reporting systems, and from routine standard of care telephonic outreach to patients, 2) we are comparing usual care practices, albeit with randomization, 3) there is no additional interaction with the participant for research purposes, and 4) it is impracticable to conduct the research with consent.

## 2. Background

Opioid overdoses continued to increase in Tennessee in 2017, where rates of overdose already exceed the national average, and these trends were reflected in VUH admissions for opioid use disorder (OUD)-related problems, which were up 55% in the first 6 months of 2018 over 2017.[1] Vanderbilt is implementing several initiatives to improve outcomes in patients with OUD. On July 20th, 2018, a new Addiction Consult Team (ACT) went live at Vanderbilt. The service has completed 1,082 patient visits as of May 29th 2019 (373 new visits and 709 follow up visits). Additionally, we have created an outpatient "Bridge Clinic" at VUMC that is available to manage patients with OUD for a transitional period of up to 3 months following hospital discharge. ACT clinicians staff both the consult service and the Bridge Clinic, providing continuity of care to patients leaving VUH. Staff include internal medicine, psychiatry, pain-anesthesia, nursing, social work and a recovery coach. A patient discharged from VUH who is deemed appropriate for the Bridge Clinic is written a bridge script for buprenorphine MAT. Upon discharge and between Bridge Clinic visits, the use of VUMC preferred communications (telephone, text, email) outreach by case management is implemented; telephone outreach may improve outcomes similarly to face-to-face contact among addicted patients.[2]

The Bridge Clinic was designed and implemented based on work completed at MGH, Boston Medical Center, Yale and UAB. The intent is to obviate the challenge that patients being considered for MAT are often not discharged until an outpatient provider willing to accept the patient has been identified, resulting in delays to discharge. By serving as a bridging provider, any delay in discharge is avoided. Moreover, the subset of patients requiring IV antibiotic therapy are often admitted for a full six-week course of treatment unless a skilled nursing facility is available, also at considerable expense to the healthcare system. For patients meeting low risk criteria, the multi-specialty Bridge Clinic is available to manage the antibiotic therapy as an outpatient. This provides further opportunities for the Bridge Clinic to broadly impact the hospital bed days dedicated to caring for this patient population while simultaneously providing access to dedicated care. While implemented clinically based on available evidence, the effectiveness of this care model in improving patient outcomes while reducing time in hospital has yet to be quantified in situ.

## 3. Rationale and Specific Aims

In order to rigorously evaluate the impact of the Bridge Clinic on improving care for patients with OUD, a randomized controlled trial is needed. Evidence generated in this way will support either the sustained implementation of the Bridge Clinic with scale up to meet demand, or sufficient evidence will accumulate to indicate the expected impact is not achieved and alternative approaches are needed to improve the system of care for OUD patients being considered for MAT. Given the overarching goal of evaluating the Bridge Clinic as it is operating, the specific aims of this study are to:

- <u>Aim 1:</u> To determine whether referral to the Bridge Clinic reduces overall index hospital length of stay when compared to direct referral to a long-term outpatient addiction provider for patients with active opioid use disorder (OUD) being considered for medications-for-addiction treatment (MAT).
- <u>Aim 2:</u> To evaluate the effects of the same intervention in the same population on secondary outcomes including costs, care-linkage, readmission rates, self-reported buprenorphine or naltrexone fills, rate of known recurrent opiate use, and measures of overall quality of life.
- <u>Aim 3:</u> Evaluate fidelity outcomes including Bridge Clinic acceptance and attendance rates among those referred to Bridge Clinic and reasons why patients were not considered to eligible for inclusion.
- <u>Aim 4:</u> Among the subgroup of patients with an infection for which a course of IV antibiotics is required, additionally evaluate whether access to the Bridge Clinic facilitates outpatient management of the antibiotic therapy, with consequent improvement in clinical (infection and mortality) and resource (inpatient days, time to discharge following a negative blood culture) outcomes.

To complete these aims, patients admitted to VUH with OUD and being considered for MAT, who have not previously utilized Bridge Clinic, will be enrolled in a pragmatic, single-center, randomized, controlled trial comparing referral to Bridge Clinic versus no referral to usual care in the community. Randomization ratio will be set to ensure the Bridge Clinic is functioning at capacity and the remaining patients will serve as the control. For example, if there is an average of five available slots for new referrals to the Bridge Clinic in a week, and there is an average of 20 eligible patient discharges in a week, the randomization ration will be set as 1:3.

If the Bridge Clinic is successful in bridging buprenorphine-naloxone use (as well as IM naltrexone in some cases) and linking to additional care, it is expected to decrease the overall length of stay of patients with OUD who are initiating buprenorphine-naloxone therapy. This reduction in length of stay for these patients will reduce costs and increases bed availability for other patients while simultaneously providing these patients with the care they need.

## 4. Inclusion/Exclusion Criteria

Inclusion Criteria:

- Inpatients at VUH with active OUD being considered for MAT who have not previously utilized Bridge Clinic
- Patient accepting a transitional prescription for buprenorphine-naloxone or IM naltrexone whose outpatient plans are not fixed

Exclusion Criteria:

- Deemed ineligible for referral to outpatient Bridge Clinic by the ACT (examples include by are not limited to patients with severe, active co-occurring psychiatric disorders requiring a higher level of psychiatric care or patients for whom methadone maintenance is deemed the best choice of MAT).
- Patients previously randomized in this study.
- Patients previously utilized Bridge Clinic

## 5. Enrollment/Randomization

The Addiction Consult Team (ACT) will be notified of potentially eligible patients via an EPIC consult order or referral from the general or homeless psychiatry service. Those patients for whom an order for an addiction medicine consultation is placed will be evaluated by the ACT for MAT. If the ACT considers MAT to be clinically appropriate, the patient does not have set plans for outpatient care, and the patient has not been previously randomized in this study, they will be enrolled and randomized. All other patients will be treated with usual care without availability of referral to the Bridge Clinic.

Screening information from enrolled patients will be entered into REDCap. For patients meeting objective criteria but whom the ACT determine ineligible for MAT, the reason for not enrolling them will be collected.

Once eligibility is confirmed, the patient will be randomized to being offered referral to the Bridge Clinic or not, as described below. Randomization will occur at the individual patient level. Randomization ratio will start at 1:1 and will be updated as necessary, but no more frequently than bi-weekly, to maintain the Bridge Clinic at capacity.

## 6. Study Procedures

An enrolled patient will receive a referral to social work for “outpatient MAT”. The ACT social worker will approach the patients with resources for outpatient MAT determined by randomization (though the social worker can escalate patients in either condition to a higher level of care as appropriate after randomization). All patients will be offered usual information about outpatient MAT. Patients randomized to be eligible for referral to the Bridge Clinic will additionally receive information about the Bridge Clinic and this will be offered as an option

during discussion of the plan for MAT. Patients who are randomized to not being offered a Bridge Clinic referral will not receive information about the Bridge Clinic and the Bridge Clinic will not be offered as an option.

Subsequent to comparator group assignment, patients will be followed to measure outcomes. Data will be captured from the medical record, financial systems, state-mandated reporting systems, and from other standard of care documentation. Due to complications with the social deprivation index (ADI) data within the RD, these associated variables will be obtained from the Neighborhood Atlas (https://www.neighborhoodatlas.medicine.wisc.edu/). The data elements to be collected include the following:

Baseline demographics collected at time of enrollment

- Age
- Gender
- Race
- Ethnicity
- SES indicators (e.g., ADI)

Primary Endpoint

- Overall index hospital length of stay

Additional Endpoints collected for 16 weeks following randomization

- Costs of care (total costs, and costs for each admission and care resource used (e.g. Bridge Clinic))
- Linkage to MAT provider (attending at least one visit with a MAT provider)
- Self-reported buprenorphine-naloxone (or naltrexone) prescriptions filled
- Readmission
- ED visits
- Hospital and ED free days
- Recurrent opiate use
- Overall quality of life as measured by the Schwartz Outcome Scale-10 (SOS10)
- Overdose
- Death
- Intervention Contamination (Bridge Clinic to community care or vice-versa)

Additional Endpoints for patients with infection suitable for outpatient management, collected for 16 weeks following randomization

- New, persistent, or recurrent infection (as defined by a positive culture and/or change in antibiotic regimen)
- Completion of antibiotic therapy
- Days from negative blood culture to first hospital discharge

Implementation Measurements

- Acceptance of Bridge Clinic as a bridging provider
- Reasons for ineligibility

Data will be abstracted from the EMR, REDCap and the Research Derivative (RD). Due to complications with the area deprivation index (ADI) data within the RD, these associated variables will be obtained from the Neighborhood Atlas

(https://www.neighborhoodatlas.medicine.wisc.edu/). The Research Derivative is a database of clinical and related data derived from the Medical Center's clinical systems and restructured for research. Data is repurposed from VU's enterprise data warehouse, which includes data from StarPanel, VPIMS, and ORMIS (Operating Room Management Information System), EPIC, Medipac, and HEO among others. The medical record number and other person identifiers are preserved within the database. Data types include reimbursement codes, clinical notes and documentation, nursing records, medication data, laboratory data, encounter and visit data, among others. Output may include structured data points, such as ICD 9 codes or encounter dates, semi-structured data such as laboratory tests and results, or unstructured data such as physician progress reports. The database is maintained by the Office of Research Informatics under the direction of Paul Harris, Ph.D.

## 7. **Risks and Benefits**

The risks associated with this study are limited to those associated with protection of private health information; beyond the randomization, the interaction with human subjects is limited to collection of data from existing records. Allocation made by randomization is between two usual approaches of care: i) inpatient MAT (buprenorphine or naltrexone) or appropriate alternative until a long-term outpatient MAT provider accepts the patient (sometimes after SNF stay), or ii) inpatient MAT (buprenorphine or naltrexone) or appropriate alternative with referral to the Bridge Clinic, who will maintain the patient's care until a long-term outpatient MAT provider accepts the patient. Currently, the Bridge Clinic can only accept a limited number of patients and so the choice of approach is based on availability. By randomizing the availability of the Bridge Clinic, it is possible to derive knowledge of the impact of the clinic on patient outcomes that would otherwise not be discoverable without bias. The results of this study will help understand the impact of the Bridge Clinic and be used as data for potentially expanding the resources and availability of the Bridge Clinic to additional patients or refining services to improve patient care.

We expect that patients randomized to being eligible for Bridge Clinic services will experience decreased overall length of stay. We also expect overall reduced costs. The resource savings benefit not only the study participant, but this approach will open up hospital days/beds for other patients.

Risks associated with the collection of personal health information (PHI) will be mitigated by taking all reasonable efforts to keep the information private and confidential. The minimum amount of health information necessary for study conduct will be abstracted from the medical record. PHI will be entered into REDCap, which is a secure platform for maintaining research data. Identifiers will be needed to prevent duplicate enrollments and to track patients through the health system, but user level access will be set up to limit access to any identifiers only to those study personnel who need this access. Analysis datasets may be stored on secure servers but will not include direct identifiers. Direct identifiers will not be included in datasets unless absolutely necessary.

## 8. **Reporting of Adverse Events or Unanticipated Problems Involving Risk to Participants or Others**

Participating in the study does not add risk related to treatment. The Bridge Clinic is operational and provides usual clinical care to those patients fitting within its capacity. All other processes for linking patients to outpatient MAT are well established. Events associated with MAT or other

care of patients are clinical and not associated with the research. Risks to patients from participating in the study are limited to the collection of PHI. We recognize there may be adverse events related to loss of privacy. These will be reported according to appropriate timelines. The Principal Investigator will be responsible for overseeing the study on a daily basis.

## 9. Study Withdrawal/Discontinuation

Because this study involves only observational data collection after randomization to Bridge Clinic or no Bridge Clinic availability, there are no plans to withdraw participants or discontinue them in the study. Implementation outcomes, such as uptake of Bridge Clinic referrals, are important to gather and will be included. If a patient requests that their records not be included in any research, they will not be included and all data collected for this study will be removed from the dataset. However, if analyses have already been completed and reported at the time of the request, we will retain a copy of the data in a de-identified manner to ensure rigor and reproducibility of the research.

## 10. Statistical Considerations

Statistical Analysis Plan:
Initially, we will characterize participants overall and grouped by study arm using descriptive statistics (e.g. means with standard deviations, medians with interquartile range, and counts with percentages, as appropriate). Data may also be described graphically. The primary analysis will compare length of index hospital stay between those offered referral to the Bridge Clinic and those not offered referral to the Bridge Clinic on an intent-to-treat basis. We will compare length of stay using a generalized linear model with group assignment as the primary predictor variable, with adjustment for important covariates. We expect to model the outcome as a continuous outcome. We may choose to use a proportional hazards model or a gamma GLM with a log-link function if the data are substantially skewed; we do not expect this based on our experience with length of stay in this patient cohort. For secondary outcomes, costs will be similarly modeled. Binary outcomes will be modeled using logistic regression, Quality of life outcomes will be modeled using a proportional odds regression. Additional analyses that we expect to perform include ‘per protocol’ comparisons between study groups and characterization of implementation measures.

Power and Sample Size Considerations:
We estimate 700 patients per year, or about 14 patients per week, will be eligible to participate in this study, with a capacity for approximately 3-4 new patients per week to be seen at the Bridge Clinic. We expect approximately 2/3 of patients referred to the Bridge Clinic to make their first appointment. Therefore, we will randomize patients at a rate of 1:1 to Bridge Clinic versus usual care. The randomization may be adapted if eligibility and follow up rates are different to expected. The mean length of stay for patients is currently 15 days, ranging from 3-42 and a standard deviation of about 15 days. With 700 patients in a year, allocated 1:1, we will have about 80% power to detect a 3-day reduction in length of stay. This does assume a reduced standard deviation of 10 days. If the standard deviation remains at about 15 days, the difference detectable with 80% power is 3.5 days.

Interim Analysis
Because of the disruption caused by COVID-19 and lower than expected enrollment, midway through the recruitment period we decided to re-estimate the sample size based on the

experience of patients. Blinded to allocation, we estimated the distribution of length of stay for all enrolled patients. We found the mean length of stay was shorter, at 9 days, with a standard deviation of 11. However, the distribution was decidedly right skewed. Therefore, we log transformed the length of stay variable for deciding on the final sample size. The mean length of stay was ln(5.5) days with a standard deviation of ln(2.7) days. **Assuming that a reduction in length of stay of 1.5 days is meaningful and the observed common standard deviation is a good estimate, about 168 patients per group, or 336 patients total, would be required to have 80% power to detect a difference**.

## 11. Privacy/Confidentiality Issues

At no time during the course of this study, its analysis, or its publication will patient identities be revealed. The minimum necessary data will be collected and data containing patient or provider identifiers will only be obtained as required to complete the research. Data will be unlinked from identifiers, using a unique key to facilitate linkage when needed. Data will be collected into a password-protected, secure, web-based application for managing research data (REDCap). All patients will be assigned a unique study number for use in the computerized database. At the time of publication all identifiers will be removed from the archival dataset.

## 12. Follow-up and Record Retention

The initial study is anticipated to progress from initiation to completion in about 12 months. For each participant, the study will commence at enrollment and last until 16 weeks from randomization. Patient clinical outcomes will be collected. Identified data in the secure database will be stored consistent with record retention requirements, typically 5 years from completion of the research. De-identified dataset will be maintained for secondary analyses and to support the reproducibility of reports.

# eSupp2 – Statistical Analysis Plan

# Evaluating the Impact of the Bridge Clinic in Patients with Opioid Use Disorder

**Statistical Analysis Plan**

**Version 1.0**

**April 11, 2022**

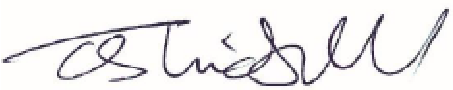

Christopher J. Lindsell, PhD

April 11, 2022
Date

## Introduction

To improve care for patients with opioid use disorder (OUD), Vanderbilt has implemented an Addiction Consult Team (ACT) and a Bridge Clinic. The primary purpose of the Bridge Clinic is to facilitate discharge for inpatients who are being considered for medications for OUD (MOUD). Specifically, the Bridge Clinic provides patients with temporary access to treatment at an outpatient clinic while they are waiting to be accepted into a long-term clinic. This document describes the statistical analysis plan for a pragmatic, single center, randomized, controlled trial designed to evaluate the impact of the Bridge Clinic on improving care for patients with active OUD. This document has been prepared prior to final data collection and unblinding. Because inpatient providers may delay discharge for patients with OUD who lack a source of outpatient care for MOUD, it is hypothesized that OUD patients referred to the Bridge Clinic will have a decreased overall length of stay, will follow up with their Bridge Clinic provider, and will have a reduction in overall costs for care.

## Population and design considerations

*Study Population:*

Inpatients at VUH with OUD being considered for MOUD who have not used the Bridge Clinic before and who do not have a fixed outpatient plan are considered potentially eligible for this trial. If the ACT considers MOUD is clinically appropriate for the patient, the patient does not have plans for outpatient care, and the patient has not been enrolled in this study previously, they are enrolled, randomized, and offered a referral to either Bridge Clinic or treatment as usual (TAU) depending on randomization.

*Study Design:*

This study is designed as a pragmatic, single center, randomized, controlled trial comparing clinical outcomes between patients assigned to receive a referral to the Bridge Clinic and patients assigned to TAU.

*Randomization:*

Randomization occurs at the individual patient level. The Bridge Clinic can only accept a limited number of patients, so eligible patients will be randomized in a ratio so that the Bridge Clinic is at capacity. To start, the ratio will be 1:1, and can be updated as often as bi-weekly to maintain the Bridge Clinic capacity. There has been no indication or need to change the ratio during the study.

*Sample Size Considerations:*

We estimated about 700 patients per year would be eligible for this study, with a capacity for about 3-4 new patients a week to be seen at the Bridge Clinic. We expected about 2/3 of patients referred to the Bridge Clinic to make their first appointment. The mean length of stay was 15 days with a standard deviation of about 15 days. Assuming a reduced standard deviation of 10 days, with 700 patients a year and a 1:1 randomization ratio, we would have 80% power to detect a 3-day reduction in length of stay. If the standard deviation remains at 15 days, we would have 80% power to detect a 3.5-day reduction in length of stay. Due to COVID-19, enrollment was lower than expected and hospital inpatient experiences differed from historical data. Midway through the recruitment period, the overall, ungrouped distribution of length of stay was re-estimated based on participant experience. Based on the revised assumptions, to detect a 1.5-day

reduction in length of stay and assuming the observed common standard deviation is a good estimate, about 336 patients (168 per group) is needed to have 80% power.

**Interventions**
Inpatients with OUD who are deemed eligible for this trial are enrolled and randomized. The patients are randomized to one of two treatment arms:

a) Direct referral to a long-term outpatient addiction provider (TAU)
b) Referral to Bridge Clinic for temporary care while a long-term outpatient addiction provider is identified (intervention)

**Endpoints**
*Primary Endpoint*
The primary endpoint is the overall index hospital length of stay. The index length of stay is defined as the time between the admission and time of discharge during the inpatient visit when the participant is identified as being eligible for the study.

*Secondary Endpoints*
Multiple secondary endpoints are prespecified for this trial. Secondary endpoints are collected for 16 weeks following randomization:

a) Overall quality of life as measured by the Schwartz Outcome Scale-10 (SOS10). The SOS10 has ten questions scored on a 0 (never) to 6 (all or nearly all of the time) scale. A total score is computed as the sum across the 10 questions. If two questions are missing, a mean score can be imputed for the missing values to generate a total score. No further imputation will be used for this score. This is self-reported and captured at the 16-week follow-up call.
b) Linkage to MOUD provider, defined as attending at least one visit with a MOUD provider after discharge, assessed by self-report at 16-week follow-up call.
c) Self-reported buprenorphine-naloxone (or naltrexone) prescriptions filled (number filled) at the 16-week follow-up call.
d) Any recurrent opioid use reported at the 16-week phone call follow-up.
e) Number of recurrent opioid uses reported at the 16-week phone call follow-up.
f) Number of ED visits or readmissions to VUH 16 weeks post discharge identified in the electronic medical record.
g) Hospital and emergency department free days
    a. Hospital free days will be calculated as a count of whole days during the 16 weeks the subject is not in the hospital. If the subject dies before the end of 16 weeks, hospital free days will be equal to -1.
    b. Hospital and emergency department free days will be calculated as a count of whole days during the 16 weeks the subject is not in the emergency department or in the hospital. If the subject dies before the end of 16 weeks, emergency free days will be equal to -1.
h) Any overdose reported at the 16-week phone call follow-up.
i) Death in hospital or documented at the 16 week follow up or in the medical record.
j) Costs of care, defined as total costs for all care episodes at VUMC 16 weeks post discharge.

*Exploratory Endpoints*
Exploratory endpoints have been specified for patients with infection suitable for outpatient antibiotic therapy (OPAT) management. These endpoints are collected for 16 weeks following randomization by review of VUMC's electronic medical record:

a) New, persistent, or recurrent infection: defined by positive culture and/or change in antibiotic regimen
b) Completion of antibiotic therapy
c) Days from negative blood culture (i.e., blood draw date) to first hospital discharge

*Implementation Endpoints*

a) Acceptance of Bridge Clinic as a bridging provider, defined by being physically checked in for at least 1 visit with the Bridge Clinic within 16 weeks of follow up.
b) Reasons for ineligibility among participants screened, specifically:
    - Absence of a qualifying OUD
    - Previously linked with a MOUD provider either prior to or during the current visit?
    - ACT determines the participant does not qualify

*Fidelity Endpoint*

a) Cross-over, defined as when a patient randomized to usual care is offered the Bridge Clinic, or when a patient is randomized to Bridge Clinic but is only offered usual care, at the time of discharge from the inpatient stay.

**Analysis dataset**
The analysis for the trial will use an intent-to-treat approach to answer the effectiveness question posed. That is, participants will be evaluated by treatment group as assigned regardless of what was delivered. All eligible participants will be included. There is no plan to restrict the analysis to a per protocol set.

**Statistical Approach**
Our initial analysis will be descriptive in nature, summarizing information that characterizes the cohort and the outcomes. Then, we will proceed with inferential analysis to answer the main study question. Then, we will compare the secondary endpoints between study groups.

*Descriptive Analysis*
To characterize the study sample, baseline demographic and clinical data will be described overall and by group. Categorical variables will be described using frequencies and proportions, and continuous variables will be described using means and standard deviations, as well as medians and interquartile ranges. Missingness will be reported for each variable. Graphical summaries using box plots, violin plots, and/or histograms may be used to describe the data graphically. At a minimum, the following variables will be described at time of enrollment:

- Age (years)
- Gender (male, female, unknown)
- Race (African American, Asian/Pacific Islander, Caucasian, Multiple, Native American, Other, Unknown)
- Ethnicity (Hispanic, Non-Hispanic, Unknown)
- Census tract area deprivation index

We will describe all of the outcome variables overall and grouped by study arm using the same approach as for the demographic data. Summary statistics and graphical representations may be displayed, and missingness will be reported for each variable.

No statistical comparisons between groups will be done for this descriptive analysis.

*Main Analysis*
The primary outcome variable (hospital index length of stay) will be compared between groups using an adjusted generalized linear model. Since the data are positive and may be skewed, like a gamma distribution, we may use a negative inverse link function, a proportional odds model, or transform the data.

In this analysis, group assignment is the main predictor variable. For drawing conclusions about the effect of the Bridge clinic, a critical p-value of 0.05 for the effect of treatment group assignment on the primary outcome will be used. The model will be adjusted for age, race, ethnicity, and area deprivation index. The influence of continuous variables will be assessed using restricted cubic splines with a minimum of three knots. We do not expect missingness in our primary outcome. If there are missing covariates, cases will not be excluded; we will use multiple imputation with predictive mean matching for missingness in adjusting covariates.

Secondary and implementation outcomes will be compared between study groups using adjusted generalized linear models, just as for the primary endpoint. Binary endpoints will use a logit link function. For counts, such as number of medication refills, either a zero-inflated Poisson or a negative binomial model will be fit.

Exploratory outcomes for OPAT patients will not be compared between study groups but will be reported by study group.

There may be missingness in secondary or implementation outcomes. If there are missing outcomes, these may be imputed of they occur in less than 5% of cases. Otherwise, the cohort for which the outcome is available will be described, along with the results of the model evaluating treatment effects in this cohort. All model results will be summarized with point estimates and 95% confidence intervals (CIs), which will be emphasized over p-values when reporting the results for secondary and implementation outcomes. No adjustments for multiplicity will be made.

*Differential treatment effects*
To determine whether effects of treatment on the primary endpoint depends on any of the baseline characteristics, we will test the interaction between the baseline characteristics and treatment effect in the regression model. If evidence of an interaction is observed using a threshold of $p<0.2$, we will proceed to subgroup analyses. For categorical variables, endpoints will be compared between within categories. For continuous variables, we will display the partial effects plots showing how treatment effects change with the putative subgrouping variable.

**Summary**

The results of this study will help to determine whether the Bridge Clinic is effective at improving patient outcomes. The analysis approach we describe is selected based on the trial's pragmatic nature and the intent to understand the effectiveness of Bridge Clinic when compared to TAU.

# Evaluating the Impact of the Bridge Clinic in Patients with Opioid Use Disorder

**Statistical Analysis Plan Addendum**

**March 1, 2023**

**Endpoints**

*Exploratory Endpoints*

Post hoc, it was determined that there is uncertainty in specifying the timing of a consult with the inpatient psychiatric service. Therefore, an additional exploratory length of stay endpoint was defined using a consistent start point. This hospital length of stay variable is defined as the time between the time of randomization and the time of discharge during the index inpatient visit. This endpoint is incorporated into the statistical plan prior as an exploratory endpoint.

Christopher J. Lindsell — 3/1/2023

Christopher J. Lindsell, PhD — Date

**eSupp3 – Statistical Analysis Report**

# Evaluating the Impact of the Bridge Clinic in Patients with Opioid Use Disorder

Cassie Hennessy

2023-06-20

## Table of Contents

Project Summary ..... 22
Data Summary ..... 23
Table 1: Demographics by treatment group and overall ..... 23
Figure 1: Continuous Variable Distributions ..... 24
Outcomes ..... 25
Table 2: Primary outcome: overall index hospital length of stay ..... 25
Figure 2: Index hospital length of stay, overall and by group ..... 25
Table 3: Secondary outcomes ..... 26
Figure 3a: Number of Readmissions ..... 29
Figure 3b: Number of ED Visits ..... 29
Figure 3c: Number of Readmissions or ED Visits ..... 29
Figure 4: Cost of care ..... 30
Figure 5: Quality of life ..... 30
Table 4: Exploratory Outcomes ..... 31
Table 5a: Implementation Outcome - Acceptance of Bridge Clinic ..... 32
Table 5b: Implementation Outcome - Reasons for ineligibility ..... 32
Table 6: Fidelity Outcome ..... 33
Primary Analysis ..... 34
Unadjusted Primary Analysis ..... 34
Adjusted Primary Analysis ..... 34
Test differential treatment effects ..... 36
Secondary Analysis ..... 38
Number of readmissions ..... 39
Unadjusted - Proportional odds model ..... 39
Adjusted - Proportional odds model ..... 39

Number of ED visits ..... 41
Unadjusted - Proportional odds model ..... 41
Adjusted - Proportional odds model ..... 41
Number of readmissions or ED visits ..... 43
Unadjusted - Proportional odds model ..... 43
Adjusted - Proportional Odds Model ..... 43
Recurrent opioid use ..... 45
Unadjusted ..... 45
Adjusted ..... 45
Opioid use in last 30 days ..... 47
Unadjusted ..... 47
Adjusted ..... 47
Total cost of care ..... 48
Unadjusted ..... 48
Adjusted ..... 49
Linkage to MOUD provider ..... 50
Unadjusted ..... 50
Adjusted ..... 51
Quality of life ..... 53
Unadjusted ..... 53
Adjusted ..... 53
Overdose ..... 55
Unadjusted ..... 55
Adjusted ..... 55
Death ..... 56
Unadjusted ..... 56
Adjusted ..... 57
Hospital free days ..... 58
Unadjusted ..... 58
Adjusted ..... 59
Hospital and ED free days ..... 60
Unadjusted ..... 60
Adjusted ..... 61

Number of buprenorphine refills ..... 63
Unadjusted ..... 63
Adjusted ..... 63
Implementation Outcome Analysis ..... 65
Acceptance of Bridge Clinic as a bridging provider ..... 65
Unadjusted ..... 65
Adjusted ..... 65
Post Hoc Analyses ..... 67
Binary logistic regression for readmissions, ED visits, and readmission or ED visit ..... 67
Readmission: yes/no within 16 week follow up ..... 67
ED visits: yes/no within 16 week follow up ..... 68
Readmission or ED visit: yes/no within 16 week follow up ..... 70
Exploratory outcome: Index hospital length of stay from time of randomization..... 72
Table 7: LOS from time of randomization ..... 72
Figure 6: Index hospital length of stay from randomization, overall and by group ..... 72
Analysis ..... 72
Propensity Score Analysis ..... 75
Linkage to MOUD Provider ..... 76
Recurrent opioid use ..... 78
Opioid use in last 30 days ..... 80
Overdose ..... 82
Number of buprenorphine refills ..... 84
Quality of Life ..... 86

## Project Summary

A pragmatic, single center, randomized, controlled trial designed to evaluate the impact of the Bridge Clinic on improving care for patients with active opioid use disorder (OUD).

- The Bridge Clinic provides patients with temporary access to treatment at an outpatient clinic while they are waiting to be accepted into a long-term clinic

**Hypothesis:** Because inpatient providers may delay discharge for patients with OUD who lack a source of outpatient care for MOUD, it is hypothesized that OUD patients referred to the Bridge Clinic will have a decreased overall length of stay, will follow up with their Bridge Clinic provider, and will have a reduction in overall costs for care.

**Inclusion:**

- Adults (age >= 18 years old)
- Inpatients at VUH
- Active OUD
- Being considered for MAT (MOUD)
- No fixed outpatient plan
- ACT considers MOUD clinically appropriate for the patient

**Exclusion:**

- Ineligible for referral to outpatient Bridge Clinic by ACT
- Previously randomized in the study
- Previously used Bridge Clinic

**Interventions:**

- Direct referral to a long-term outpatient addiction provider (Usual Care)
- Referral to Bridge Clinic to temporary care while a long-term outpatient addiction provider is identified

**Calculate quartiles for ADI, using National Percentile (0-100)**

- Quartile 1: [0,42)
- Quartile 2: [42,55)
- Quartile 3: [55,72)
- Quartile 4: [72,100]

40 subjects are allocated to specific quartiles, as determined by the study team

- 38 subjects allocated to Quartile 1
- 2 subjects allocated to Quartile 2

# Data Summary

## Table 1: Demographics by treatment group and overall

| | Overall | Bridge Clinic | Usual Care |
|---|---|---|---|
| N | 335 | 167 | 168 |
| Age (mean (SD)) | 39.8 (10.3) | 40.4 (10.8) | 39.1 (9.7) |
| Age (median [IQR]) | 38.0 [31.9, 45.7] | 38.7 [32.1, 47.0] | 37.9 [31.8, 44.5] |
| Sex | | | |
| Female | 141 (42.1) | 72 (43.1) | 69 (41.1) |
| Male | 194 (57.9) | 95 (56.9) | 99 (58.9) |
| Area Deprivation Index (Quartiles) | | | |
| Quartile 1 | 112 (33.4) | 55 (32.9) | 57 (33.9) |
| Quartile 2 | 73 (21.8) | 35 (21.0) | 38 (22.6) |
| Quartile 3 | 71 (21.2) | 35 (21.0) | 36 (21.4) |
| Quartile 4 | 79 (23.6) | 42 (25.1) | 37 (22.0) |
| Race | | | |
| White | 287 (85.7) | 144 (86.2) | 143 (85.1) |
| Black or African American | 36 (10.7) | 16 ( 9.6) | 20 (11.9) |
| Other | 10 ( 3.0) | 6 ( 3.6) | 4 ( 2.4) |
| Missing/Unknown | 2 ( 0.6) | 1 ( 0.6) | 1 ( 0.6) |
| Ethnicity | | | |
| Hispanic/Latino | 12 ( 3.6) | 6 ( 3.6) | 6 ( 3.6) |
| Non-Hispanic/Latino | 320 (95.5) | 161 (96.4) | 159 (94.6) |
| Missing/Unknown | 3 ( 0.9) | 0 ( 0.0) | 3 ( 1.8) |

## Figure 1: Continuous Variable Distributions

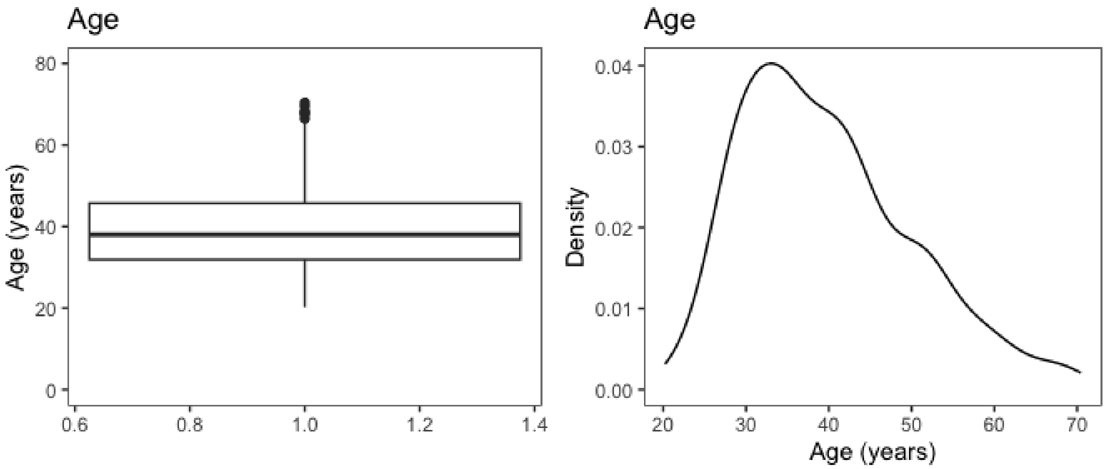

# Outcomes

## Table 2: Primary outcome: overall index hospital length of stay

- The time between admission and time of discharge during the inpatient visit when the participant is identified as being eligible for the study

| | Overall | Bridge Clinic | Usual Care |
|---|---|---|---|
| n | 335 | 167 | 168 |
| Length of stay (hours) (mean (SD)) | 223.6 (252.0) | 215.0 (236.1) | 232.1 (267.4) |
| Length of stay (hours) (median [IQR]) | 139.1 [84.6, 258.0] | 141.7 [78.1, 276.1] | 136.2 [86.9, 256.7] |

## Figure 2: Index hospital length of stay, overall and by group

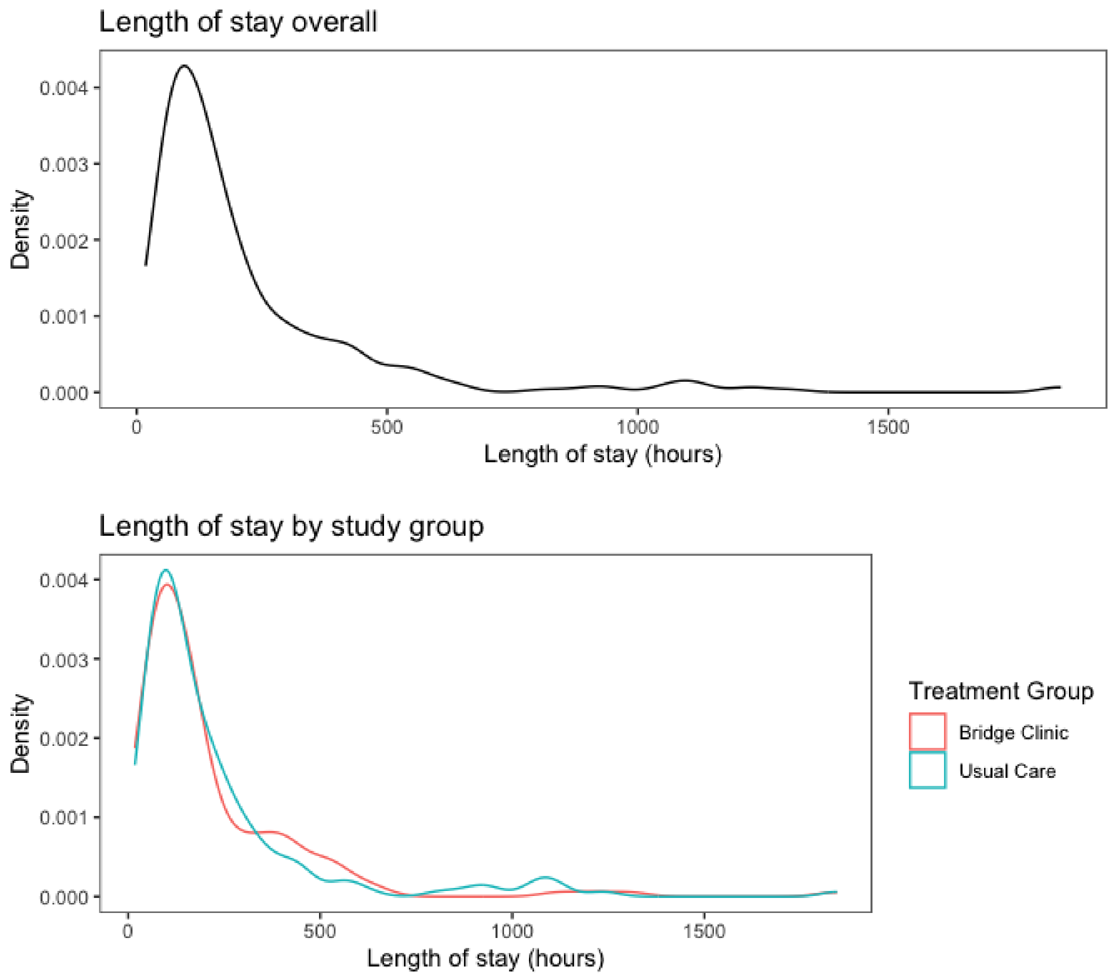

## Table 3: Secondary outcomes

- Readmission: number of readmissions to VUH 16 weeks post discharge
- ED Visits: number of ED visits to VUH 16 weeks post discharge
- Readmission and ED visits: number of ED visits or readmissions to VUH 16 weeks post discharge
- Recurrent opioid use: yes/no; any recurrent opioid use within the 16 week follow up
- Opioid use in the last 30 days:
  - No use, single use, multiple use
- Cost of care: total costs for all care episodes at VUMC 16 weeks post discharge
  - Remove the patient encounters listed as 'NI'
  - Sum up the Total Costs for each patient, across their encounters
- Linkage to MOUD provider: yes/no; attended at least one visit with a MOUD provider after discharge
- Quality of Life: Schwartz Outcome Scale-10
  - Sum across the 10 questions
  - If one or two questions are missing, the mean of the non-missing questions is used to impute the missing column
  - If three or more questions are missing, the quality of life is missing
- Overdose: yes/no; within the 16 week follow up
- Death: yes/no; within the 16 week follow up
- Hospital free days and Hospital free & ED free days:
  - Hospital free days will be calculated as a count of whole days during the 16 weeks the subject is not in the hospital. If the subject dies before the end of 16 weeks, hospital free days will be equal to -1.
  - Hospital and emergency department free days will be calculated as a count of whole days during the 16 weeks the subject is not in the emergency department or in the hospital. If the subject dies before the end of 16 weeks, emergency free days will be equal to -1.
  - Per SAP, admission and discharge date each count as 1 day in the hospital/ED so the calculated LOS should not be used for this
  - Some patients have inpatient encounters listed that happened during their index hospital stay (i.e., were in the hospital for multiple days and had a surgery or lab after they were enrolled in the study). These should be filtered out to only include subsequent encounters 16 weeks after discharge from index stay.
  - If a subject has no hospital or emergency room encounters and does not die within the 16 week follow up, hospital and hospital & ED frees are set to 112 days (16 weeks)
- Self-reported buprenorphine-naloxone (or naltrexone) prescriptions filled (number filled) at the 16-week follow-up call

| | Overall | Bridge Clinic | Usual Care |
|---|---|---|---|

| | Overall | Bridge Clinic | Usual Care |
|---|---|---|---|
| N | 335 | 167 | 168 |
| Number of readmissions (median [IQR]) | 0.00 [0.00, 0.00] | 0.00 [0.00, 1.00] | 0.00 [0.00, 0.00] |
| At least one readmission (%) | | | |
| Yes | 70 (20.9) | 45 (26.9) | 25 (14.9) |
| No | 265 (79.1) | 122 (73.1) | 143 (85.1) |
| Number of ED visits (median [IQR]) | 0.00 [0.00, 0.00] | 0.00 [0.00, 0.00] | 0.00 [0.00, 0.00] |
| At least one ED visit (%) | | | |
| Yes | 76 (22.7) | 40 (24.0) | 36 (21.4) |
| No | 259 (77.3) | 127 (76.0) | 132 (78.6) |
| Number of readmissions or ED visits (median [IQR]) | 0.00 [0.00, 1.00] | 0.00 [0.00, 1.00] | 0.00 [0.00, 1.00] |
| At least one readmission or ED visit (%) | | | |
| Yes | 121 (36.1) | 69 (41.3) | 52 (31.0) |
| No | 214 (63.9) | 98 (58.7) | 116 (69.0) |
| Recurrent opioid use (%) | | | |
| Yes | 38 (11.3) | 23 (13.8) | 15 ( 8.9) |
| No | 50 (14.9) | 33 (19.8) | 17 (10.1) |
| Missing | 247 (73.7) | 111 (66.5) | 136 (81.0) |
| Opioid use in last 30 days (%) | | | |
| No use | 4 ( 1.2) | 2 ( 1.2) | 2 ( 1.2) |
| Single use | 4 ( 1.2) | 0 ( 0.0) | 4 ( 2.4) |
| Multiple use | 24 ( 7.2) | 17 (10.2) | 7 ( 4.2) |
| Missing | 303 (90.4) | 148 (88.6) | 155 (92.3) |
| Total cost of care (mean (SD)) | 20052.86 (34970.10) | 25066.64 (40311.33) | 14740.26 (27379.09) |
| Total cost of care (median [IQR]) | 4926.93 [678.99, 22109.90] | 9481.93 [1478.28, 29376.14] | 1705.28 [360.16, 12146.33] |
| Missing | 24 ( 7.2) | 7 ( 4.2) | 17 (10.1) |

| | Overall | Bridge Clinic | Usual Care |
|---|---|---|---|
| Linkage to MOUD provider (%) | | | |
| Yes | 64 (19.1) | 45 (26.9) | 19 (11.3) |
| No | 24 ( 7.2) | 11 ( 6.6) | 13 ( 7.7) |
| Missing | 247 (73.7) | 111 (66.5) | 136 (81.0) |
| Quality of Life (mean (SD)) | 40.42 (16.44) | 40.36 (17.75) | 40.53 (14.21) |
| Quality of Life (median [IQR]) | 44.00 [29.50, 54.00] | 47.00 [26.00, 53.75] | 41.50 [32.50, 54.25] |
| Missing | 249 (74.3) | 113 (67.7) | 136 (81.0) |
| Overdose (%) | | | |
| Yes | 5 ( 1.5) | 1 ( 0.6) | 4 ( 2.4) |
| No | 81 (24.2) | 53 (31.7) | 28 (16.7) |
| Missing | 249 (74.3) | 113 (67.7) | 136 (81.0) |
| Death (%) | | | |
| Yes | 6 ( 1.8) | 2 ( 1.2) | 4 ( 2.4) |
| No | 329 (98.2) | 165 (98.8) | 164 (97.6) |
| Hospital free days (mean (SD)) | 107.09 (16.79) | 106.38 (15.70) | 107.80 (17.83) |
| Hospital free days (median [IQR]) | 112.00 [112.00, 112.00] | 112.00 [109.00, 112.00] | 112.00 [112.00, 112.00] |
| Hospital and ED free days (mean (SD)) | 106.54 (16.88) | 105.77 (15.85) | 107.31 (17.86) |
| Hospital and ED free days (median [IQR]) | 112.00 [110.00, 112.00] | 112.00 [107.00, 112.00] | 112.00 [111.00, 112.00] |
| Self-reported number of buprenorphine fills (mean (SD)) | 7.60 (7.57) | 10.26 (7.80) | 3.65 (5.17) |
| Self-reported number of buprenorphine fills (median [IQR]) | 6.00 [0.00, 15.00] | 10.00 [4.00, 16.00] | 1.00 [0.00, 6.00] |
| Missing | 258 (77.0) | 121 (72.5) | 137 (81.5) |

## Figure 3a: Number of Readmissions

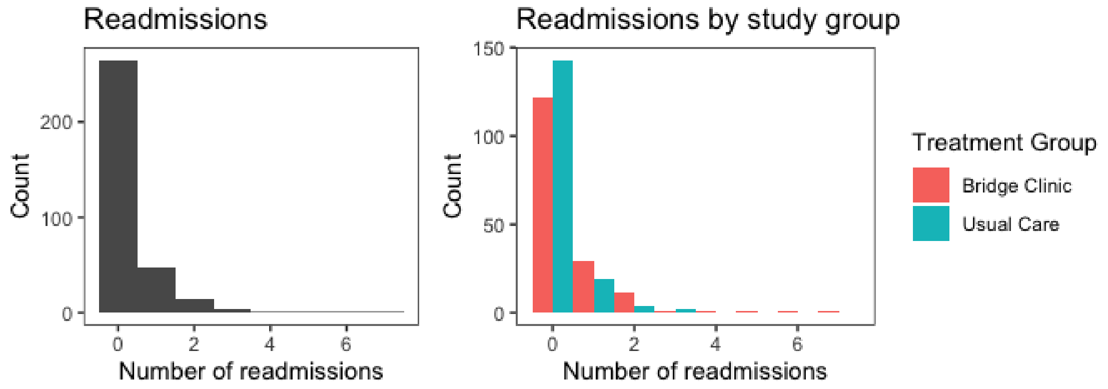


## Figure 3b: Number of ED Visits

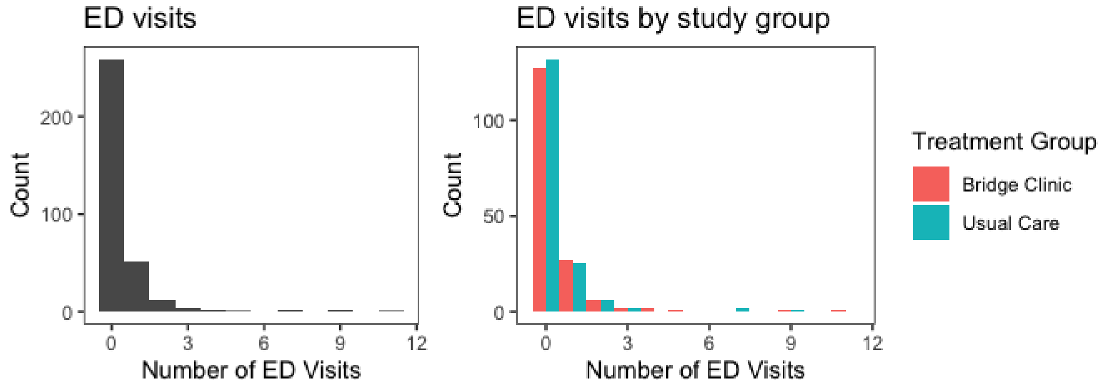


## Figure 3c: Number of Readmissions or ED Visits

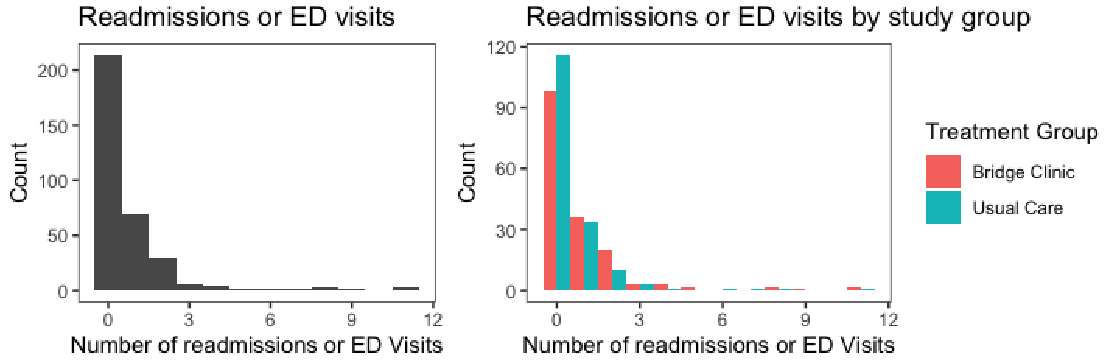

## Figure 4: Cost of care

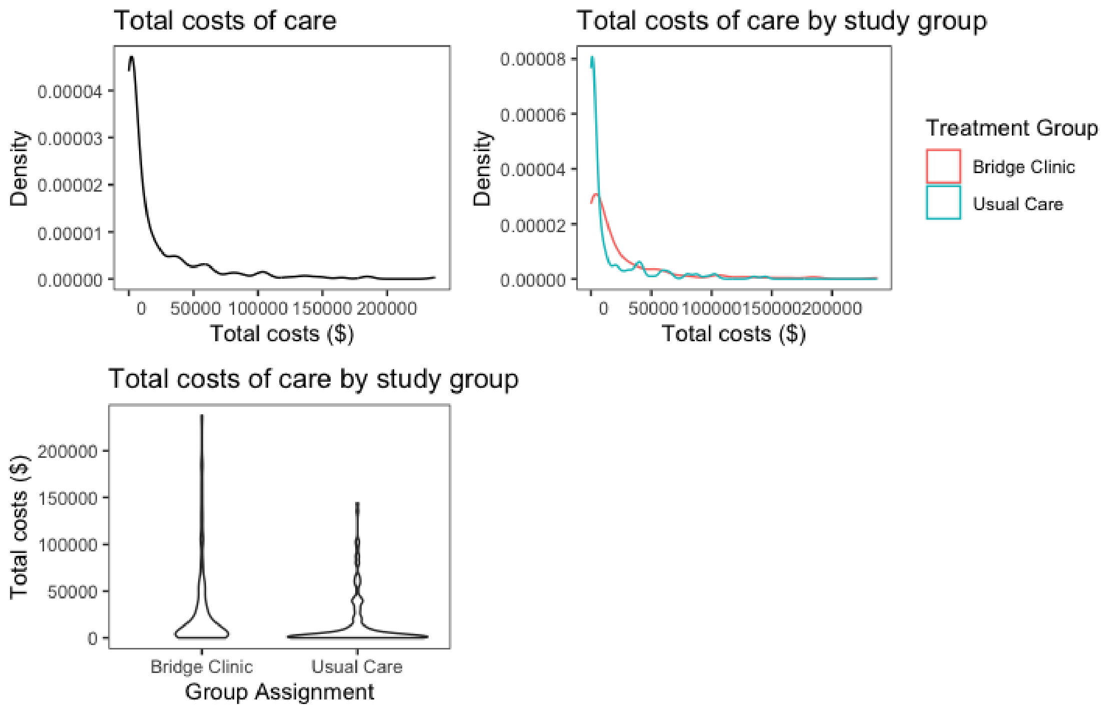


## Figure 5: Quality of life

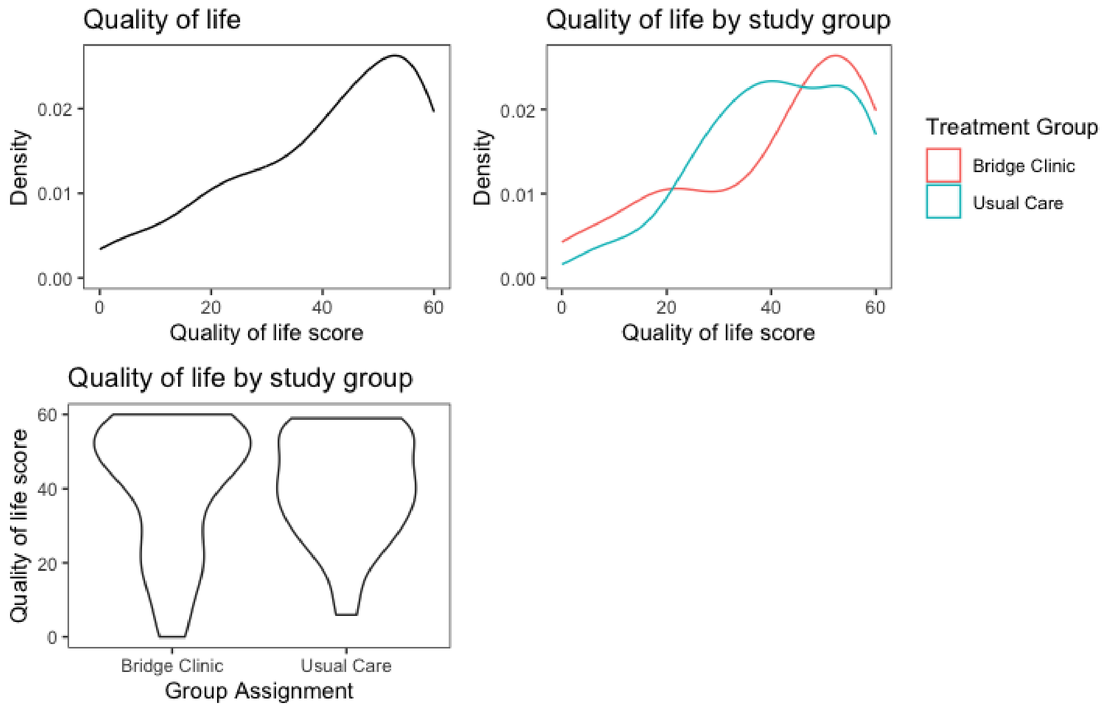

## Table 4: Exploratory Outcomes

**Exploratory outcomes for OPAT patients will not be compared between study groups but will be reported by study group**

Exploratory endpoints have been specified for patients with infection suitable for outpatient antibiotic therapy (OPAT) management

- New, persistent, or recurrent infection: defined by positive culture and/or change in antibiotic regimen
- Completion of antibiotic therapy
- Days from negative blood culture (i.e., blood draw date) to first hospital discharge

| | Overall | Bridge Clinic | Usual Care |
|---|---|---|---|
| N | 7 | 6 | 1 |
| New, persistent, or recurrent infection (%) | | | |
| No | 7 (100.0) | 6 (100.0) | 1 (100.0) |
| Yes | 0 ( 0.0) | 0 ( 0.0) | 0 ( 0.0) |
| Completion of antibiotic therapy (%) | | | |
| No | 1 ( 14.3) | 0 ( 0.0) | 1 (100.0) |
| Yes | 6 ( 85.7) | 6 (100.0) | 0 ( 0.0) |
| Days from negative blood culture (mean (SD)) | 17.40 (8.96) | 19.75 (8.38) | 8.00 (NA) |
| Days from negative blood culture (median [IQR]) | 15.00 [12.00, 21.00] | 18.00 [14.25, 23.50] | 8.00 [8.00, 8.00] |
| Missing | 2 (28.6) | 2 (33.3) | 0 ( 0.0) |

## Table 5a: Implementation Outcome - Acceptance of Bridge Clinic

- Acceptance of Bridge Clinic as a bridging provider, defined by being physically checked in for at least 1 visit with the Bridge Clinic within 16 weeks of follow up.

| | | Overall | Bridge Clinic | Usual Care |
|---|---|---|---|---|
| n | | 335 | 167 | 168 |
| At least one Bridge Clinic Visit (%) | No | 237 (70.7) | 70 (41.9) | 167 (99.4) |
| | Yes | 98 (29.3) | 97 (58.1) | 1 ( 0.6) |

## Table 5b: Implementation Outcome - Reasons for ineligibility

- Reasons for ineligibility among participants screened, specifically:
  - Absence of a qualifying OUD
  - ACT determines the participant does not qualify

| | Overall |
|---|---|
| n (number screened but not enrolled) | 37 |
| Does the patient have OUD? = No (%) | 1 (11.1) |
| Does the ACT believe the patient is a candidate for Bridge Clinic = No (%) | 3 (60.0) |

## Table 6: Fidelity Outcome

- Intervention Contamination: defined as when a patient randomized to usual care is offered the Bridge Clinic, or when a patient is randomized to Bridge Clinic but is only offered usual care, at the time of discharge from the inpatient stay.

| | Overall | Bridge Clinic | Usual Care |
|---|---|---|---|
| n | 335 | 167 | 168 |
| Intervention Contamination = Yes (%) | 1 (1.2) | 1 (1.9) | 0 (0.0) |

## Primary Analysis

- Since the data are positive and skewed, a proportional odds model is used

### Unadjusted Primary Analysis

- Outcome: index hospital length of stay (hours)
- Predictor: Group assignment (Bridge Clinic or usual care)
- Number of observations included in this analysis: 335

| | Low | High | Diff. | Effect | S.E. | Lower 0.95 | Upper 0.95 | Type |
|---|---|---|---|---|---|---|---|---|
| Group Assignment (Bridge Clinic vs Usual Care) | 2 | 1 | | -0.06 | 0.19 | -0.43 | 0.31 | 1 |
| Odds Ratio | 2 | 1 | | 0.94 | | 0.65 | 1.37 | 2 |

The unadjusted OR (95% CI) for the index hospital length of stay being longer for a subject in Bridge Clinic vs Usual Care is 0.944 (0.651-1.368), p = 0.761.

### Adjusted Primary Analysis

**Multiple Imputation**

- Multiple imputation was used to impute missing data for race and ethnicity

**Proportional Odds Model**

- Outcome: index hospital length of stay (hours)
- Predictor: Group assignment (Bridge Clinic or usual care)
- Adjusted for age, race, ethnicity, area deprivation index (quartiles)
  - Restricted cubic splines are used for continuous covariates (with 3 knots)
    - Age
- Number of observations included in this analysis: 335

| | Low | High | Diff. | Effect | S.E. | Lower 0.95 | Upper 0.95 | Type |
|---|---|---|---|---|---|---|---|---|
| Age | 31.93 | 45.72 | 13.79 | 0.26 | 0.16 | -0.05 | 0.58 | 1 |
| Odds Ratio | 31.93 | 45.72 | 13.79 | 1.30 | | 0.95 | 1.79 | 2 |
| Group Assignment (Bridge Clinic vs Usual Care) | 2.00 | 1.00 | | -0.06 | 0.19 | -0.44 | 0.31 | 1 |
| Odds Ratio | 2.00 | 1.00 | | 0.94 | | 0.65 | 1.36 | 2 |
| Race (Black or African American vs White) | 1.00 | 2.00 | | -0.13 | 0.31 | -0.75 | 0.48 | 1 |
| Odds Ratio | 1.00 | 2.00 | | 0.87 | | 0.47 | 1.62 | 2 |
| Race (Other vs White) | 1.00 | 3.00 | | -0.45 | 0.72 | -1.85 | 0.95 | 1 |
| Odds Ratio | 1.00 | 3.00 | | 0.64 | | 0.16 | 2.60 | 2 |
| Ethnicity (Hispanic/Latino vs Non-Hispanic/Latino) | 2.00 | 1.00 | | 0.75 | 0.66 | -0.55 | 2.05 | 1 |
| Odds Ratio | 2.00 | 1.00 | | 2.12 | | 0.58 | 7.76 | 2 |
| ADI Quartile (2 vs 1) | 1.00 | 2.00 | | 0.15 | 0.26 | -0.37 | 0.66 | 1 |
| Odds Ratio | 1.00 | 2.00 | | 1.16 | | 0.69 | 1.94 | 2 |
| ADI Quartile (3 vs 1) | 1.00 | 3.00 | | 0.12 | 0.26 | -0.40 | 0.63 | 1 |
| Odds Ratio | 1.00 | 3.00 | | 1.12 | | 0.67 | 1.88 | 2 |
| ADI Quartile (4 vs 1) | 1.00 | 4.00 | | 0.08 | 0.26 | -0.42 | 0.59 | 1 |
| Odds Ratio | 1.00 | 4.00 | | 1.09 | | 0.66 | 1.80 | 2 |

The adjusted OR (95% CI) for the index hospital length of stay being longer for a subject in Bridge Clinic vs Usual Care is 0.939 (0.646-1.364), p = 0.74.

## Test differential treatment effects

Differential treatment effects will be evaluated at a significance level = .2, decided a priori.

- Age
- Race
- Ethnicity
- Area deprivation index (quartiles)

### Age

```
##
## Model 1: los_hrs ~ Group.Assignment. * rcs(AGE, 3) + RACE_2 + ETHNI
CITY +
##     quartile_grp
## Model 2: los_hrs ~ Group.Assignment. + rcs(AGE, 3) + RACE_2 + ETHNI
CITY +
##     quartile_grp
##
## L.R. Chisq       d.f.          P
##  1.5642566  2.0000000  0.4574314
```

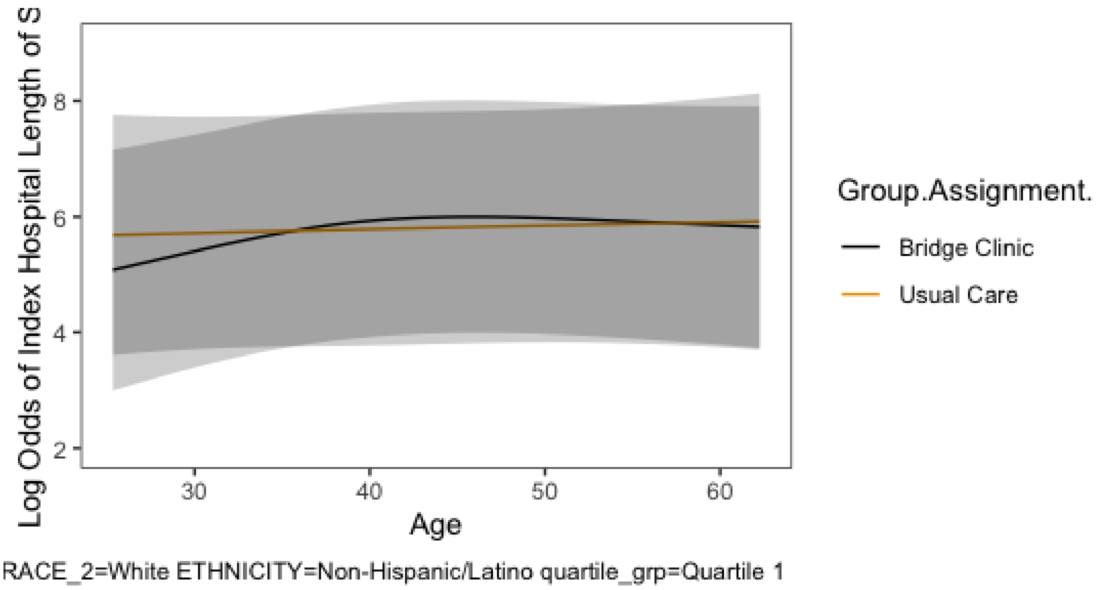


There is no evidence suggesting that the effect of intervention group on the index hospital length of stay depends on age (p-value = 0.457).

### Race

```
##
## Model 1: los_hrs ~ Group.Assignment. * RACE_2 + rcs(AGE, 3) + ETHNI
CITY +
##     quartile_grp
## Model 2: los_hrs ~ Group.Assignment. + rcs(AGE, 3) + RACE_2 + ETHNI
CITY +
##     quartile_grp
##
## L.R. Chisq       d.f.          P
##   3.055476   2.000000   0.217026
```

There is no evidence suggesting that the effect of intervention group on the index hospital length of stay depends on race (p-value = 0.217).

**Ethnicity**

```
##
## Model 1: los_hrs ~ Group.Assignment. * ETHNICITY + rcs(AGE, 3) + RA
CE_2 +
##     quartile_grp
## Model 2: los_hrs ~ Group.Assignment. + rcs(AGE, 3) + RACE_2 + ETHNI
CITY +
##     quartile_grp
##
## L.R. Chisq       d.f.          P
##  0.1975030  1.0000000  0.6567439
```

There is no evidence suggesting that the effect of intervention group on the index hospital length of stay depends on ethnicity (p-value = 0.657).

**Area Deprivation Index (Quartiles)**

```
##
## Model 1: los_hrs ~ Group.Assignment. * quartile_grp + rcs(AGE, 3) +
RACE_2 +
##     ETHNICITY
## Model 2: los_hrs ~ Group.Assignment. + rcs(AGE, 3) + RACE_2 + ETHNI
CITY +
##     quartile_grp
##
## L.R. Chisq       d.f.          P
##  0.1354324  3.0000000  0.9872701
```

There is no evidence suggesting that the effect of intervention group on the index hospital length of stay depends on area deprivation index (p-value = 0.987).

## Secondary Analysis

- Continuous outcomes will be compared between study groups like the primary outcome: proportional odds model
- Binary endpoints will use a logit link function
- For counts either a zero-inflated Poisson or a negative binomial model will be fit.
- If there are missing outcomes, these may be imputed if they occur in less than 5% of cases. Otherwise, the cohort for which the outcome is available will be described, along with the results of the model evaluating treatment effects in this cohort.
- No adjustments for multiplicity will be made.
- Missing data for covariates will be imputed using single imputation
    - Modes will be imputed for categorical variables
    - Means will be imputed for continuous variables

# Number of readmissions

## Unadjusted - Proportional odds model

- Outcome: number of readmissions
- Predictor: Group assignment (Bridge Clinic or usual care)
- Model: proportional odds model
- Number of observations included in this analysis: 335

| | Low | High | Diff. | Effect | S.E. | Lower 0.95 | Upper 0.95 | Type |
|---|---|---|---|---|---|---|---|---|
| Group Assignment (Bridge Clinic vs Usual Care) | 1 | 2 | | 0.77 | 0.28 | 0.23 | 1.31 | 1 |
| Odds Ratio | 1 | 2 | | 2.15 | | 1.25 | 3.71 | 2 |

The unadjusted OR (95% CI) for the number of readmissions being greater for a subject in Bridge Clinic vs Usual Care is 2.155 (1.252-3.707).

## Adjusted - Proportional odds model

- Proportional odds model
- Single imputation was used to impute missing data for race and ethnicity
- Outcome: number of readmissions
- Predictor: Group assignment (Bridge Clinic or usual care)
- Adjusted for age, race, ethnicity, area deprivation index (quartiles)
  - Restricted cubic splines are used for continuous covariates (with 3 knots)
    - Age
- Number of observations included in this analysis: 335

| | Low | High | Diff. | Effect | S.E. | Lower 0.95 | Upper 0.95 | Type |
|---|---|---|---|---|---|---|---|---|
| Age | 31.93 | 45.72 | 13.79 | 0.25 | 0.24 | -0.22 | 0.71 | 1 |
| Odds Ratio | 31.93 | 45.72 | 13.79 | 1.28 | | 0.81 | 2.03 | 2 |
| Group Assignment (Bridge Clinic vs Usual Care) | 1.00 | 2.00 | | 0.77 | 0.28 | 0.22 | 1.32 | 1 |
| Odds Ratio | 1.00 | 2.00 | | 2.17 | | 1.25 | 3.76 | 2 |
| Race (Black or African American vs White) | 1.00 | 2.00 | | -0.71 | 0.52 | -1.72 | 0.30 | 1 |

| | Low | High | Diff. | Effect | S.E. | Lower 0.95 | Upper 0.95 | Type |
|---|---|---|---|---|---|---|---|---|
| Odds Ratio | 1.00 | 2.00 | | 0.49 | | 0.18 | 1.35 | 2 |
| Race (Other vs White) | 1.00 | 3.00 | | -7.23 | 33.27 | -72.43 | 57.97 | 1 |
| Odds Ratio | 1.00 | 3.00 | | 0.00 | | 0.00 | 1.506e+25 | 2 |
| Ethnicity (Hispanic/Latino vs Non-Hispanic/Latino) | 2.00 | 1.00 | | -7.37 | 31.33 | -68.79 | 54.04 | 1 |
| Odds Ratio | 2.00 | 1.00 | | 0.00 | | 0.00 | 2.946e+23 | 2 |
| ADI Quartile (2 vs 1) | 1.00 | 2.00 | | 0.27 | 0.37 | -0.46 | 1.00 | 1 |
| Odds Ratio | 1.00 | 2.00 | | 1.31 | | 0.63 | 2.72 | 2 |
| ADI Quartile (3 vs 1) | 1.00 | 3.00 | | -0.53 | 0.42 | -1.35 | 0.29 | 1 |
| Odds Ratio | 1.00 | 3.00 | | 0.59 | | 0.26 | 1.33 | 2 |
| ADI Quartile (4 vs 1) | 1.00 | 4.00 | | -0.03 | 0.36 | -0.73 | 0.66 | 1 |
| Odds Ratio | 1.00 | 4.00 | | 0.97 | | 0.48 | 1.94 | 2 |

The adjusted OR (95% CI) for the number of readmissions being greater for a subject in Bridge Clinic vs Usual Care is 2.165 (1.246-3.762).

# Number of ED visits

## Unadjusted - Proportional odds model

- Outcome: number of ED visits
- Predictor: Group assignment (Bridge Clinic or usual care)
- Model: proportional odds model
- Number of observations included in this analysis: 335

| | Low | High | Diff. | Effect | S.E. | Lower 0.95 | Upper 0.95 | Type |
|---|---|---|---|---|---|---|---|---|
| Group Assignment (Bridge Clinic vs Usual Care) | 1 | 2 | | 0.15 | 0.26 | -0.36 | 0.66 | 1 |
| Odds Ratio | 1 | 2 | | 1.16 | | 0.70 | 1.93 | 2 |

The unadjusted OR (95% CI) for the number of ED visits being greater for a subject in Bridge Clinic vs Usual Care is 1.162 (0.699-1.931).

## Adjusted - Proportional odds model

- Proportional odds model
- Single imputation was used to impute missing data for race and ethnicity
- Outcome: number of ED visits
- Predictor: Group assignment (Bridge Clinic or usual care)
- Adjusted for age, race, ethnicity, area deprivation index (quartiles)
    - Restricted cubic splines are used for continuous covariates (with 3 knots)
        - Age
- Number of observations included in this analysis: 335

| | Low | High | Diff. | Effect | S.E. | Lower 0.95 | Upper 0.95 | Type |
|---|---|---|---|---|---|---|---|---|
| Age | 31.93 | 45.72 | 13.79 | 0.45 | 0.23 | -0.01 | 0.90 | 1 |
| Odds Ratio | 31.93 | 45.72 | 13.79 | 1.57 | | 0.99 | 2.46 | 2 |
| Group Assignment (Bridge Clinic vs Usual Care) | 1.00 | 2.00 | | 0.14 | 0.27 | -0.38 | 0.66 | 1 |
| Odds Ratio | 1.00 | 2.00 | | 1.15 | | 0.68 | 1.94 | 2 |
| Race (Black or African American vs White) | 1.00 | 2.00 | | 0.48 | 0.39 | -0.29 | 1.25 | 1 |
| Odds Ratio | 1.00 | 2.00 | | 1.61 | | 0.75 | 3.50 | 2 |

| | Low | High | Diff. | Effect | S.E. | Lower 0.95 | Upper 0.95 | Type |
|---|---|---|---|---|---|---|---|---|
| Race (Other vs White) | 1.00 | 3.00 | | -0.38 | 1.22 | -2.78 | 2.02 | 1 |
| Odds Ratio | 1.00 | 3.00 | | 0.68 | | 0.06 | 7.53 | 2 |
| Ethnicity (Hispanic/Latino vs Non-Hispanic/Latino) | 2.00 | 1.00 | | -0.91 | 1.20 | -3.26 | 1.44 | 1 |
| Odds Ratio | 2.00 | 1.00 | | 0.40 | | 0.04 | 4.22 | 2 |
| ADI Quartile (2 vs 1) | 1.00 | 2.00 | | 0.17 | 0.34 | -0.51 | 0.84 | 1 |
| Odds Ratio | 1.00 | 2.00 | | 1.18 | | 0.60 | 2.32 | 2 |
| ADI Quartile (3 vs 1) | 1.00 | 3.00 | | -0.67 | 0.40 | -1.44 | 0.11 | 1 |
| Odds Ratio | 1.00 | 3.00 | | 0.51 | | 0.24 | 1.12 | 2 |
| ADI Quartile (4 vs 1) | 1.00 | 4.00 | | -0.49 | 0.37 | -1.20 | 0.23 | 1 |
| Odds Ratio | 1.00 | 4.00 | | 0.62 | | 0.30 | 1.26 | 2 |

The adjusted OR (95% CI) for the number of ED visits being greater for a subject in Bridge Clinic vs Usual Care is 1.151 (0.683-1.942).

## Number of readmissions or ED visits

### Unadjusted - Proportional odds model

- Outcome: number of readmissions or ED visits
- Predictor: Group assignment (Bridge Clinic or usual care)
- Model: proportional odds model
- Number of observations included in this analysis: 335

| | Low | High | Diff. | Effect | S.E. | Lower 0.95 | Upper 0.95 | Type |
|---|---|---|---|---|---|---|---|---|
| Group Assignment (Bridge Clinic vs Usual Care) | 1 | 2 | | 0.50 | 0.22 | 0.06 | 0.93 | 1 |
| Odds Ratio | 1 | 2 | | 1.64 | | 1.06 | 2.55 | 2 |

The unadjusted OR (95% CI) for the number of readmissions or ED visits being greater for a subject in Bridge Clinic vs Usual Care is 1.643 (1.06-2.547).

### Adjusted - Proportional Odds Model

- Proportional odds model
- Single imputation was used to impute missing data for race and ethnicity
- Outcome: number of readmissions or ED visits
- Predictor: Group assignment (Bridge Clinic or usual care)
- Adjusted for age, race, ethnicity, area deprivation index (quartiles)
    - Restricted cubic splines are used for continuous covariates (with 3 knots)
        - Age
- Number of observations included in this analysis: 335

| | Low | High | Diff. | Effect | S.E. | Lower 0.95 | Upper 0.95 | Type |
|---|---|---|---|---|---|---|---|---|
| Age | 31.93 | 45.72 | 13.79 | 0.32 | 0.19 | -0.05 | 0.70 | 1 |
| Odds Ratio | 31.93 | 45.72 | 13.79 | 1.38 | | 0.95 | 2.01 | 2 |
| Group Assignment (Bridge Clinic vs Usual Care) | 1.00 | 2.00 | | 0.51 | 0.23 | 0.06 | 0.96 | 1 |
| Odds Ratio | 1.00 | 2.00 | | 1.66 | | 1.06 | 2.60 | 2 |
| Race (Black or African American vs White) | 1.00 | 2.00 | | 0.12 | 0.36 | -0.59 | 0.83 | 1 |
| Odds Ratio | 1.00 | 2.00 | | 1.12 | | 0.55 | 2.29 | 2 |

| | Low | High | Diff. | Effect | S.E. | Lower 0.95 | Upper 0.95 | Type |
|---|---|---|---|---|---|---|---|---|
| Race (Other vs White) | 1.00 | 3.00 | | -1.00 | 1.20 | -3.34 | 1.35 | 1 |
| Odds Ratio | 1.00 | 3.00 | | 0.37 | | 0.04 | 3.86 | 2 |
| Ethnicity (Hispanic/Latino vs Non-Hispanic/Latino) | 2.00 | 1.00 | | -1.48 | 1.17 | -3.76 | 0.81 | 1 |
| Odds Ratio | 2.00 | 1.00 | | 0.23 | | 0.02 | 2.25 | 2 |
| ADI Quartile (2 vs 1) | 1.00 | 2.00 | | 0.10 | 0.30 | -0.49 | 0.70 | 1 |
| Odds Ratio | 1.00 | 2.00 | | 1.11 | | 0.61 | 2.01 | 2 |
| ADI Quartile (3 vs 1) | 1.00 | 3.00 | | -0.85 | 0.34 | -1.52 | -0.17 | 1 |
| Odds Ratio | 1.00 | 3.00 | | 0.43 | | 0.22 | 0.84 | 2 |
| ADI Quartile (4 vs 1) | 1.00 | 4.00 | | -0.37 | 0.30 | -0.96 | 0.23 | 1 |
| Odds Ratio | 1.00 | 4.00 | | 0.69 | | 0.38 | 1.25 | 2 |

The adjusted OR (95% CI) for the number of readmissions or ED visits being greater for a subject in Bridge Clinic vs Usual Care is 1.661 (1.061-2.6).

## Recurrent opioid use

### Unadjusted

- Outcome: recurrent opioid use (yes/no)
- Predictor: Group assignment (Bridge Clinic or usual care)
- Model: binary logistic regression model
- Number of observations included in this analysis: 88

| | Low | High | Diff. | Effect | S.E. | Lower 0.95 | Upper 0.95 | Type |
|---|---|---|---|---|---|---|---|---|
| Group Assignment (Bridge Clinic vs Usual Care) | 1 | 2 | | -0.24 | 0.45 | -1.11 | 0.64 | 1 |
| Odds Ratio | 1 | 2 | | 0.79 | | 0.33 | 1.89 | 2 |

The unadjusted OR (95% CI) for a subject in Bridge Clinic to have recurrent opioid use vs a subject with usual care is 0.79 (0.329-1.895).

### Adjusted

- Binary logistic regression model
- Single imputation was used to impute missing data for race and ethnicity
- Outcome: recurrent opioid use (yes/no)
- Predictor: Group assignment (Bridge Clinic or usual care)
- Adjusted for age, race, ethnicity, area deprivation index (quartiles)
  - Restricted cubic splines are used for continuous covariates (with 3 knots)
    - Age
- Number of observations included in this analysis: 88

| | Low | High | Diff. | Effect | S.E. | Lower 0.95 | Upper 0.95 | Type |
|---|---|---|---|---|---|---|---|---|
| Age | 31.93 | 45.72 | 13.79 | 0.08 | 0.49 | -0.87 | 1.04 | 1 |
| Odds Ratio | 31.93 | 45.72 | 13.79 | 1.09 | | 0.42 | 2.83 | 2 |
| Group Assignment (Bridge Clinic vs Usual Care) | 1.00 | 2.00 | | -0.31 | 0.50 | -1.29 | 0.68 | 1 |
| Odds Ratio | 1.00 | 2.00 | | 0.74 | | 0.28 | 1.97 | 2 |
| Race (Black or African American vs White) | 1.00 | 2.00 | | 0.94 | 0.78 | -0.58 | 2.46 | 1 |

| | Low | High | Diff. | Effect | S.E. | Lower 0.95 | Upper 0.95 | Type |
|---|---|---|---|---|---|---|---|---|
| Odds Ratio | 1.00 | 2.00 | | 2.56 | | 0.56 | 11.75 | 2 |
| Race (Other vs White) | 1.00 | 3.00 | | 0.54 | 1.72 | -2.84 | 3.91 | 1 |
| Odds Ratio | 1.00 | 3.00 | | 1.71 | | 0.06 | 50.10 | 2 |
| Ethnicity (Hispanic/Latino vs Non-Hispanic/Latino) | 2.00 | 1.00 | | -0.61 | 1.88 | -4.31 | 3.08 | 1 |
| Odds Ratio | 2.00 | 1.00 | | 0.54 | | 0.01 | 21.73 | 2 |
| ADI Quartile (2 vs 1) | 1.00 | 2.00 | | -0.32 | 0.66 | -1.62 | 0.98 | 1 |
| Odds Ratio | 1.00 | 2.00 | | 0.73 | | 0.20 | 2.67 | 2 |
| ADI Quartile (3 vs 1) | 1.00 | 3.00 | | 0.67 | 0.64 | -0.58 | 1.92 | 1 |
| Odds Ratio | 1.00 | 3.00 | | 1.95 | | 0.56 | 6.82 | 2 |
| ADI Quartile (4 vs 1) | 1.00 | 4.00 | | -0.66 | 0.62 | -1.87 | 0.55 | 1 |
| Odds Ratio | 1.00 | 4.00 | | 0.52 | | 0.15 | 1.72 | 2 |

The adjusted OR (95% CI) for a subject in Bridge Clinic to have recurrent opioid use vs a subject with usual care is 0.736 (0.276-1.966).

## Opioid use in last 30 days

### Unadjusted

- Outcome: opioid use in past 30 days (no use, single use, multiple use)
- Predictor: Group assignment (Bridge Clinic or usual care)
- Model: proportional odds model
- Number of observations included in this analysis: 32

| | Low | High | Diff. | Effect | S.E. | Lower 0.95 | Upper 0.95 | Type |
|---|---|---|---|---|---|---|---|---|
| Group Assignment (Bridge Clinic vs Usual Care) | 1 | 2 | | 1.76 | 0.92 | -0.03 | 3.56 | 1 |
| Odds Ratio | 1 | 2 | | 5.82 | | 0.97 | 35.09 | 2 |

The unadjusted OR (95% CI) for a subject in Bridge Clinic to have more opioid uses in the past 30 days vs a subject with usual care is 5.822 (0.966-35.093).

### Adjusted

- Proportional odds model
- Single imputation was used to impute missing data for race and ethnicity
- Outcome: opioid use in past 30 days (no use, single use, multiple use)
- Predictor: Group assignment (Bridge Clinic or usual care)
- Adjusted for age, race, area deprivation index (quartiles)
  - Restricted cubic splines are used for continuous covariates (with 3 knots)
    - Age
  - This model was not adjusted for ethnicity because all subjects in this model (N = 32) are Non-Hispanic/Latino
- Number of observations included in this analysis: 32

| | Low | High | Diff. | Effect | S.E. | Lower 0.95 | Upper 0.95 | Type |
|---|---|---|---|---|---|---|---|---|
| Age | 31.93 | 45.72 | 13.79 | 2.45 | 1.24 | 0.02 | 4.88 | 1 |
| Odds Ratio | 31.93 | 45.72 | 13.79 | 11.62 | | 1.02 | 132.03 | 2 |
| Group Assignment (Bridge Clinic vs Usual Care) | 1.00 | 2.00 | | 3.78 | 1.55 | 0.75 | 6.81 | 1 |
| Odds Ratio | 1.00 | 2.00 | | 43.76 | | 2.11 | 906.46 | 2 |
| Race (Black or African | 1.00 | 2.00 | | 2.49 | 1.74 | -0.93 | 5.90 | 1 |

| | Low | High | Diff. | Effect | S.E. | Lower 0.95 | Upper 0.95 | Type |
|---|---|---|---|---|---|---|---|---|
| American vs White) | | | | | | | | |
| Odds Ratio | 1.00 | 2.00 | | 12.04 | | 0.40 | 366.03 | 2 |
| Race (Other vs White) | 1.00 | 3.00 | | 9.11 | 72.48 | -132.94 | 151.17 | 1 |
| Odds Ratio | 1.00 | 3.00 | | 9,090.16 | | 0.00 | 4.496e+65 | 2 |
| ADI Quartile (2 vs 1) | 1.00 | 2.00 | | -0.01 | 1.57 | -3.10 | 3.07 | 1 |
| Odds Ratio | 1.00 | 2.00 | | 0.99 | | 0.05 | 21.57 | 2 |
| ADI Quartile (3 vs 1) | 1.00 | 3.00 | | -1.06 | 1.74 | -4.47 | 2.35 | 1 |
| Odds Ratio | 1.00 | 3.00 | | 0.35 | | 0.01 | 10.51 | 2 |
| ADI Quartile (4 vs 1) | 1.00 | 4.00 | | 8.48 | 27.07 | -44.57 | 61.54 | 1 |
| Odds Ratio | 1.00 | 4.00 | | 4,829.69 | | 0.00 | 5.310e+26 | 2 |

The adjusted OR (95% CI) for a subject in Bridge Clinic to have more opioid uses in the past 30 days vs a subject with usual care is 43.758 (2.112-906.462).

## Total cost of care

### Unadjusted

- Outcome: total cost of care

- Predictor: Group assignment (Bridge Clinic or usual care)
- Model: proportional odds model
- Number of observations included in this analysis: 311

| | Low | High | Diff. | Effect | S.E. | Lower 0.95 | Upper 0.95 | Type |
|---|---|---|---|---|---|---|---|---|
| Group Assignment (Bridge Clinic vs Usual Care) | 1 | 2 | | 0.82 | 0.2 | 0.42 | 1.21 | 1 |
| Odds Ratio | 1 | 2 | | 2.27 | | 1.53 | 3.36 | 2 |

The unadjusted OR (95% CI) for the total cost of care being greater for a subject in Bridge Clinic vs Usual Care is 2.265 (1.525-3.365).

## Adjusted

- Proportional odds model
- Single imputation was used to impute missing data for race and ethnicity
- Outcome: total cost of care
- Predictor: Group assignment (Bridge Clinic or usual care)
- Adjusted for age, race, ethnicity, area deprivation index (quartiles)
    - Restricted cubic splines are used for continuous covariates (with 3 knots)
        - Age
- Number of observations included in this analysis: 311

| | Low | High | Diff. | Effect | S.E. | Lower 0.95 | Upper 0.95 | Type |
|---|---|---|---|---|---|---|---|---|
| Age | 31.93 | 45.72 | 13.79 | 0.53 | 0.16 | 0.21 | 0.86 | 1 |
| Odds Ratio | 31.93 | 45.72 | 13.79 | 1.71 | | 1.24 | 2.35 | 2 |
| Group Assignment (Bridge Clinic vs Usual Care) | 1.00 | 2.00 | | 0.81 | 0.20 | 0.41 | 1.21 | 1 |
| Odds Ratio | 1.00 | 2.00 | | 2.25 | | 1.51 | 3.35 | 2 |
| Race (Black or African American vs White) | 1.00 | 2.00 | | -0.05 | 0.32 | -0.68 | 0.58 | 1 |
| Odds Ratio | 1.00 | 2.00 | | 0.95 | | 0.50 | 1.79 | 2 |
| Race (Other vs White) | 1.00 | 3.00 | | 0.14 | 0.73 | -1.29 | 1.56 | 1 |
| Odds Ratio | 1.00 | 3.00 | | 1.15 | | 0.27 | 4.78 | 2 |
| Ethnicity (Hispanic/Latino vs Non- | 2.00 | 1.00 | | -0.25 | 0.68 | -1.58 | 1.08 | 1 |

| | Low | High | Diff. | Effect | S.E. | Lower 0.95 | Upper 0.95 | Type |
|---|---|---|---|---|---|---|---|---|
| Hispanic/Latino) | | | | | | | | |
| Odds Ratio | 2.00 | 1.00 | | 0.78 | | 0.21 | 2.93 | 2 |
| ADI Quartile (2 vs 1) | 1.00 | 2.00 | | 0.05 | 0.28 | -0.50 | 0.59 | 1 |
| Odds Ratio | 1.00 | 2.00 | | 1.05 | | 0.61 | 1.80 | 2 |
| ADI Quartile (3 vs 1) | 1.00 | 3.00 | | -0.62 | 0.27 | -1.15 | -0.08 | 1 |
| Odds Ratio | 1.00 | 3.00 | | 0.54 | | 0.32 | 0.92 | 2 |
| ADI Quartile (4 vs 1) | 1.00 | 4.00 | | -0.39 | 0.26 | -0.91 | 0.12 | 1 |
| Odds Ratio | 1.00 | 4.00 | | 0.68 | | 0.40 | 1.13 | 2 |

The adjusted OR (95% CI) for the total cost of care being greater for a subject in Bridge Clinic vs Usual Care is 2.25 (1.513-3.346).

## Linkage to MOUD provider

### Unadjusted

- Outcome: Linkage to MOUD provider (yes/no)
- Predictor: Group assignment (Bridge Clinic or usual care)
- Model: binary logistic regression model
- Number of observations included in this analysis: 88

| | Low | High | Diff. | Effect | S.E. | Lower 0.95 | Upper 0.95 | Type |
|---|---|---|---|---|---|---|---|---|
| Group Assignment (Bridge Clinic vs Usual Care) | 1 | 2 | | 1.03 | 0.49 | 0.06 | 1.99 | 1 |
| Odds Ratio | 1 | 2 | | 2.80 | | 1.07 | 7.35 | 2 |

The unadjusted OR (95% CI) for a subject in Bridge Clinic to attend at least one visit with a MOUD provider vs a subject in usual care is 2.799 (1.066-7.351).

### Adjusted

- Binary logistic regression model
- Single imputation was used to impute missing data for race and ethnicity
- Outcome: Linkage to MOUD provider (yes/no)
- Predictor: Group assignment (Bridge Clinic or usual care)
- Adjusted for age, race, ethnicity, area deprivation index (quartiles)
  - Restricted cubic splines are used for continuous covariates (with 3 knots)
    - Age
- Number of observations included in this analysis: 88

| | Low | High | Diff. | Effect | S.E. | Lower 0.95 | Upper 0.95 | Type |
|---|---|---|---|---|---|---|---|---|
| Age | 31.93 | 45.72 | 13.79 | 0.54 | 0.56 | -0.55 | 1.63 | 1 |
| Odds Ratio | 31.93 | 45.72 | 13.79 | 1.72 | | 0.58 | 5.10 | 2 |
| Group Assignment (Bridge Clinic vs Usual Care) | 1.00 | 2.00 | | 0.92 | 0.57 | -0.19 | 2.03 | 1 |
| Odds Ratio | 1.00 | 2.00 | | 2.51 | | 0.82 | 7.64 | 2 |
| Race (Black or African American vs White) | 1.00 | 2.00 | | -1.56 | 0.85 | -3.23 | 0.10 | 1 |
| Odds Ratio | 1.00 | 2.00 | | 0.21 | | 0.04 | 1.10 | 2 |
| Race (Other vs White) | 1.00 | 3.00 | | 13.79 | 57.02 | -97.97 | 125.54 | 1 |
| Odds Ratio | 1.00 | 3.00 | | 9.702e+05 | | 0.00 | 3.328e+54 | 2 |
| Ethnicity (Hispanic/Latino vs Non-Hispanic/Latino) | 2.00 | 1.00 | | -6.92 | 41.87 | -88.99 | 75.15 | 1 |
| Odds Ratio | 2.00 | 1.00 | | 0.00 | | 0.00 | 4.338e+32 | 2 |
| ADI Quartile (2 vs 1) | 1.00 | 2.00 | | 1.03 | 0.85 | -0.64 | 2.70 | 1 |

| | Low | High | Diff. | Effect | S.E. | Lower 0.95 | Upper 0.95 | Type |
|---|---|---|---|---|---|---|---|---|
| Odds Ratio | 1.00 | 2.00 | | 2.79 | | 0.53 | 14.87 | 2 |
| ADI Quartile (3 vs 1) | 1.00 | 3.00 | | -0.54 | 0.72 | -1.95 | 0.87 | 1 |
| Odds Ratio | 1.00 | 3.00 | | 0.58 | | 0.14 | 2.38 | 2 |
| ADI Quartile (4 vs 1) | 1.00 | 4.00 | | 0.09 | 0.70 | -1.27 | 1.45 | 1 |
| Odds Ratio | 1.00 | 4.00 | | 1.09 | | 0.28 | 4.28 | 2 |

The adjusted OR (95% CI) for a subject in Bridge Clinic to attend at least one visit with a MOUD provider vs a subject in usual care is 2.509 (0.824-7.64).

## Quality of life

### Unadjusted

- Outcome: quality of life score
- Predictor: Group assignment (Bridge Clinic or usual care)
- Model: proportional odds model
- Number of observations included in this analysis: 86

| | Low | High | Diff. | Effect | S.E. | Lower 0.95 | Upper 0.95 | Type |
|---|---|---|---|---|---|---|---|---|
| Group Assignment (Bridge Clinic vs Usual Care) | 1 | 2 | | 0.17 | 0.38 | -0.58 | 0.92 | 1 |
| Odds Ratio | 1 | 2 | | 1.18 | | 0.56 | 2.50 | 2 |

The unadjusted OR (95% CI) for the quality of life score being greater for a subject in Bridge Clinic vs Usual Care is 1.181 (0.558-2.498).

### Adjusted

- Proportional odds mode1
- Single imputation was used to impute missing data for race and ethnicity
- Outcome: quality of life score
- Predictor: Group assignment (Bridge Clinic or usual care)
- Adjusted for age, race, ethnicity, area deprivation index (quartiles)
  - Restricted cubic splines are used for continuous covariates (with 3 knots)
    - Age
- Number of observations included in this analysis: 86

| | Low | High | Diff. | Effect | S.E. | Lower 0.95 | Upper 0.95 | Type |
|---|---|---|---|---|---|---|---|---|
| Age | 31.93 | 45.72 | 13.79 | -0.42 | 0.43 | -1.27 | 0.43 | 1 |
| Odds Ratio | 31.93 | 45.72 | 13.79 | 0.66 | | 0.28 | 1.53 | 2 |
| Group Assignment (Bridge Clinic vs Usual Care) | 1.00 | 2.00 | | 0.02 | 0.41 | -0.79 | 0.83 | 1 |
| Odds Ratio | 1.00 | 2.00 | | 1.02 | | 0.45 | 2.29 | 2 |
| Race (Black or African American vs White) | 1.00 | 2.00 | | -0.84 | 0.59 | -2.00 | 0.32 | 1 |

| | Low | High | Diff. | Effect | S.E. | Lower 0.95 | Upper 0.95 | Type |
|---|---|---|---|---|---|---|---|---|
| Odds Ratio | 1.00 | 2.00 | | 0.43 | | 0.14 | 1.38 | 2 |
| Race (Other vs White) | 1.00 | 3.00 | | -2.17 | 1.27 | -4.67 | 0.32 | 1 |
| Odds Ratio | 1.00 | 3.00 | | 0.11 | | 0.01 | 1.38 | 2 |
| Ethnicity (Hispanic/Latino vs Non-Hispanic/Latino) | 2.00 | 1.00 | | 0.76 | 1.42 | -2.03 | 3.54 | 1 |
| Odds Ratio | 2.00 | 1.00 | | 2.13 | | 0.13 | 34.60 | 2 |
| ADI Quartile (2 vs 1) | 1.00 | 2.00 | | -0.25 | 0.57 | -1.37 | 0.87 | 1 |
| Odds Ratio | 1.00 | 2.00 | | 0.78 | | 0.25 | 2.39 | 2 |
| ADI Quartile (3 vs 1) | 1.00 | 3.00 | | -0.50 | 0.56 | -1.59 | 0.60 | 1 |
| Odds Ratio | 1.00 | 3.00 | | 0.61 | | 0.20 | 1.82 | 2 |
| ADI Quartile (4 vs 1) | 1.00 | 4.00 | | -0.10 | 0.51 | -1.10 | 0.91 | 1 |
| Odds Ratio | 1.00 | 4.00 | | 0.91 | | 0.33 | 2.47 | 2 |

The adjusted OR (95% CI) for the quality of life score being greater for a subject in Bridge Clinic vs Usual Care is 1.019 (0.453-2.295).

# Overdose

## Unadjusted

- Outcome: Overdose (yes/no)
- Predictor: Group assignment (Bridge Clinic or usual care)
- Model: binary logistic regression model
- Number of observations included in this analysis: 86

| | Low | High | Diff. | Effect | S.E. | Lower 0.95 | Upper 0.95 | Type |
|---|---|---|---|---|---|---|---|---|
| Group Assignment (Bridge Clinic vs Usual Care) | 1 | 2 | | -2.02 | 1.14 | -4.26 | 0.21 | 1 |
| Odds Ratio | 1 | 2 | | 0.13 | | 0.01 | 1.24 | 2 |

The unadjusted OR (95% CI) for a subject in Bridge Clinic to overdose vs a subject in usual care is 0.132 (0.014-1.239).

## Adjusted

- Binary logistic regression model
- Single imputation was used to impute missing data for race and ethnicity
- Outcome: Overdose (yes/no)
- Predictor: Group assignment (Bridge Clinic or usual care)
- Adjusted for age, race, ethnicity, area deprivation index (quartiles)
  - Restricted cubic splines are used for continuous covariates (with 3 knots)
    - Age
- Number of observations included in this analysis: 86

| | Low | High | Diff. | Effect | S.E. | Lower 0.95 | Upper 0.95 | Type |
|---|---|---|---|---|---|---|---|---|
| Age | 31.93 | 45.72 | 13.79 | -1.51 | 1.73 | -4.90 | 1.88 | 1 |
| Odds Ratio | 31.93 | 45.72 | 13.79 | 0.22 | | 0.01 | 6.53 | 2 |
| Group Assignment (Bridge Clinic vs Usual Care) | 1.00 | 2.00 | | -2.11 | 1.33 | -4.72 | 0.51 | 1 |
| Odds Ratio | 1.00 | 2.00 | | 0.12 | | 0.01 | 1.66 | 2 |
| Race (Black or African American vs | 1.00 | 2.00 | | -0.28 | 1.36 | -2.94 | 2.38 | 1 |

| | Low | High | Diff. | Effect | S.E. | Lower 0.95 | Upper 0.95 | Type |
|---|---|---|---|---|---|---|---|---|
| White) | | | | | | | | |
| Odds Ratio | 1.00 | 2.00 | | 0.76 | | 0.05 | 10.80 | 2 |
| Race (Other vs White) | 1.00 | 3.00 | | -3.71 | 135.96 | -270.19 | 262.77 | 1 |
| Odds Ratio | 1.00 | 3.00 | | 0.02 | | 0.00 | 1.315e+114 | 2 |
| Ethnicity (Hispanic/Latino vs Non-Hispanic/Latino) | 2.00 | 1.00 | | -8.63 | 135.95 | -275.08 | 257.82 | 1 |
| Odds Ratio | 2.00 | 1.00 | | 0.00 | | 0.00 | 9.335e+111 | 2 |
| ADI Quartile (2 vs 1) | 1.00 | 2.00 | | 0.63 | 1.45 | -2.21 | 3.46 | 1 |
| Odds Ratio | 1.00 | 2.00 | | 1.87 | | 0.11 | 31.91 | 2 |
| ADI Quartile (3 vs 1) | 1.00 | 3.00 | | 0.84 | 1.40 | -1.90 | 3.58 | 1 |
| Odds Ratio | 1.00 | 3.00 | | 2.32 | | 0.15 | 35.89 | 2 |
| ADI Quartile (4 vs 1) | 1.00 | 4.00 | | -7.84 | 35.31 | -77.05 | 61.36 | 1 |
| Odds Ratio | 1.00 | 4.00 | | 0.00 | | 0.00 | 4.465e+26 | 2 |

The adjusted OR (95% CI) for a subject in Bridge Clinic to overdose vs a subject in usual care is 0.122 (0.009-1.659).

## Death

### Unadjusted

- Outcome: Death (yes/no)

- Predictor: Group assignment (Bridge Clinic or usual care)
- Model: binary logistic regression model
- Number of observations included in this analysis: 335

| | Low | High | Diff. | Effect | S.E. | Lower 0.95 | Upper 0.95 | Type |
|---|---|---|---|---|---|---|---|---|
| Group Assignment (Bridge Clinic vs Usual Care) | 1 | 2 | | -0.7 | 0.87 | -2.41 | 1.01 | 1 |
| Odds Ratio | 1 | 2 | | 0.5 | | 0.09 | 2.75 | 2 |

The unadjusted OR (95% CI) for a subject in Bridge Clinic to die within the 16 week follow up period vs a subject in usual care is 0.497 (0.09-2.751).

### Adjusted

- Binary logistic regression model
- Single imputation was used to impute missing data for race and ethnicity
- Outcome: Death (yes/no)
- Predictor: Group assignment (Bridge Clinic or usual care)
- Adjusted for age, race, ethnicity, area deprivation index (quartiles)
  - Restricted cubic splines are used for continuous covariates (with 3 knots)
    - Age
- Number of observations included in this analysis: 335

| | Low | High | Diff. | Effect | S.E. | Lower 0.95 | Upper 0.95 | Type |
|---|---|---|---|---|---|---|---|---|
| Age | 31.93 | 45.72 | 13.79 | 0.50 | 0.81 | -1.09 | 2.09 | 1 |
| Odds Ratio | 31.93 | 45.72 | 13.79 | 1.65 | | 0.34 | 8.09 | 2 |
| Group Assignment (Bridge Clinic vs Usual Care) | 1.00 | 2.00 | | -0.74 | 0.89 | -2.49 | 1.00 | 1 |
| Odds Ratio | 1.00 | 2.00 | | 0.47 | | 0.08 | 2.72 | 2 |
| Race (Black or African American vs White) | 1.00 | 2.00 | | 0.29 | 1.17 | -2.00 | 2.58 | 1 |
| Odds Ratio | 1.00 | 2.00 | | 1.34 | | 0.13 | 13.25 | 2 |
| Race (Other vs White) | 1.00 | 3.00 | | -4.55 | 60.88 | -123.88 | 114.77 | 1 |
| Odds Ratio | 1.00 | 3.00 | | 0.01 | | 0.00 | 6.988e+49 | 2 |

| | Low | High | Diff. | Effect | S.E. | Lower 0.95 | Upper 0.95 | Type |
|---|---|---|---|---|---|---|---|---|
| Ethnicity (Hispanic/Latino vs Non-Hispanic/Latino) | 2.00 | 1.00 | | -5.59 | 58.58 | -120.40 | 109.22 | 1 |
| Odds Ratio | 2.00 | 1.00 | | 0.00 | | 0.00 | 2.703e+47 | 2 |
| ADI Quartile (2 vs 1) | 1.00 | 2.00 | | 7.31 | 21.64 | -35.10 | 49.72 | 1 |
| Odds Ratio | 1.00 | 2.00 | | 1,494.94 | | 0.00 | 3.922e+21 | 2 |
| ADI Quartile (3 vs 1) | 1.00 | 3.00 | | 7.35 | 21.64 | -35.06 | 49.76 | 1 |
| Odds Ratio | 1.00 | 3.00 | | 1,556.76 | | 0.00 | 4.074e+21 | 2 |
| ADI Quartile (4 vs 1) | 1.00 | 4.00 | | 7.19 | 21.64 | -35.22 | 49.60 | 1 |
| Odds Ratio | 1.00 | 4.00 | | 1,324.72 | | 0.00 | 3.466e+21 | 2 |

The adjusted OR (95% CI) for a subject in Bridge Clinic to die within the 16 week follow up period vs a subject in usual care is 0.475 (0.083-2.721).

## Hospital free days

### Unadjusted

- Outcome: hospital free days
- Predictor: Group assignment (Bridge Clinic or usual care)
- Model: proportional odds model
- Number of observations included in this analysis: 335

| | Low | High | Diff. | Effect | S.E. | Lower 0.95 | Upper 0.95 | Type |
|---|---|---|---|---|---|---|---|---|
| Group Assignment (Bridge Clinic vs Usual Care) | 1 | 2 | | -0.62 | 0.27 | -1.14 | -0.1 | 1 |
| Odds Ratio | 1 | 2 | | 0.54 | | 0.32 | 0.9 | 2 |

The unadjusted OR (95% CI) for the number of hospital free days being greater for a subject in Bridge Clinic vs Usual Care is 0.537 (0.32-0.904).

## Adjusted

- Proportional odds model
- Single imputation was used to impute missing data for race and ethnicity
- Outcome: hospital free days
- Predictor: Group assignment (Bridge Clinic or usual care)
- Adjusted for age, race, ethnicity, area deprivation index (quartiles)
  - Restricted cubic splines are used for continuous covariates (with 3 knots)
    - Age
- Number of observations included in this analysis: 335

| | Low | High | Diff. | Effect | S.E. | Lower 0.95 | Upper 0.95 | Type |
|---|---|---|---|---|---|---|---|---|
| Age | 31.93 | 45.72 | 13.79 | -0.31 | 0.23 | -0.76 | 0.15 | 1 |
| Odds Ratio | 31.93 | 45.72 | 13.79 | 0.73 | | 0.47 | 1.16 | 2 |
| Group Assignment (Bridge Clinic vs Usual Care) | 1.00 | 2.00 | | -0.61 | 0.27 | -1.14 | -0.08 | 1 |
| Odds Ratio | 1.00 | 2.00 | | 0.54 | | 0.32 | 0.92 | 2 |
| Race (Black or African American vs White) | 1.00 | 2.00 | | 0.48 | 0.48 | -0.46 | 1.42 | 1 |
| Odds Ratio | 1.00 | 2.00 | | 1.62 | | 0.63 | 4.13 | 2 |
| Race (Other vs White) | 1.00 | 3.00 | | 7.17 | 32.70 | -56.92 | 71.27 | 1 |
| Odds Ratio | 1.00 | 3.00 | | 1,304.81 | | 0.00 | 8.924e+30 | 2 |
| Ethnicity (Hispanic/Latino vs Non-Hispanic/Latino) | 2.00 | 1.00 | | 7.31 | 30.83 | -53.11 | 67.73 | 1 |

| | Low | High | Diff. | Effect | S.E. | Lower 0.95 | Upper 0.95 | Type |
|---|---|---|---|---|---|---|---|---|
| Odds Ratio | 2.00 | 1.00 | | 1,494.88 | | 0.00 | 2.607e+29 | 2 |
| ADI Quartile (2 vs 1) | 1.00 | 2.00 | | -0.33 | 0.36 | -1.03 | 0.37 | 1 |
| Odds Ratio | 1.00 | 2.00 | | 0.72 | | 0.36 | 1.45 | 2 |
| ADI Quartile (3 vs 1) | 1.00 | 3.00 | | 0.41 | 0.40 | -0.38 | 1.20 | 1 |
| Odds Ratio | 1.00 | 3.00 | | 1.50 | | 0.68 | 3.31 | 2 |
| ADI Quartile (4 vs 1) | 1.00 | 4.00 | | -0.15 | 0.35 | -0.83 | 0.52 | 1 |
| Odds Ratio | 1.00 | 4.00 | | 0.86 | | 0.44 | 1.69 | 2 |

The adjusted OR (95% CI) for the number of hospital free days being greater for a subject in Bridge Clinic vs Usual Care is 0.543 (0.32-0.92).

## Hospital and ED free days

### Unadjusted

- Outcome: hospital and ED free days
- Predictor: Group assignment (Bridge Clinic or usual care)
- Model: proportional odds model
- Number of observations included in this analysis: 335

| | Low | High | Diff. | Effect | S.E. | Lower 0.95 | Upper 0.95 | Type |
|---|---|---|---|---|---|---|---|---|

| | Low | High | Diff. | Effect | S.E. | Lower 0.95 | Upper 0.95 | Type |
|---|---|---|---|---|---|---|---|---|
| Group Assignment (Bridge Clinic vs Usual Care) | 1 | 2 | | -0.47 | 0.22 | -0.90 | -0.04 | 1 |
| Odds Ratio | 1 | 2 | | 0.62 | | 0.41 | 0.96 | 2 |

The unadjusted OR (95% CI) for the number of hospital and ED free days being greater for a subject in Bridge Clinic vs Usual Care is 0.623 (0.405-0.959).

### Adjusted

- Proportional odds model
- Single imputation was used to impute missing data for race and ethnicity
- Outcome: hospital and ED free days
- Predictor: Group assignment (Bridge Clinic or usual care)
- Adjusted for age, race, ethnicity, area deprivation index (quartiles)
  - Restricted cubic splines are used for continuous covariates (with 3 knots)
    - Age
- Number of observations included in this analysis: 335

| | Low | High | Diff. | Effect | S.E. | Lower 0.95 | Upper 0.95 | Type |
|---|---|---|---|---|---|---|---|---|
| Age | 31.93 | 45.72 | 13.79 | -0.37 | 0.19 | -0.74 | 0.01 | 1 |
| Odds Ratio | 31.93 | 45.72 | 13.79 | 0.69 | | 0.48 | 1.01 | 2 |
| Group Assignment (Bridge Clinic vs Usual Care) | 1.00 | 2.00 | | -0.50 | 0.22 | -0.93 | -0.06 | 1 |
| Odds Ratio | 1.00 | 2.00 | | 0.61 | | 0.39 | 0.95 | 2 |
| Race (Black or African American vs White) | 1.00 | 2.00 | | -0.17 | 0.35 | -0.85 | 0.52 | 1 |
| Odds Ratio | 1.00 | 2.00 | | 0.85 | | 0.43 | 1.68 | 2 |
| Race (Other vs White) | 1.00 | 3.00 | | 1.01 | 1.20 | -1.34 | 3.36 | 1 |
| Odds Ratio | 1.00 | 3.00 | | 2.74 | | 0.26 | 28.76 | 2 |
| Ethnicity (Hispanic/Latino vs Non-Hispanic/Latino) | 2.00 | 1.00 | | 1.45 | 1.16 | -0.83 | 3.73 | 1 |
| Odds Ratio | 2.00 | 1.00 | | 4.26 | | 0.43 | 41.70 | 2 |

| | Low | High | Diff. | Effect | S.E. | Lower 0.95 | Upper 0.95 | Type |
|---|---|---|---|---|---|---|---|---|
| ADI Quartile (2 vs 1) | 1.00 | 2.00 | | -0.18 | 0.29 | -0.75 | 0.40 | 1 |
| Odds Ratio | 1.00 | 2.00 | | 0.84 | | 0.47 | 1.49 | 2 |
| ADI Quartile (3 vs 1) | 1.00 | 3.00 | | 0.73 | 0.34 | 0.07 | 1.39 | 1 |
| Odds Ratio | 1.00 | 3.00 | | 2.08 | | 1.07 | 4.02 | 2 |
| ADI Quartile (4 vs 1) | 1.00 | 4.00 | | 0.15 | 0.30 | -0.43 | 0.73 | 1 |
| Odds Ratio | 1.00 | 4.00 | | 1.16 | | 0.65 | 2.08 | 2 |

The adjusted OR (95% CI) for the number of hospital and ED free days being greater for a subject in Bridge Clinic vs Usual Care is 0.609 (0.393-0.945).

## Number of buprenorphine refills

### Unadjusted

- Outcome: Number of buprenorphine refills
- Predictor: Group assignment (Bridge Clinic or usual care)
- Model: proportional odds model
- Number of observations included in this analysis: 77

| | Low | High | Diff. | Effect | S.E. | Lower 0.95 | Upper 0.95 | Type |
|---|---|---|---|---|---|---|---|---|
| Group Assignment (Bridge Clinic vs Usual Care) | 1 | 2 | | 1.66 | 0.44 | 0.80 | 2.53 | 1 |
| Odds Ratio | 1 | 2 | | 5.28 | | 2.22 | 12.58 | 2 |

The unadjusted OR (95% CI) for the number of buprenorphine refills being greater for a subject in Bridge Clinic vs Usual Care is 5.28 (2.216-12.582).

### Adjusted

- Proportional odds model
- Single imputation was used to impute missing data for race and ethnicity
- Outcome: Number of buprenorphine refills
- Predictor: Group assignment (Bridge Clinic or usual care)
- Adjusted for age, race, ethnicity, area deprivation index (quartiles)
  - Restricted cubic splines are used for continuous covariates (with 3 knots)
    - Age
- Number of observations included in this analysis: 77

| | Low | High | Diff. | Effect | S.E. | Lower 0.95 | Upper 0.95 | Type |
|---|---|---|---|---|---|---|---|---|
| Age | 31.93 | 45.72 | 13.79 | 0.69 | 0.49 | -0.26 | 1.65 | 1 |
| Odds Ratio | 31.93 | 45.72 | 13.79 | 2.00 | | 0.77 | 5.19 | 2 |
| Group Assignment (Bridge Clinic vs Usual Care) | 1.00 | 2.00 | | 1.93 | 0.50 | 0.94 | 2.91 | 1 |
| Odds Ratio | 1.00 | 2.00 | | 6.87 | | 2.56 | 18.42 | 2 |
| Race (Black or African American vs White) | 1.00 | 2.00 | | -1.52 | 0.69 | -2.87 | -0.17 | 1 |

| | Low | High | Diff. | Effect | S.E. | Lower 0.95 | Upper 0.95 | Type |
|---|---|---|---|---|---|---|---|---|
| Odds Ratio | 1.00 | 2.00 | | 0.22 | | 0.06 | 0.85 | 2 |
| Race (Other vs White) | 1.00 | 3.00 | | -0.94 | 1.43 | -3.75 | 1.86 | 1 |
| Odds Ratio | 1.00 | 3.00 | | 0.39 | | 0.02 | 6.43 | 2 |
| Ethnicity (Hispanic/Latino vs Non-Hispanic/Latino) | 2.00 | 1.00 | | -0.58 | 1.83 | -4.15 | 3.00 | 1 |
| Odds Ratio | 2.00 | 1.00 | | 0.56 | | 0.02 | 20.15 | 2 |
| ADI Quartile (2 vs 1) | 1.00 | 2.00 | | 0.68 | 0.66 | -0.60 | 1.97 | 1 |
| Odds Ratio | 1.00 | 2.00 | | 1.98 | | 0.55 | 7.15 | 2 |
| ADI Quartile (3 vs 1) | 1.00 | 3.00 | | 0.11 | 0.61 | -1.08 | 1.30 | 1 |
| Odds Ratio | 1.00 | 3.00 | | 1.12 | | 0.34 | 3.68 | 2 |
| ADI Quartile (4 vs 1) | 1.00 | 4.00 | | 0.12 | 0.55 | -0.96 | 1.20 | 1 |
| Odds Ratio | 1.00 | 4.00 | | 1.13 | | 0.38 | 3.33 | 2 |

The adjusted OR (95% CI) for the number of buprenorphine refills being greater for a subject in Bridge Clinic vs Usual Care is 6.872 (2.564-18.416).

# Implementation Outcome Analysis

- Binary endpoint: will use a logit link function
- Missing data for covariates will be imputed using single imputation
  - Modes will be imputed for categorical variables
  - Means will be imputed for continuous variables

## Acceptance of Bridge Clinic as a bridging provider

### Unadjusted

- Outcome: Acceptance of Bridge Clinic as a bridging provider - attended at least one visit during follow up (yes/no)
- Predictor: Group assignment (Bridge Clinic or usual care)
- Model: binary logistic regression model
- Number of observations included in this analysis: 335

| | Low | High | Diff. | Effect | S.E. | Lower 0.95 | Upper 0.95 | Type |
|---|---|---|---|---|---|---|---|---|
| Group Assignment (Bridge Clinic vs Usual Care) | 1 | 2 | | 5.44 | 1.02 | 3.45 | 7.43 | 1 |
| Odds Ratio | 1 | 2 | | 231.41 | | 31.64 | 1,692.42 | 2 |

The unadjusted OR (95% CI) for a subject in Bridge Clinic to attend at least one Bridge Clinic visit during the 16-week follow up vs a subject in usual care is 231.414 (31.642-1692.424).

### Adjusted

- Binary logistic regression model
- Single imputation was used to impute missing data for race and ethnicity
- Outcome: Acceptance of Bridge Clinic as a bridging provider - attended at least one visit during follow up (yes/no)
- Predictor: Group assignment (Bridge Clinic or usual care)
- Adjusted for age, race, ethnicity, area deprivation index (quartiles)
  - Restricted cubic splines are used for continuous covariates (with 3 knots)
    - Age
- Number of observations included in this analysis: 335

| | Low | High | Diff. | Effect | S.E. | Lower 0.95 | Upper 0.95 | Type |
|---|---|---|---|---|---|---|---|---|
| Age | 31.93 | 45.72 | 13.79 | 0.51 | 0.28 | -0.05 | 1.06 | 1 |

| | Low | High | Diff. | Effect | S.E. | Lower 0.95 | Upper 0.95 | Type |
|---|---|---|---|---|---|---|---|---|
| Odds Ratio | 31.93 | 45.72 | 13.79 | 1.66 | | 0.95 | 2.90 | 2 |
| Group Assignment (Bridge Clinic vs Usual Care) | 1.00 | 2.00 | | 5.66 | 1.05 | 3.60 | 7.72 | 1 |
| Odds Ratio | 1.00 | 2.00 | | 287.16 | | 36.69 | 2,247.69 | 2 |
| Race (Black or African American vs White) | 1.00 | 2.00 | | -1.18 | 0.59 | -2.34 | -0.03 | 1 |
| Odds Ratio | 1.00 | 2.00 | | 0.31 | | 0.10 | 0.97 | 2 |
| Race (Other vs White) | 1.00 | 3.00 | | 3.20 | 2.30 | -1.31 | 7.71 | 1 |
| Odds Ratio | 1.00 | 3.00 | | 24.53 | | 0.27 | 2,221.45 | 2 |
| Ethnicity (Hispanic/Latino vs Non-Hispanic/Latino) | 2.00 | 1.00 | | -4.44 | 2.43 | -9.20 | 0.33 | 1 |
| Odds Ratio | 2.00 | 1.00 | | 0.01 | | 0.00 | 1.39 | 2 |
| ADI Quartile (2 vs 1) | 1.00 | 2.00 | | -0.46 | 0.46 | -1.36 | 0.44 | 1 |
| Odds Ratio | 1.00 | 2.00 | | 0.63 | | 0.26 | 1.55 | 2 |
| ADI Quartile (3 vs 1) | 1.00 | 3.00 | | -0.25 | 0.46 | -1.15 | 0.65 | 1 |
| Odds Ratio | 1.00 | 3.00 | | 0.78 | | 0.32 | 1.91 | 2 |
| ADI Quartile (4 vs 1) | 1.00 | 4.00 | | 0.19 | 0.44 | -0.67 | 1.05 | 1 |
| Odds Ratio | 1.00 | 4.00 | | 1.21 | | 0.51 | 2.86 | 2 |

The adjusted OR (95% CI) for a subject in Bridge Clinic to attend at least one Bridge Clinic visit during the 16-week follow up vs a subject in usual care is 287.163 (36.688-2247.691).

# Post Hoc Analyses

## Binary logistic regression for readmissions, ED visits, and readmission or ED visit

### Readmission: yes/no within 16 week follow up

*Unadjusted*

- Outcome: Readmission (yes/no)
- Predictor: Group assignment (Bridge Clinic or usual care)
- Model: binary logistic regression
- Number of observations included in this analysis: 335

| | Low | High | Diff. | Effect | S.E. | Lower 0.95 | Upper 0.95 | Type |
|---|---|---|---|---|---|---|---|---|
| Group Assignment (Bridge Clinic vs Usual Care) | 1 | 2 | | 0.75 | 0.28 | 0.20 | 1.29 | 1 |
| Odds Ratio | 1 | 2 | | 2.11 | | 1.22 | 3.64 | 2 |

The unadjusted OR (95% CI) for a subject in Bridge Clinic to have a readmission within the 16 week follow up period vs a subject in usual care is 2.11 (1.223-3.64).

*Adjusted - Proportional odds model*

- Binary logistic regression model
- Single imputation was used to impute missing data for race and ethnicity
- Outcome: Readmission (yes/no)
- Predictor: Group assignment (Bridge Clinic or usual care)
- Adjusted for age, race, ethnicity, area deprivation index (quartiles)
  - Restricted cubic splines are used for continuous covariates (with 3 knots)
    - Age
- Number of observations included in this analysis: 335

| | Low | High | Diff. | Effect | S.E. | Lower 0.95 | Upper 0.95 | Type |
|---|---|---|---|---|---|---|---|---|
| Age | 31.93 | 45.72 | 13.79 | 0.27 | 0.24 | -0.21 | 0.74 | 1 |
| Odds Ratio | 31.93 | 45.72 | 13.79 | 1.30 | | 0.81 | 2.09 | 2 |
| Group Assignment (Bridge Clinic vs Usual Care) | 1.00 | 2.00 | | 0.74 | 0.28 | 0.19 | 1.30 | 1 |
| Odds Ratio | 1.00 | 2.00 | | 2.10 | | 1.21 | 3.67 | 2 |
| Race (Black or African American vs White) | 1.00 | 2.00 | | -0.63 | 0.52 | -1.65 | 0.38 | 1 |

| | Low | High | Diff. | Effect | S.E. | Lower 0.95 | Upper 0.95 | Type |
|---|---|---|---|---|---|---|---|---|
| Odds Ratio | 1.00 | 2.00 | | 0.53 | | 0.19 | 1.47 | 2 |
| Race (Other vs White) | 1.00 | 3.00 | | -7.21 | 33.33 | -72.55 | 58.12 | 1 |
| Odds Ratio | 1.00 | 3.00 | | 0.00 | | 0.00 | 1.743e+25 | 2 |
| Ethnicity (Hispanic/Latino vs Non-Hispanic/Latino) | 2.00 | 1.00 | | -7.38 | 31.33 | -68.80 | 54.03 | 1 |
| Odds Ratio | 2.00 | 1.00 | | 0.00 | | 0.00 | 2.918e+23 | 2 |
| ADI Quartile (2 vs 1) | 1.00 | 2.00 | | 0.21 | 0.37 | -0.53 | 0.94 | 1 |
| Odds Ratio | 1.00 | 2.00 | | 1.23 | | 0.59 | 2.56 | 2 |
| ADI Quartile (3 vs 1) | 1.00 | 3.00 | | -0.59 | 0.42 | -1.41 | 0.23 | 1 |
| Odds Ratio | 1.00 | 3.00 | | 0.56 | | 0.24 | 1.26 | 2 |
| ADI Quartile (4 vs 1) | 1.00 | 4.00 | | -0.06 | 0.36 | -0.77 | 0.64 | 1 |
| Odds Ratio | 1.00 | 4.00 | | 0.94 | | 0.46 | 1.90 | 2 |

The adjusted OR (95% CI) for a subject in Bridge Clinic to have a readmission within the 16 week follow up period vs a subject in usual care is 2.102 (1.205-3.666).

### ED visits: yes/no within 16 week follow up

*Unadjusted*

- Outcome: ED Visits (yes/no)
- Predictor: Group assignment (Bridge Clinic or usual care)
- Model: binary logistic regression
- Number of observations included in this analysis: 335

| | Low | High | Diff. | Effect | S.E. | Lower 0.95 | Upper 0.95 | Type |
|---|---|---|---|---|---|---|---|---|
| Group Assignment (Bridge Clinic vs Usual Care) | 1 | 2 | | 0.14 | 0.26 | -0.37 | 0.66 | 1 |
| Odds Ratio | 1 | 2 | | 1.15 | | 0.69 | 1.93 | 2 |

The unadjusted OR (95% CI) for a subject in Bridge Clinic to have an ED visit within the 16 week follow up period vs a subject in usual care is 1.155 (0.692-1.927).

*Adjusted - Proportional odds model*

- Binary logistic regression model
- Single imputation was used to impute missing data for race and ethnicity
- Outcome: ED Visit (yes/no)
- Predictor: Group assignment (Bridge Clinic or usual care)
- Adjusted for age, race, ethnicity, area deprivation index (quartiles)
  - Restricted cubic splines are used for continuous covariates (with 3 knots)
    - Age
- Number of observations included in this analysis: 335

| | Low | High | Diff. | Effect | S.E. | Lower 0.95 | Upper 0.95 | Type |
|---|---|---|---|---|---|---|---|---|
| Age | 31.93 | 45.72 | 13.79 | 0.42 | 0.24 | -0.04 | 0.88 | 1 |
| Odds Ratio | 31.93 | 45.72 | 13.79 | 1.52 | | 0.96 | 2.40 | 2 |
| Group Assignment (Bridge Clinic vs Usual Care) | 1.00 | 2.00 | | 0.15 | 0.27 | -0.37 | 0.68 | 1 |
| Odds Ratio | 1.00 | 2.00 | | 1.16 | | 0.69 | 1.97 | 2 |
| Race (Black or African American vs White) | 1.00 | 2.00 | | 0.53 | 0.40 | -0.26 | 1.31 | 1 |
| Odds Ratio | 1.00 | 2.00 | | 1.69 | | 0.77 | 3.72 | 2 |
| Race (Other vs White) | 1.00 | 3.00 | | -0.37 | 1.22 | -2.77 | 2.02 | 1 |
| Odds Ratio | 1.00 | 3.00 | | 0.69 | | 0.06 | 7.57 | 2 |
| Ethnicity (Hispanic/Latino vs Non-Hispanic/Latino) | 2.00 | 1.00 | | -0.86 | 1.20 | -3.21 | 1.48 | 1 |
| Odds Ratio | 2.00 | 1.00 | | 0.42 | | 0.04 | 4.41 | 2 |
| ADI Quartile (2 vs 1) | 1.00 | 2.00 | | 0.14 | 0.35 | -0.54 | 0.83 | 1 |
| Odds Ratio | 1.00 | 2.00 | | 1.15 | | 0.58 | 2.28 | 2 |
| ADI Quartile (3 vs 1) | 1.00 | 3.00 | | -0.66 | 0.40 | -1.44 | 0.12 | 1 |
| Odds Ratio | 1.00 | 3.00 | | 0.52 | | 0.24 | 1.13 | 2 |
| ADI Quartile (4 vs 1) | 1.00 | 4.00 | | -0.45 | 0.37 | -1.17 | 0.27 | 1 |
| Odds Ratio | 1.00 | 4.00 | | 0.64 | | 0.31 | 1.31 | 2 |

The adjusted OR (95% CI) for a subject in Bridge Clinic to have an ED visit within the 16 week follow up period vs a subject in usual care is 1.165 (0.687-1.974).

## Readmission or ED visit: yes/no within 16 week follow up

*Unadjusted*

- Outcome: Readmission or ED visit (yes/no)
- Predictor: Group assignment (Bridge Clinic or usual care)
- Model: binary logistic regression
- Number of observations included in this analysis: 335

| | Low | High | Diff. | Effect | S.E. | Lower 0.95 | Upper 0.95 | Type |
|---|---|---|---|---|---|---|---|---|
| Group Assignment (Bridge Clinic vs Usual Care) | 1 | 2 | | 0.45 | 0.23 | 0 | 0.90 | 1 |
| Odds Ratio | 1 | 2 | | 1.57 | | 1 | 2.46 | 2 |

The unadjusted OR (95% CI) for a subject in Bridge Clinic to have a readmission or ED visit within the 16 week follow up period vs a subject in usual care is 1.571 (1.002-2.462).

*Adjusted - Proportional odds model*

- Binary logistic regression model
- Single imputation was used to impute missing data for race and ethnicity
- Outcome: Readmission or ED visit (yes/no)
- Predictor: Group assignment (Bridge Clinic or usual care)
- Adjusted for age, race, ethnicity, area deprivation index (quartiles)
  - Restricted cubic splines are used for continuous covariates (with 3 knots)
    - Age
- Number of observations included in this analysis: 335

| | Low | High | Diff. | Effect | S.E. | Lower 0.95 | Upper 0.95 | Type |
|---|---|---|---|---|---|---|---|---|
| Age | 31.93 | 45.72 | 13.79 | 0.28 | 0.20 | -0.11 | 0.67 | 1 |
| Odds Ratio | 31.93 | 45.72 | 13.79 | 1.32 | | 0.89 | 1.95 | 2 |
| Group Assignment (Bridge Clinic vs Usual Care) | 1.00 | 2.00 | | 0.48 | 0.24 | 0.01 | 0.94 | 1 |
| Odds Ratio | 1.00 | 2.00 | | 1.61 | | 1.01 | 2.57 | 2 |
| Race (Black or African American vs White) | 1.00 | 2.00 | | 0.17 | 0.38 | -0.58 | 0.91 | 1 |
| Odds Ratio | 1.00 | 2.00 | | 1.18 | | 0.56 | 2.49 | 2 |
| Race (Other vs White) | 1.00 | 3.00 | | -1.00 | 1.19 | -3.33 | 1.33 | 1 |

| | Low | High | Diff. | Effect | S.E. | Lower 0.95 | Upper 0.95 | Type |
|---|---|---|---|---|---|---|---|---|
| Odds Ratio | 1.00 | 3.00 | | 0.37 | | 0.04 | 3.79 | 2 |
| Ethnicity (Hispanic/Latino vs Non-Hispanic/Latino) | 2.00 | 1.00 | | -1.47 | 1.16 | -3.74 | 0.80 | 1 |
| Odds Ratio | 2.00 | 1.00 | | 0.23 | | 0.02 | 2.23 | 2 |
| ADI Quartile (2 vs 1) | 1.00 | 2.00 | | 0.04 | 0.32 | -0.58 | 0.66 | 1 |
| Odds Ratio | 1.00 | 2.00 | | 1.04 | | 0.56 | 1.94 | 2 |
| ADI Quartile (3 vs 1) | 1.00 | 3.00 | | -0.98 | 0.35 | -1.67 | -0.30 | 1 |
| Odds Ratio | 1.00 | 3.00 | | 0.37 | | 0.19 | 0.74 | 2 |
| ADI Quartile (4 vs 1) | 1.00 | 4.00 | | -0.50 | 0.31 | -1.12 | 0.11 | 1 |
| Odds Ratio | 1.00 | 4.00 | | 0.60 | | 0.33 | 1.12 | 2 |

The adjusted OR (95% CI) for a subject in Bridge Clinic to have a readmission or ED visit within the 16 week follow up period vs a subject in usual care is 1.611 (1.011-2.568).

## Exploratory outcome: Index hospital length of stay from time of randomization

Table 7: LOS from time of randomization

| | Overall | Bridge Clinic | Usual Care |
|---|---|---|---|
| n | 335 | 167 | 168 |
| Length of stay from randomization (hours) (mean (SD)) | 125.8 (181.6) | 120.8 (170.2) | 130.7 (192.6) |
| Length of stay from randomization (hours) (median [IQR]) | 56.4 [27.5, 148.3] | 56.4 [26.3, 145.7] | 57.0 [28.2, 151.2] |

Figure 6: Index hospital length of stay from randomization, overall and by group

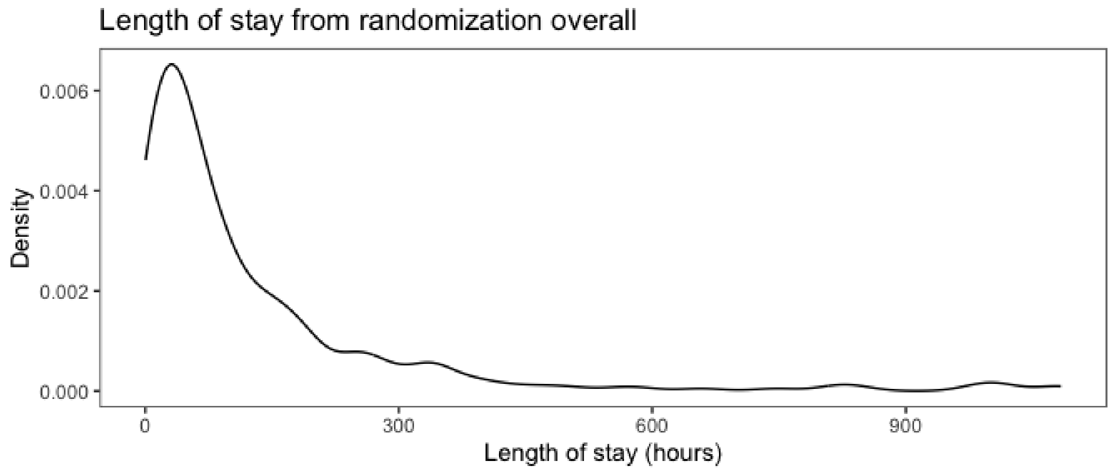


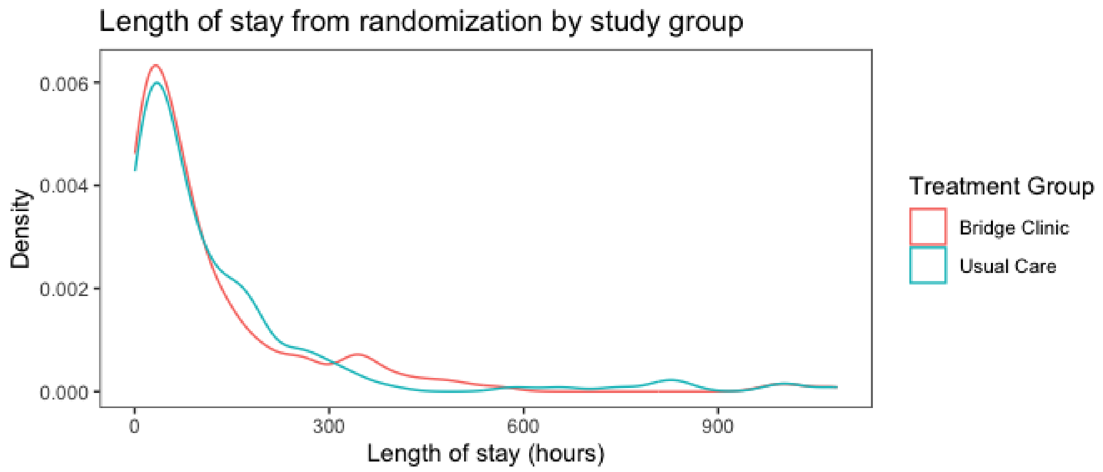


### Analysis

- Since the data are positive and skewed, a proportional odds model is used

*Unadjusted*

- Outcome: index hospital length of stay from time of randomization (hours)

- Predictor: Group assignment (Bridge Clinic or usual care)
- Number of observations included in this analysis: 335

| | Low | High | Diff. | Effect | S.E. | Lower 0.95 | Upper 0.95 | Type |
|---|---|---|---|---|---|---|---|---|
| Group Assignment (Bridge Clinic vs Usual Care) | 2 | 1 | | -0.09 | 0.19 | -0.47 | 0.28 | 1 |
| Odds Ratio | 2 | 1 | | 0.91 | | 0.63 | 1.32 | 2 |

The unadjusted OR (95% CI) for the index hospital length of stay being longer for a subject in Bridge Clinic vs Usual Care is 0.91 (0.628-1.319), p = 0.618.

*Adjusted*

**Multiple Imputation**

- Multiple imputation was used to impute missing data for race and ethnicity

**Proportional Odds Model**

- Outcome: index hospital length of stay (hours)
- Predictor: Group assignment (Bridge Clinic or usual care)
- Adjusted for age, race, ethnicity, area deprivation index (quartiles)
  - Restricted cubic splines are used for continuous covariates (with 3 knots)
    - Age
- Number of observations included in this analysis: 335

| | Low | High | Diff. | Effect | S.E. | Lower 0.95 | Upper 0.95 | Type |
|---|---|---|---|---|---|---|---|---|
| Age | 31.93 | 45.72 | 13.79 | 0.26 | 0.16 | -0.05 | 0.58 | 1 |
| Odds Ratio | 31.93 | 45.72 | 13.79 | 1.30 | | 0.95 | 1.79 | 2 |
| Group Assignment (Bridge Clinic vs Usual Care) | 2.00 | 1.00 | | -0.06 | 0.19 | -0.44 | 0.31 | 1 |
| Odds Ratio | 2.00 | 1.00 | | 0.94 | | 0.65 | 1.36 | 2 |
| Race (Black or African American vs White) | 1.00 | 2.00 | | -0.13 | 0.31 | -0.75 | 0.48 | 1 |
| Odds Ratio | 1.00 | 2.00 | | 0.88 | | 0.47 | 1.62 | 2 |
| Race (Other vs White) | 1.00 | 3.00 | | -0.44 | 0.72 | -1.84 | 0.96 | 1 |
| Odds Ratio | 1.00 | 3.00 | | 0.64 | | 0.16 | 2.62 | 2 |

| | Low | High | Diff. | Effect | S.E. | Lower 0.95 | Upper 0.95 | Type |
|---|---|---|---|---|---|---|---|---|
| Ethnicity (Hispanic/Latino vs Non-Hispanic/Latino) | 2.00 | 1.00 | | 0.75 | 0.66 | -0.55 | 2.05 | 1 |
| Odds Ratio | 2.00 | 1.00 | | 2.13 | | 0.58 | 7.80 | 2 |
| ADI Quartile (2 vs 1) | 1.00 | 2.00 | | 0.15 | 0.26 | -0.37 | 0.66 | 1 |
| Odds Ratio | 1.00 | 2.00 | | 1.16 | | 0.69 | 1.94 | 2 |
| ADI Quartile (3 vs 1) | 1.00 | 3.00 | | 0.12 | 0.26 | -0.39 | 0.63 | 1 |
| Odds Ratio | 1.00 | 3.00 | | 1.12 | | 0.67 | 1.88 | 2 |
| ADI Quartile (4 vs 1) | 1.00 | 4.00 | | 0.08 | 0.26 | -0.42 | 0.59 | 1 |
| Odds Ratio | 1.00 | 4.00 | | 1.09 | | 0.66 | 1.80 | 2 |

The adjusted OR (95% CI) for the index hospital length of stay being longer for a subject in Bridge Clinic vs Usual Care is 0.938 (0.646-1.364), p = 0.739.

## Propensity Score Analysis

- 16-week follow up for secondary outcomes has a poor response rate
- We see a difference in rates of follow up between treatment arms (subjects in Bridge Clinic had a better response rate)
- Estimate the propensity score using IPTW for the probability of following up
  - Include age, race, ethnicity, deprivation index (same variables used in adjusted models)
- 16-week follow up (yes/no):
  - Considered not to have followed up if they are missing all of the following:
    - Linkage to MOUD provider: yes/no
    - Recurrent opioid use: yes/no
    - Opioid use in the last 30 days
    - Self-reported buprenorphine-naloxone (or naltrexone) prescriptions filled
    - Overdose: yes/no
    - Quality of Life
- N = 247 with no 16-week follow up

**SMDs between groups**

```
##                                        Stratified by follow_up
##                                         No             Yes
SMD
##   n                                     333.91         335.30
##   AGE (mean (SD))                        39.91 (9.96)   39.81 (10.54)
0.009
##   RACE_2 (%)
0.023
##      White                              289.2 (86.6)   289.4 (86.3)
##      Black or African American           36.2 (10.9)    36.0 (10.8)
##      Other                                8.5 ( 2.6)     9.8 ( 2.9)
##   ETHNICITY = Non-Hispanic/Latino (%)  320.9 (96.1)   323.2 (96.4)
0.016
##   quartile_grp (%)
0.032
##      Quartile 1                         107.6 (32.2)   111.5 (33.2)
##      Quartile 2                          73.5 (22.0)    73.2 (21.8)
##      Quartile 3                          75.0 (22.5)    71.4 (21.3)
##      Quartile 4                          77.7 (23.3)    79.3 (23.6)
```

## Linkage to MOUD Provider

- Binary logistic regression model
- Single imputation was used to impute missing data for race and ethnicity
- Outcome: Linkage to MOUD provider (yes/no)
- Predictor: Group assignment (Bridge Clinic or usual care)
- Adjusted for age, race, ethnicity, area deprivation index (quartiles)
    - Restricted cubic splines are used for continuous covariates (with 3 knots)
        - Age
- Adjusted for propensity score weights
- Number of observations included in this analysis: 88

| | Low | High | Diff. | Effect | S.E. | Lower 0.95 | Upper 0.95 | Type |
|---|---|---|---|---|---|---|---|---|
| Age | 31.93 | 45.72 | 13.79 | 0.53 | 0.27 | 0.00 | 1.05 | 1 |
| Odds Ratio | 31.93 | 45.72 | 13.79 | 1.70 | | 1.00 | 2.87 | 2 |
| Group Assignment (Bridge Clinic vs Usual Care) | 1.00 | 2.00 | | 0.86 | 0.30 | 0.28 | 1.45 | 1 |
| Odds Ratio | 1.00 | 2.00 | | 2.37 | | 1.32 | 4.26 | 2 |
| Race (Black or African American vs White) | 1.00 | 2.00 | | -1.75 | 0.43 | -2.58 | -0.91 | 1 |
| Odds Ratio | 1.00 | 2.00 | | 0.17 | | 0.08 | 0.40 | 2 |
| Race (Other vs White) | 1.00 | 3.00 | | 16.63 | 44.73 | -71.03 | 104.29 | 1 |
| Odds Ratio | 1.00 | 3.00 | | 1.667e+07 | | 0.00 | 1.968e+45 | 2 |
| Ethnicity (Hispanic/Latino vs Non-Hispanic/Latino) | 2.00 | 1.00 | | -8.49 | 32.26 | -71.71 | 54.73 | 1 |
| Odds Ratio | 2.00 | 1.00 | | 0.00 | | 0.00 | 5.882e+23 | 2 |
| ADI Quartile (2 vs 1) | 1.00 | 2.00 | | 0.91 | 0.40 | 0.12 | 1.71 | 1 |
| Odds Ratio | 1.00 | 2.00 | | 2.49 | | 1.13 | 5.51 | 2 |
| ADI Quartile (3 vs 1) | 1.00 | 3.00 | | -0.51 | 0.37 | -1.24 | 0.21 | 1 |
| Odds Ratio | 1.00 | 3.00 | | 0.60 | | 0.29 | 1.24 | 2 |

| | Low | High | Diff. | Effect | S.E. | Lower 0.95 | Upper 0.95 | Type |
|---|---|---|---|---|---|---|---|---|
| ADI Quartile (4 vs 1) | 1.00 | 4.00 | | 0.16 | 0.37 | -0.55 | 0.88 | 1 |
| Odds Ratio | 1.00 | 4.00 | | 1.18 | | 0.57 | 2.42 | 2 |

The adjusted OR (95% CI) for a subject in Bridge Clinic to attend at least one visit with a MOUD provider in the 16-week follow-up vs a subject in usual care is 2.371 (1.321-4.256).

## Recurrent opioid use

- Binary logistic regression model
- Single imputation was used to impute missing data for race and ethnicity
- Outcome: recurrent opioid use (yes/no)
- Predictor: Group assignment (Bridge Clinic or usual care)
- Adjusted for age, race, ethnicity, area deprivation index (quartiles)
  - Restricted cubic splines are used for continuous covariates (with 3 knots)
    - Age
- Adjusted for propensity score weights
- Number of observations included in this analysis: 88

| | Low | High | Diff. | Effect | S.E. | Lower 0.95 | Upper 0.95 | Type |
|---|---|---|---|---|---|---|---|---|
| Age | 31.93 | 45.72 | 13.79 | 0.04 | 0.23 | -0.42 | 0.50 | 1 |
| Odds Ratio | 31.93 | 45.72 | 13.79 | 1.04 | | 0.66 | 1.64 | 2 |
| Group Assignment (Bridge Clinic vs Usual Care) | 1.00 | 2.00 | | -0.36 | 0.27 | -0.88 | 0.16 | 1 |
| Odds Ratio | 1.00 | 2.00 | | 0.70 | | 0.42 | 1.18 | 2 |
| Race (Black or African American vs White) | 1.00 | 2.00 | | 1.16 | 0.40 | 0.38 | 1.95 | 1 |
| Odds Ratio | 1.00 | 2.00 | | 3.20 | | 1.46 | 7.03 | 2 |
| Race (Other vs White) | 1.00 | 3.00 | | 1.86 | 0.95 | -0.01 | 3.73 | 1 |
| Odds Ratio | 1.00 | 3.00 | | 6.42 | | 0.99 | 41.49 | 2 |
| Ethnicity (Hispanic/Latino vs Non-Hispanic/Latino) | 2.00 | 1.00 | | -1.45 | 0.87 | -3.16 | 0.26 | 1 |
| Odds Ratio | 2.00 | 1.00 | | 0.23 | | 0.04 | 1.30 | 2 |
| ADI Quartile (2 vs 1) | 1.00 | 2.00 | | -0.27 | 0.33 | -0.91 | 0.37 | 1 |
| Odds Ratio | 1.00 | 2.00 | | 0.76 | | 0.40 | 1.45 | 2 |
| ADI Quartile (3 vs 1) | 1.00 | 3.00 | | 0.68 | 0.33 | 0.02 | 1.33 | 1 |
| Odds Ratio | 1.00 | 3.00 | | 1.97 | | 1.02 | 3.79 | 2 |

| | Low | High | Diff. | Effect | S.E. | Lower 0.95 | Upper 0.95 | Type |
|---|---|---|---|---|---|---|---|---|
| ADI Quartile (4 vs 1) | 1.00 | 4.00 | | -0.62 | 0.33 | -1.27 | 0.03 | 1 |
| Odds Ratio | 1.00 | 4.00 | | 0.54 | | 0.28 | 1.03 | 2 |

The adjusted OR (95% CI) for a subject in Bridge Clinic to have recurrent opioid use in the 16-week follow-up vs a subject in usual care is 0.699 (0.416-1.175).

## Opioid use in last 30 days

- Proportional odds model
- Single imputation was used to impute missing data for race and ethnicity
- Outcome: opioid use in past 30 days (no use, single use, multiple use)
- Predictor: Group assignment (Bridge Clinic or usual care)
- Adjusted for age, race, ethnicity, area deprivation index (quartiles)
  - Restricted cubic splines are used for continuous covariates (with 3 knots)
    - Age
- Adjusted for propensity score weights
  - This model was not adjusted for ethnicity because all subjects in this model (N = 32) are Non-Hispanic/Latino
- Number of observations included in this analysis: 32

| | Low | High | Diff. | Effect | S.E. | Lower 0.95 | Upper 0.95 | Type |
|---|---|---|---|---|---|---|---|---|
| Age | 31.93 | 45.72 | 13.79 | 2.49 | 0.60 | 1.32 | 3.67 | 1 |
| Odds Ratio | 31.93 | 45.72 | 13.79 | 12.08 | | 3.73 | 39.10 | 2 |
| Group Assignment (Bridge Clinic vs Usual Care) | 1.00 | 2.00 | | 3.85 | 0.83 | 2.23 | 5.47 | 1 |
| Odds Ratio | 1.00 | 2.00 | | 46.94 | | 9.28 | 237.35 | 2 |
| Race (Black or African American vs White) | 1.00 | 2.00 | | 3.30 | 1.04 | 1.26 | 5.34 | 1 |
| Odds Ratio | 1.00 | 2.00 | | 27.22 | | 3.54 | 209.44 | 2 |
| Race (Other vs White) | 1.00 | 3.00 | | 10.33 | 47.89 | -83.54 | 104.20 | 1 |
| Odds Ratio | 1.00 | 3.00 | | 30,712.09 | | 0.00 | 1.794e+45 | 2 |
| ADI Quartile (2 vs 1) | 1.00 | 2.00 | | 0.33 | 0.80 | -1.24 | 1.90 | 1 |
| Odds Ratio | 1.00 | 2.00 | | 1.39 | | 0.29 | 6.66 | 2 |
| ADI Quartile (3 vs 1) | 1.00 | 3.00 | | -1.18 | 0.87 | -2.89 | 0.53 | 1 |
| Odds Ratio | 1.00 | 3.00 | | 0.31 | | 0.06 | 1.70 | 2 |

| | Low | High | Diff. | Effect | S.E. | Lower 0.95 | Upper 0.95 | Type |
|---|---|---|---|---|---|---|---|---|
| ADI Quartile (4 vs 1) | 1.00 | 4.00 | | 9.66 | 24.10 | -37.59 | 56.90 | 1 |
| Odds Ratio | 1.00 | 4.00 | | 15,605.84 | | 0.00 | 5.132e+24 | 2 |

The adjusted OR (95% CI) for a subject in Bridge Clinic to have more opioid uses in the past 30 days vs a subject in usual care is 46.943 (9.284-237.351).

## Overdose

- Binary logistic regression model
- Single imputation was used to impute missing data for race and ethnicity
- Outcome: Overdose (yes/no)
- Predictor: Group assignment (Bridge Clinic or usual care)
- Adjusted for age, race, ethnicity, area deprivation index (quartiles)
  - Restricted cubic splines are used for continuous covariates (with 3 knots)
    - Age
- Adjusted for propensity score weights
- Number of observations included in this analysis: 86

| | Low | High | Diff. | Effect | S.E. | Lower 0.95 | Upper 0.95 | Type |
|---|---|---|---|---|---|---|---|---|
| Age | 31.93 | 45.72 | 13.79 | -1.27 | 0.87 | -2.97 | 0.44 | 1 |
| Odds Ratio | 31.93 | 45.72 | 13.79 | 0.28 | | 0.05 | 1.55 | 2 |
| Group Assignment (Bridge Clinic vs Usual Care) | 1.00 | 2.00 | | -2.18 | 0.66 | -3.48 | -0.88 | 1 |
| Odds Ratio | 1.00 | 2.00 | | 0.11 | | 0.03 | 0.41 | 2 |
| Race (Black or African American vs White) | 1.00 | 2.00 | | -0.28 | 0.66 | -1.57 | 1.01 | 1 |
| Odds Ratio | 1.00 | 2.00 | | 0.76 | | 0.21 | 2.74 | 2 |
| Race (Other vs White) | 1.00 | 3.00 | | -4.23 | 95.07 | -190.57 | 182.10 | 1 |
| Odds Ratio | 1.00 | 3.00 | | 0.01 | | 0.00 | 1.219e+79 | 2 |
| Ethnicity (Hispanic/Latino vs Non-Hispanic/Latino) | 2.00 | 1.00 | | -9.78 | 90.11 | -186.39 | 166.83 | 1 |
| Odds Ratio | 2.00 | 1.00 | | 0.00 | | 0.00 | 2.827e+72 | 2 |
| ADI Quartile (2 vs 1) | 1.00 | 2.00 | | 0.88 | 0.71 | -0.51 | 2.27 | 1 |
| Odds Ratio | 1.00 | 2.00 | | 2.41 | | 0.60 | 9.68 | 2 |
| ADI Quartile (3 vs 1) | 1.00 | 3.00 | | 1.21 | 0.69 | -0.14 | 2.57 | 1 |
| Odds Ratio | 1.00 | 3.00 | | 3.37 | | 0.87 | 13.12 | 2 |

| | Low | High | Diff. | Effect | S.E. | Lower 0.95 | Upper 0.95 | Type |
|---|---|---|---|---|---|---|---|---|
| ADI Quartile (4 vs 1) | 1.00 | 4.00 | | -8.62 | 30.67 | -68.73 | 51.49 | 1 |
| Odds Ratio | 1.00 | 4.00 | | 0.00 | | 0.00 | 2.309e+22 | 2 |

The adjusted OR (95% CI) for a subject in Bridge Clinic to overdose during the 16-week follow-up vs a subject in usual care is 0.113 (0.031-0.414).

## Number of buprenorphine refills

- Proportional odds model
- Single imputation was used to impute missing data for race and ethnicity
- Outcome: Number of buprenorphine refills
- Predictor: Group assignment (Bridge Clinic or usual care)
- Adjusted for age, race, ethnicity, area deprivation index (quartiles)
    - Restricted cubic splines are used for continuous covariates (with 3 knots)
        - Age
- Adjusted for propensity score weights
- Number of observations included in this analysis: 77

| | Low | High | Diff. | Effect | S.E. | Lower 0.95 | Upper 0.95 | Type |
|---|---|---|---|---|---|---|---|---|
| Age | 31.93 | 45.72 | 13.79 | 0.62 | 0.23 | 0.17 | 1.07 | 1 |
| Odds Ratio | 31.93 | 45.72 | 13.79 | 1.86 | | 1.18 | 2.92 | 2 |
| Group Assignment (Bridge Clinic vs Usual Care) | 1.00 | 2.00 | | 1.82 | 0.26 | 1.31 | 2.33 | 1 |
| Odds Ratio | 1.00 | 2.00 | | 6.16 | | 3.69 | 10.30 | 2 |
| Race (Black or African American vs White) | 1.00 | 2.00 | | -1.57 | 0.35 | -2.26 | -0.89 | 1 |
| Odds Ratio | 1.00 | 2.00 | | 0.21 | | 0.10 | 0.41 | 2 |
| Race (Other vs White) | 1.00 | 3.00 | | -0.35 | 0.79 | -1.90 | 1.21 | 1 |
| Odds Ratio | 1.00 | 3.00 | | 0.71 | | 0.15 | 3.35 | 2 |
| Ethnicity (Hispanic/Latino vs Non-Hispanic/Latino) | 2.00 | 1.00 | | -1.14 | 0.87 | -2.85 | 0.58 | 1 |
| Odds Ratio | 2.00 | 1.00 | | 0.32 | | 0.06 | 1.78 | 2 |
| ADI Quartile (2 vs 1) | 1.00 | 2.00 | | 0.73 | 0.32 | 0.11 | 1.35 | 1 |
| Odds Ratio | 1.00 | 2.00 | | 2.07 | | 1.11 | 3.85 | 2 |
| ADI Quartile (3 vs 1) | 1.00 | 3.00 | | 0.17 | 0.32 | -0.45 | 0.80 | 1 |
| Odds Ratio | 1.00 | 3.00 | | 1.19 | | 0.64 | 2.23 | 2 |

| | Low | High | Diff. | Effect | S.E. | Lower 0.95 | Upper 0.95 | Type |
|---|---|---|---|---|---|---|---|---|
| ADI Quartile (4 vs 1) | 1.00 | 4.00 | | 0.18 | 0.29 | -0.40 | 0.75 | 1 |
| Odds Ratio | 1.00 | 4.00 | | 1.19 | | 0.67 | 2.12 | 2 |

The adjusted OR (95% CI) for the number of buprenorphine refills being greater for a subject in Bridge Clinic vs Usual Care is 6.165 (3.69-10.299).

## Quality of Life

- Proportional odds model
- Single imputation was used to impute missing data for race and ethnicity
- Outcome: quality of life score
- Predictor: Group assignment (Bridge Clinic or usual care)
- Adjusted for age, race, ethnicity, area deprivation index (quartiles)
  - Restricted cubic splines are used for continuous covariates (with 3 knots)
    - Age
- Adjusted for propensity score weights
- Number of observations included in this analysis: 86

| | Low | High | Diff. | Effect | S.E. | Lower 0.95 | Upper 0.95 | Type |
|---|---|---|---|---|---|---|---|---|
| Age | 31.93 | 45.72 | 13.79 | -0.48 | 0.21 | -0.89 | -0.08 | 1 |
| Odds Ratio | 31.93 | 45.72 | 13.79 | 0.62 | | 0.41 | 0.92 | 2 |
| Group Assignment (Bridge Clinic vs Usual Care) | 1.00 | 2.00 | | -0.11 | 0.22 | -0.54 | 0.32 | 1 |
| Odds Ratio | 1.00 | 2.00 | | 0.90 | | 0.58 | 1.38 | 2 |
| Race (Black or African American vs White) | 1.00 | 2.00 | | -1.06 | 0.30 | -1.64 | -0.47 | 1 |
| Odds Ratio | 1.00 | 2.00 | | 0.35 | | 0.19 | 0.62 | 2 |
| Race (Other vs White) | 1.00 | 3.00 | | -1.98 | 0.64 | -3.23 | -0.73 | 1 |
| Odds Ratio | 1.00 | 3.00 | | 0.14 | | 0.04 | 0.48 | 2 |
| Ethnicity (Hispanic/Latino vs Non-Hispanic/Latino) | 2.00 | 1.00 | | 0.66 | 0.63 | -0.57 | 1.89 | 1 |
| Odds Ratio | 2.00 | 1.00 | | 1.93 | | 0.56 | 6.62 | 2 |
| ADI Quartile (2 vs 1) | 1.00 | 2.00 | | -0.66 | 0.28 | -1.22 | -0.11 | 1 |
| Odds Ratio | 1.00 | 2.00 | | 0.51 | | 0.30 | 0.89 | 2 |
| ADI Quartile (3 vs 1) | 1.00 | 3.00 | | -0.73 | 0.30 | -1.32 | -0.14 | 1 |
| Odds Ratio | 1.00 | 3.00 | | 0.48 | | 0.27 | 0.87 | 2 |

| | Low | High | Diff. | Effect | S.E. | Lower 0.95 | Upper 0.95 | Type |
|---|---|---|---|---|---|---|---|---|
| ADI Quartile (4 vs 1) | 1.00 | 4.00 | | -0.28 | 0.28 | -0.82 | 0.26 | 1 |
| Odds Ratio | 1.00 | 4.00 | | 0.76 | | 0.44 | 1.30 | 2 |

The adjusted OR (95% CI) for the quality of life score being greater for a subject in Bridge Clinic vs Usual Care is 0.896 (0.583-1.376).
