# Supplementary material for: Effect of a Co-Located Bridging Recovery Initiative on Hospital Length of Stay Among Patients With Opioid Use Disorder: The BRIDGE Randomized Clinical Trial
Source: JAMA Netw Open. 2024 Feb 27;7(2):e2356430. doi: 10.1001/jamanetworkopen.2023.56430 (PMC10900965; doi:10.1001/jamanetworkopen.2023.56430)
Supplement: Supplement 2. — eAppendix. Methods and Design [file jamanetwopen-e2356430-s002.pdf]

Supplementary Online Content

Marcovitz D, Dear ML, Donald R, et al. Effect of a co-located bridging recovery initiative on hospital length of stay among patients with opioid use disorder: the BRIDGE randomized clinical trial. *JAMA Netw Open.* 2024;7(2):e2356430. doi:10.1001/jamanetworkopen.2023.56430

eAppendix. Methods and Design

This supplementary material has been provided by the authors to give readers additional information about their work.

© 2024 Marcovitz D et al. *JAMA Network Open.*

eAppendix. Methods and Design

Data Sources

This study used the electronic health record (EHR) as a real-world data source. In addition, we estimated the area deprivation index (ADI) as an indicator of social drivers of health using the Neighborhood Atlas tool.[24,25] Please see supplement. National level ADI was used for this study as not all patients lived in the same state. To minimize missingness and prevent bias due to unequal documentation of zip code, we derived a four-level ordinal indicator of social deprivation. ADI values were first divided into quartiles. Patients without an available ADI score were assigned to a quartile based on housing status obtained via manual chart review: those found to be domiciled were assigned to the second lowest quartile; those who were not domiciled were assigned to the lowest quartile.

Participant Assessment at Follow-up

At 16-weeks, patients were called to obtain information about attendance at outpatient MOUD follow-up, self-reported relapse to use of illicit opioids and frequency of use in the preceding 30 days, number of overdoses since discharge, intervention contamination, and a measure of overall psychological well-being (Schwartz Outcome Scale-10).[27]

At the 16-week follow up, case managers attempted at least three telephone calls, leaving voicemails if the patient did not respond. Prompted by a low response rate, beginning in August 2020 each call was accompanied by a text message from recovery coaches on their institutional mobile device or through our patient portal. Case managers attempted to engage additional contacts in the medical record when patients were unable to be reached. Some patients could not be reached for documented reasons at 16 weeks, such as incarceration or death.

© 2024 Marcovitz D et al. *JAMA Network Open.*
