# Supplementary material for: Effect of a Co-Located Bridging Recovery Initiative on Hospital Length of Stay Among Patients With Opioid Use Disorder: The BRIDGE Randomized Clinical Trial
Source: JAMA Netw Open. 2024 Feb 27;7(2):e2356430. doi: 10.1001/jamanetworkopen.2023.56430 (PMC10900965; doi:10.1001/jamanetworkopen.2023.56430)
Supplement: Supplement 3. — Nonauthor Collaborators [file jamanetwopen-e2356430-s003.pdf]

Supplemental Online Content: Nonauthor Collaborators
*First name, last name, and suffix (if applicable) are required and will appear in PubMed.

*Group Name(s): Vanderbilt Learning Healthcare System Platform Investigators							
***First Name and Middle Initial(s)**	***Last Name**	***Suffix (eg, Jr, III)**	Academic Degrees	Institution	Location (city, state/province, country)	Role or Contribution, eg, chair, principal investigator	Group (if more than 1 Group listed in the byline) and/or Subgroup (eg, Steering Committee)
Gordon R.	Bernard		MD	Vanderbilt University Medical Center and Vanderbilt University	Nashville, Tennessee		Vanderbilt Learning Healthcare System Platform Investigators
Robert S.	Dittus		MD, MPH	Vanderbilt University Medical Center and Vanderbilt University	Nashville, Tennessee		Vanderbilt Learning Healthcare System Platform Investigators
Shon	Dwyer		MBA, BSN, RN	Vanderbilt University Medical Center and Vanderbilt University	Nashville, Tennessee		Vanderbilt Learning Healthcare System Platform Investigators
Peter J.	Embi		MD, MS, FACP, FACM	Vanderbilt University Medical Center and Vanderbilt University	Nashville, Tennessee		Vanderbilt Learning Healthcare System Platform Investigators
Chad	Fitzgerald		JD	Vanderbilt University Medical Center and Vanderbilt University	Nashville, Tennessee		Vanderbilt Learning Healthcare System Platform Investigators
Robert E.	Freundlich		MD	Vanderbilt University Medical Center and Vanderbilt University	Nashville, Tennessee		Vanderbilt Learning Healthcare System Platform Investigators
Frank E.	Harrell	Jr.	PhD	Vanderbilt University Medical Center and Vanderbilt University	Nashville, Tennessee		Vanderbilt Learning Healthcare System Platform Investigators
Paul A.	Harris		Phd, PFACMI, FIAHSI	Vanderbilt University Medical Center and Vanderbilt University	Nashville, Tennessee		Vanderbilt Learning Healthcare System Platform Investigators
Tina	Hartert		MD, MPH	Vanderbilt University Medical Center and Vanderbilt University	Nashville, Tennessee		Vanderbilt Learning Healthcare System Platform Investigators
Jim	Hayman		MS, MBA	Vanderbilt University Medical Center and Vanderbilt University	Nashville, Tennessee		Vanderbilt Learning Healthcare System Platform Investigators
Catherine H.	Ivory		PhD, RN-BC, NEA-BC, FAAN	Vanderbilt University Medical Center and Vanderbilt University	Nashville, Tennessee		Vanderbilt Learning Healthcare System Platform Investigators
Ruth	Kleinpell		PhD, RN, FAAN	Vanderbilt University Medical Center and Vanderbilt University	Nashville, Tennessee		Vanderbilt Learning Healthcare System Platform Investigators
Sunil	Kripalani		MD, MSc	Vanderbilt University Medical Center and Vanderbilt University	Nashville, Tennessee		Vanderbilt Learning Healthcare System Platform Investigators
Lee Ann	Liska		MBA, RN	Vanderbilt University Medical Center and Vanderbilt University	Nashville, Tennessee		Vanderbilt Learning Healthcare System Platform Investigators
Patrick	Luther		MHS	Vanderbilt University Medical Center and Vanderbilt University	Nashville, Tennessee		Vanderbilt Learning Healthcare System Platform Investigators
Jay	Morrison		MSN, RN, CPPS	Vanderbilt University Medical Center and Vanderbilt University	Nashville, Tennessee		Vanderbilt Learning Healthcare System Platform Investigators
Thomas	Nantais		MBA, BSN, RN	Vanderbilt University Medical Center and Vanderbilt University	Nashville, Tennessee		Vanderbilt Learning Healthcare System Platform Investigators
Jill M.	Pulley		MBA	Vanderbilt University Medical Center and Vanderbilt University	Nashville, Tennessee		Vanderbilt Learning Healthcare System Platform Investigators

Supplemental Online Content: Nonauthor Collaborators
*First name, last name, and suffix (if applicable) are required and will appear in PubMed.

***First Name and Middle Initial(s)**	***Last Name**	***Suffix (eg, Jr, III)**	Academic Degrees	Institution	Location (city, state/province, country)	Role or Contribution, eg, chair, principal investigator	Group (if more than 1 Group listed in the byline) and/or Subgroup (eg, Steering Committee)
Kris	Rehm		MD	Vanderbilt University Medical Center and Vanderbilt University	Nashville, Tennessee		Vanderbilt Learning Healthcare System Platform Investigators
Russell L.	Rothman		MD, MPP	Vanderbilt University Medical Center and Vanderbilt University	Nashville, Tennessee		Vanderbilt Learning Healthcare System Platform Investigators
Patti	Runyan		DNP, MBA, RN, NEA-[illegible]	Vanderbilt University Medical Center and Vanderbilt University	Nashville, Tennessee		Vanderbilt Learning Healthcare System Platform Investigators
Wesley H.	Self		MD, MPH	Vanderbilt University Medical Center and Vanderbilt University	Nashville, Tennessee		Vanderbilt Learning Healthcare System Platform Investigators
Matthew W.	Semler		MD, MSc	Vanderbilt University Medical Center and Vanderbilt University	Nashville, Tennessee		Vanderbilt Learning Healthcare System Platform Investigators
Robin	Steaban		MSN, RN, NEA-BC	Vanderbilt University Medical Center and Vanderbilt University	Nashville, Tennessee		Vanderbilt Learning Healthcare System Platform Investigators
Cosby A.	Stone	Jr.	MD, MPH	Vanderbilt University Medical Center and Vanderbilt University	Nashville, Tennessee		Vanderbilt Learning Healthcare System Platform Investigators
Philip D.	Walker		MLIS, MSHI	Vanderbilt University Medical Center and Vanderbilt University	Nashville, Tennessee		Vanderbilt Learning Healthcare System Platform Investigators
Consuelo H.	Wilkins		MD, MSCI	Vanderbilt University Medical Center and Vanderbilt University	Nashville, Tennessee		Vanderbilt Learning Healthcare System Platform Investigators
Adam	Wright		PhD	Vanderbilt University Medical Center and Vanderbilt University	Nashville, Tennessee		Vanderbilt Learning Healthcare System Platform Investigators
Autumn D.	Zuckerman		PharmD	Vanderbilt University Medical Center and Vanderbilt University	Nashville, Tennessee		Vanderbilt Learning Healthcare System Platform Investigators
