# Supplementary material for: Effect of a Co-Located Bridging Recovery Initiative on Hospital Length of Stay Among Patients With Opioid Use Disorder: The BRIDGE Randomized Clinical Trial
Source: JAMA Netw Open. 2024 Feb 27;7(2):e2356430. doi: 10.1001/jamanetworkopen.2023.56430 (PMC10900965; doi:10.1001/jamanetworkopen.2023.56430)
Supplement: Supplement 4. — Data Sharing Statement [file jamanetwopen-e2356430-s004.pdf]

Data Sharing Statement

Marcovitz. Effect of a Co-Located Bridging Recovery Initiative on Hospital Length of Stay Among Patients With Opioid Use Disorder. *JAMA Netw Open*. Published February 27, 2024. doi:10.1001/jamanetworkopen.2023.56430

Data
Data available: Yes
Data types: Deidentified participant data
How to access data: david.marcovitz@vumc.org
When available: With publication

Supporting Documents
Document types: None

Additional Information
Who can access the data: Researchers whose proposed use of the data has been approved by the study team and following appropriate regulatory approvals.
Types of analyses: For any purpose
Mechanisms of data availability: With a signed data access agreement
